# Roles of Genetic Polymorphisms in the Folate Pathway in Childhood Acute Lymphoblastic Leukemia Evaluated by Bayesian Relevance and Effect Size Analysis

**DOI:** 10.1371/journal.pone.0069843

**Published:** 2013-08-05

**Authors:** Orsolya Lautner-Csorba, András Gézsi, Dániel J. Erdélyi, Gábor Hullám, Péter Antal, Ágnes F. Semsei, Nóra Kutszegi, Gábor Kovács, András Falus, Csaba Szalai

**Affiliations:** 1 Department of Genetics, Cell- and Immunobiology, Semmelweis University, Budapest, Hungary; 2 2nd Department of Pediatrics, Semmelweis University, Budapest, Hungary; 3 Department of Measurement and Information Systems, University of Technology and Economics, Budapest, Hungary; 4 Heim Pal Children Hospital, Budapest, Hungary; 5 Csertex Research Laboratory, Budapest, Hungary; Huazhong University of Science and Technology, China

## Abstract

In this study we investigated whether polymorphisms in the folate pathway influenced the risk of childhood acute lymphoblastic leukemia (ALL) or the survival rate of the patients. For this we selected and genotyped 67 SNPs in 15 genes in the folate pathway in 543 children with ALL and 529 controls. The results were evaluated by gender adjusted logistic regression and by the Bayesian network based Bayesian multilevel analysis of relevance (BN-BMLA) methods. Bayesian structure based odds ratios for the relevant variables and interactions were also calculated. Altogether 9 SNPs in 8 genes were associated with altered susceptibility to ALL. After correction for multiple testing, two associations remained significant. The genotype distribution of the *MTHFD1* rs1076991 differed significantly between the ALL and control population. Analyzing the subtypes of the disease the GG genotype increased only the risk of B-cell ALL (p = 3.52×10^−4^; OR = 2.00). The GG genotype of the rs3776455 SNP in the *MTRR* gene was associated with a significantly reduced risk to ALL (p = 1.21×10^−3^; OR = 0.55), which resulted mainly from the reduced risk to B-cell and hyperdiploid-ALL. The TC genotype of the rs9909104 SNP in the *SHMT1* gene was associated with a lower survival rate comparing it to the TT genotype (80.2% vs. 88.8%; p = 0.01). The BN-BMLA confirmed the main findings of the frequentist-based analysis and showed structural interactional maps and the probabilities of the different structural association types of the relevant SNPs especially in the hyperdiploid-ALL, involving additional SNPs in genes like *TYMS*, *DHFR* and *GGH*. We also investigated the statistical interactions and redundancies using structural model properties. These results gave further evidence that polymorphisms in the folate pathway could influence the ALL risk and the effectiveness of the therapy. It was also shown that in gene association studies the BN-BMLA could be a useful supplementary to the traditional frequentist-based statistical method.

## Introduction

Acute lymphoblastic leukemia (ALL) is the most frequent haematopoetic malignancy in childhood worldwide [1], and also in Hungary with about 60–80 new cases registered a year (Hungarian Children Cancer Registry). In the last years several genome wide and candidate gene association studies have tried to explore the genetic background of the disease, and revealed a number of genes and genetic variations, which might influence the risk of the disease or the response to the therapy [2], [3]. But these results are often inconsistent and require further studies for confirmation.

**Figure 1:**
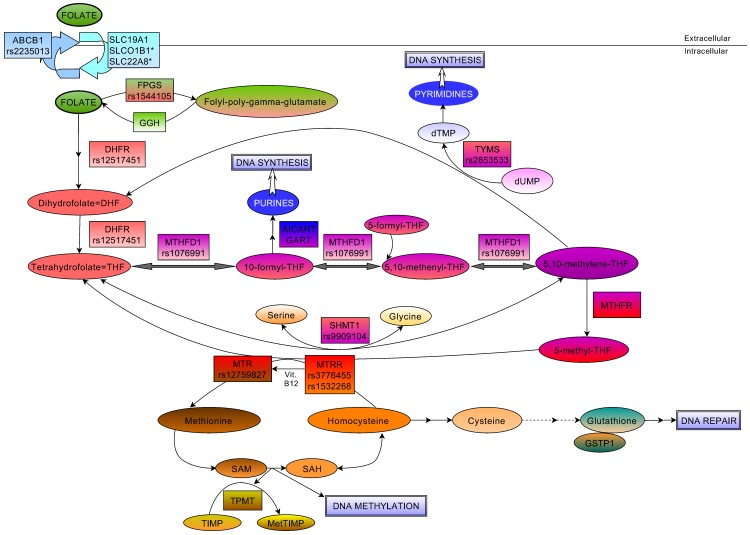
Schematic overview of the intracellular folate pathway. Key molecules and derivatives of the pathway are denoted as ovals, regulatory enzymes as rectangles, affected DNA mechanisms as double rectangles. Genetic polymorphisms are showed in rectangles only where significant results were found. dUMP = Deoxyuridine monophosphate, dTMP = Deoxythimidine monophosphate, Vit.B12 = Vitamin B12, SAM = S-adenosylmethionine, SAH = S-adenosylhomocysteine, TIMP = Thioinosine monophosphate, MetTIMP = Methyl-thioinosine monophosphate, AICART = AICAR (5-Aminoimidazole-4-carboxamide ribonucleotide) formyltransferase, GART = GAR (glycinamide ribonucleotide) formyltransferase.

It is well known that genetic alterations of genes in DNA synthesis or in DNA methylation could be a first step for haematopoetic (lymphoid) malignant cell production [4], [5]. It is necessary to maintain the adequate concentration of folate (tetrahidrofolate-THF) because folate provides a methyl group for the proper DNA synthesis, moreover for homocysteine in the methionine cycle, which is important in the DNA methylation process [6], [7]. Genetic variations of genes encoding pivotal regulator and transport enzymes of the folate cycle (e.g. *MTHFD1*, *MTRR*, *MTR, MTHFR, DHFR, GGH, SLCO1B1*) could influence the available folate in the cells, and therefore might alter the susceptibility to ALL and the response to the therapy [7]–[12]. The objective of this study was to investigate, whether genetic polymorphisms in genes related to folate metabolic pathway influence the risk to childhood ALL. On the other hand, as the folate pathway is one of the main targets of the ALL chemotherapy we also studied, whether these variations influence the overall and event-free survival of the patients after the therapy. For this, we selected 67 SNPs in 15 genes (*DHFR, MTHFD1, MTHFR, MTRR, MTR, SHMT1, TYMS, ABCB1, FPGS, GGH, GSTP1, SLCO1B1, SLC19A1, SLC22A8, TPMT)* in the folate metabolic pathway (Figure 1 and Table S1).

Recently, several methods have been described and used for evaluating multiple interactions in multifactorial diseases (for the comparison of methods capable of capturing interactions see [13], [14]. In the present study, for the statistical analysis we applied a traditional frequentist method (logistic regression), a multifactor dimensionality reduction (MDR, [15]) tool and a Bayesian statistical methodology, named Bayesian network based Bayesian multilevel analysis of relevance (BN-BMLA), which extends association analysis by estimating posteriors [16]–[18]of strong relevance. By analyzing the data with BN-BMLA we intend to refine the dependency relationships of factors, e.g. we investigate whether a variable is directly relevant or its association is only mediated.BN-BMLA was recently extended with Bayesian effect size estimation providing a hybrid measure, the Bayesian structure based odds ratio, which characterizes the parametric and strong relevance aspects of factors [19].

In previous studies, we showed that the Bayesian networks offer a rich language in the Bayesian statistical framework for the representation of relevance types, including causal, acausal, and multitarget aspects. We demonstrated that BN-BMLA can be a useful supplementary to the traditional frequentist statistical methods in gene association studies [20], [21].

## Methods

### Study population

The study population consisted of 543 childhood acute lymphoid leukemia (ALL) patients of Hungarian (Caucasian) origin treated according to the ALL Berlin-Frankfurt-Münster (BFM) 90, 95 or ALL IC-BFM 2002 chemotherapy protocols in ten Hungarian centres. The patients were diagnosed with ALL between 1990 and 2010. See more details about the characteristics of patients in Table 1. The median age of ALL patients was 5.1 (1–18) years at diagnosis. Peripheral blood samples were taken from children with ALL in remission phase. Only those samples were involved in the study, where the normal leukocyte cell count was≥5 G/l measured by flow cytometry. In fact, we analyzed only normal leukocytes and germline polymorphisms. There is no significant difference in the distribution of age groups or genders or ALL-immunophenotypes between the whole ALL children population in the Hungarian Children Cancer Registry and our sample collection.

**Table 1:** Characteristics of cases and controls.

	Cases	Controls
**Gender (%)**		
Male	308 (56.7)	305 (57.7)
Female	235 (43.3)	224 (42.3)
**Years of age at diagnosis**		
Mean (±SD)	6.4(±4.2)	16.1(±12.4)
Median (Range)	5.1(1–18)	12.5(1.5–59)
**Risk category (%)**		
Low risk, LR	96 (17.7)	-
Medium risk, MR	309 (56.9)	-
High risk, HR	55 (10.1)	-
**Morphological subgroup (%)**		
B-ALL	390 (71.8)	-
T-ALL	78 (14.4)	-
**Cytogenetics**		
Hyperdiploidy	79 (14.5)	-

Risk group stratifications were determined according to the study protocols.

The 529 control patients (median years of age 12.5 (1.5–59)) of the same ethnicity and from the same geographical region as the patients were randomly selected from healthy blood donors and from minor outpatients from the Orthopaedic Department in the Budai Children's Hospital, and from the Urological Department of Heim Pal Pediatric Hospital, Budapest. None of the controls have had childhood ALL or any other types of cancers previously.

Written informed consent was obtained from the study participants or from the next of kin, caretakers, or guardians on the behalf of the minors/children participants involved in the study. All the associated documents have been stored. The study was conducted according to the principles expressed in the Declaration of Helsinki and the whole study including the informed consent procedure were approved by the Hungarian Scientific and Research Ethics Committee of the Medical Research Council (ETT TUKEB; Case No.:8-374/2009-1018EKU 914/PI/08.)

### Genes and polymorphisms

Based on the relevant scientific publication results (candidate gene association and GWA studies) and the dbSNP database of the National Center for Biotechnology Information (NCBI; www.ncbi.nlm.nih.gov. Accessed: 2013 Jun 20.) we selected and genotyped 67 single nucleotide polymorphisms in 15 key genes encoding the main enzymes directly (MTHFR, MTRR, MTHFD1) or indirectly (TPMT, GSTP1) involved in the folate cycle through which the DNA synthesis, the DNA methylation and repair mechanisms can be regulated (Table 2).

**Table 2:** List of polymorphisms investigated in this study.

Gene	SNP ID	Position^1^	Function^2^
**ABCB1**	rs10280101	chr7:86991521	intron
	rs1202179	chr7:87042215	intron
	rs2235013	chr7:87016562	intron
	rs9282564	chr7:87067376	missense,Asn>Asp
**DHFR**	rs11742668	chr5:79975660	intron
	rs1222809	chr5:79953273	unknown
	rs12517451	chr5:79955829	near-gene-3
	rs1478834	chr5:79985331	intron
	rs1650723	chr5:79957786	near-gene-3
	rs1677626	chr5:79985201	intron
**FPGS**	rs10106	chr9:129615896	untranslated-3
	rs1544105	chr9:129602546	unknown
	rs4451422	chr9:129616418	near-gene-3
**GGH**	rs10957267	chr8:64106805	intron
	rs11545078	chr8:64101318	missense, Thr>Ile
	rs3780127	chr8:64103693	intron
	rs719235	chr8:64114235	near-gene-5
**GSTP1**	rs1695	chr11:67109265	missense,Ile>Val
	rs749174	chr11:67109829	intron
	rs7941395	chr11:67103993	unknown
**MTHFD1**	rs1076991	chr14:63924794	near-gene-5
	rs1950902	chr14:63952133	missense,Lys>Arg
	rs2236225	chr14:63978598	missense, Arg>Gln
	rs745686	chr14:64000385	near-gene-5
**MTHFR**	rs13306561	chr1:11788391	intron
	rs1801131	chr1:11777063	missense,Glu>Ala
	rs1801133	chr1:11778965	missense,Ala>Val
**MTRR**	rs10380	chr5:7950191	missense,His>Tyr
	rs1532268	chr5:7931179	missense,Ser>Leu
	rs162036	chr5:7938959	missense,Lys>Arg
	rs1801394	chr5:7923973	missense,Ile>Met
	rs2966952	chr5:7921030	near-gene-5
	rs326120	chr5:7927847	intron
	rs3776455	chr5:7949511	intron
**MTR**	rs10925257	chr1:235112783	intron
	rs12759827	chr1:235038809	intron
	rs1805087	chr1:235115123	missense,Asp>Gly
	rs2853523	chr1:235128821	untranslated-3
	rs3768142	chr1:235095187	intron
	rs4659724	chr1:235040747	intron
**SHMT1**	rs1979277	chr17:18172821	missense,Leu>Phe
	rs643333	chr17:18207764	near-gene-5
	rs9909104	chr17:18188746	intron
**SLC19A1** 3	rs1051266	chr21:45782222	missense,His>Arg
	rs4819128	chr21:45774077	intron
	rs7499	chr21:45756756	untranslated-3
**SLC22A8**	rs2276299	chr11:62523007	coding-synon,Thr>Thr
	rs3809069	chr11:62540348	near-gene-5
	rs4149183	chr11:62522445	intron
**SLCO1B1** 4	rs10841769	chr12:21286286	unknown
	rs11045818	chr12:21221028	coding-synon,Ser>Ser
	rs11045819	chr12:21221080	missense,Pro>Thr
	rs11045823	chr12:21225012	intron
	rs17328763	chr12:21173837	near-gene-5
	rs4149056	chr12:21222816	missense,Val>Ala
	rs4363657	chr12:21259989	intron
**TPMT**	rs2518463	chr6:18251748	intron
	rs2842951	chr6:18243662	intron
	rs4449636	chr6:18255998	intron
**TYMS**	rs1004474	chr18:650383	intron
	rs2612100	chr18:662363	intron
	rs2853533	chr18:648064	intron
	rs2853741	chr18:647352	near-gene-5
	rs9967368	chr18:646020	near-gene-5

1 Position according to NCBI Genome Build 36.0;

2 Function according to the UCSC Genome Browser http://genome.ucsc.edu/(Accessed: 2013 Jun 20.);

3 RFC = SLC19A1;

4 SLCO1B1 = SLC21A6.

The criteria of the SNP selection were the minor allele frequency≥10% in Caucasian populations, their estimated functionality (missense, UTR, intron) or relevant results published previously in the scientific literature [8], [11], [12], [22]–[35].

Two of the selected SNPs were monomorphic and one of them did not meet the requirements of Hardy-Weinberg Equilibrium, and hence 64 single nucleotide polymorphisms were involved in the analyses.

### Genotyping

The genomic DNA of ALL patients was obtained from peripheral blood in a retrospective manner using QIAmp DNA Blood Maxi Kit (Qiagen, Hilden, Germany). The healthy control DNA was isolated with iPrep PureLink gDNA Blood Kit, iPrep Purification Instrument (Invitrogen, Life Technologies Co., Grand Island, USA).

Single nucleotide polymorphisms (SNPs) were genotyped by Sequenom iPLEX Gold MassARRAY technology at the McGill University and Génome Québec Innovation Centre, Montréal, Canada. Only SNPs with genotyping call rate over 90% were included in the analysis.

### Statistical analysis

#### Frequentist statistical analysis

Gender adjusted logistic regression model was applied to obtain odds ratios (ORs) and 95% confidence intervals (CIs) to estimate risks for each polymorphism to childhood ALL in the case-control data. Calculation of the genotype frequency and the allele positivity was performed by IBM SPSS Statistics software, version 21.0., and the allele frequency was analysed by Medcalc Version 12.4.0.

The statistical analyses were performed not only for the overall ALL population but also for the clinical subtypes (B-, T-cell and hyperdiplod (HD)-ALL).

The Hardy-Weinberg equilibrium analysis was carried out by χ2 goodness-of-fit test through an online application (http://ihg.gsf.de/cgi-bin/hw/hwa1.pl. Accessed: 2013 Jun 20. (see Table S2).

Multiple testing corrections were performed using the Benjamini-Hochberg false discovery rate (FDR) method with type I error rate of 5% (p≤1,21E-03) [36], [37]. Thus, „p” values<0.05 that could not reach the significance threshold (p≤1.21E-03) of the false discovery rate of 5%, were called nominally significant.

The statistical power was calculated by R, using an own implementation of the Genetic Power Calculator of Purcell and Sham [38]. The power calculations were adjusted using the same hypothesis correction method as described above (α = 1,21E-03). We calculated the power for the estimated allele frequencies and ORs for all SNPs (see Figure S3). We also performed a standard power calculation for different genetic models in our sample groups for SNPs with typical minor allele frequencies (see Figure S4). For example, in case of B-cell ALL, a SNP with minor allele frequency of 0.4 and with a recessive genotype relative risk of 2 would result in a power of 0.75 using a recessive genetic model.

Linkage disequilibrium and haplotypes were calculated with Haploview version 4.2. Odds ratios for haplotypes were obtained by MedCalc 12.4.0 software.

The survival analysis was carried out with IBM SPSS Statistics Software version 21. The survival rates were estimated with the Kaplan-Meier Method.

Associations and interactions were also investigated by the MDR method [15] using default settings.

#### Bayesian statistical methods

In our previous studies, the application of the BN-BMLA were explained in details [39]. Here, we briefly summarize the essence of the method.

The BN-BMLA method applies a Bayesian approach, which means that it computes the *a posteriori probability* of the strongly relevant variable sets with respect to a target variable (e.g. the variable describing the case/control status of the patients in a genetic association study) [17]. The strongly relevant variables have a direct influence on the target. In other words, these variables probabilistically shield the target from the effect of other variables. The *a posteriori probability* of the *strong relevance* (posterior) ranges from 0 to 1 and a posterior of 1 means that the target (e.g. immunophenotypes of ALL) definitely has a dependency relationship with a predictor (e.g. SNP), whereas 0 means there is no such relationship. S*trong relevance* has two types: *direct relevance* (e.g. a causal SNP) and *pure interaction* (e.g. a SNP with an epistatic effect).

Besides *strong relevance*, other forms of structural associations exist. In this context, we examined *statistical interaction* and *redundancy*. In a structural approach, statistical interaction means that SNPs tend to occur together more often in the model as strongly relevant variables than it is expected according to the assumption of their independent relevance (for details, see [17]). On the other hand, redundancy means that two SNPs tend to be interchangeable in strongly relevant variable sets.

The Bayesian structure based odds ratio is a recent extension to BN-BMLA which applies a hybrid approach towards effect size estimation by combining parametric relevance and strong relevance aspects [40]. A Bayesian odds ratio is related to a specific target, and it is conditioned on corresponding dependency models (i.e. graph structures representing the interactions of strongly relevant variables). The result is a posterior distribution over odds ratios, which provides a finer characterization of effect size and allows a more detailed analysis than a conventional confidence interval. Credible intervals (i.e. Bayesian analogue of confidence intervals) were computed based on the 95% HPD (high probability density) region of the Bayesian odds ratio.

## Results

### Frequentist statistical analysis

The influences of the 64 polymorphisms in the folate pathway on the risk of childhood acute lymphoid leukemia (ALL) were investigated.

Out of the 67 germline polymorphisms, 64 were statistically evaluated since two of them were monomorphic (rs12937300 *SHMT1*, rs1128503 *ABCB1*, not listed in Table 2), and one of them did not comply with the Hardy-Weinberg Equilibrium (HWE) (rs2032582 *ABCB1*, not listed in Table 2). Table S2 shows the results of the HWE and the association tests to ALL risk for all SNPs.

To examine the genotype frequencies in patient and control groups, we carried out gender adjusted logistic regression analyses on the full cohort and on various subgroups.

All the genotype and minor allele frequencies (MAF) for the investigated polymorphisms are reported in Table S3.

The frequentist statistical analysis revealed that 9 SNPs (rs2235013, rs12517451, rs1544105, rs1076991, rs12759827, rs9909104, rs2853533, rs3776455, rs1532268) in 8 genes (*ABCB1, DHFR, FPGS, MTHFD1, MTR, SHMT1, TYMS, MTRR*) reached the p<0.05 values (Table S4), but due to the multiple testing, statistical corrections were applied. In this way 2 SNPs of the *MTHFD1* and *MTRR* genes reached the FDR = 5% (p≤1.21E-03) significance threshold (see Table 3).

**Table 3:** Summary of the significant results of the frequentist-based statistical analyses.

Type of analysis		Overall		B-ALL		T-ALL		HD^a^-ALL	
		p^b^	OR (95%CI)	p^b^	OR (95%CI)	p^b^	OR (95%CI)	p^b^	OR (95%CI)
**rs1076991 (** ***MTHFD1*** **)**									
Genotype frequency	AA	Reference							
	AG	0.01	1.49(1.12–1.97)	0.01	1.52(1.12–2.08)	0.29	1.37(0.77–2.43)	0.77	1.08(0.63–1.85)
	GG	**1.94E**–**04**	**1.94(1.37**–**2.76)**	**3.52E**–**04**	**2.01(1.37**–**2.94)**	0.07	1.90(0.96–3.76)	0.66	1.17(0.59–2.33)
Allele positivity	AA vs AG/GG	**4.90E**–**04**	**1.61(1.23**–**2.1)**	**8.84E**–**04**	**1.65(1.23**–**2.22)**	0.14	1.51(0.88–2.59)	0.70	1.11(0.67–1.83)
	AA/AG vs GG	0.01	1.51(1.12–2.03)	0.01	1.53(1.11–2.12)	0.13	1.56(0.88–2.76)	0.72	1.12(0.67–2.04)
Allele frequency	A vs G	**1.30E**–**04**	**1.39(1.18**–**1.65)**	**2.10E**–**04**	**1.42(1.18**–**1.71)**	0.08	1.36(0.97–1.90)	0.65	1.08(0.77–1.51)
**rs3776455 (** ***MTRR*** **)**									
Genotype frequency	AA	Reference							
	AG	0.57	1.08(0.83–1.40)	0.79	1.04(0.78–1.38)	0.14	1.48(0.88–2.48)	0.43	1.22(0.74–2.02)
	GG	4.49E–03	0.57(0.38–0.84)	0.01	0.57(0.37–0.87)	0.26	0.61(0.26–1.45)	0.01	0.15(0.04–0.65)
Allele positivity	AA vs AG/GG	0.57	0.93(0.73–1.19)	0.46	0.90(0.69–1.18)	0.42	1.23(0.74–2.02)	0.73	0.92(0.56–1.50)
	AA/AG vs GG	**1.21E**–**03**	**0.55(0.38**–**0.79)**	0.01	0.56(0.37–0.83)	0.09	0.49(0.22–1.11)	0.01	0.14(0.03–0.57)
Allele frequency	A vs G	0.04	0.83(0.69–0.99)	0.04	0.82(0.67–0.99)	0.70	0.93(0.66–1.32)	0.05	0.69(0.48–1.01)

a HD: Hyperdiploid ALL;

b p-values in bold reached the p≤1.21E-03;

FDR(α) = 5.0% significant threshold.

The genotype distribution of the *MTHFD1* rs1076991 differed significantly between the overall ALL and control population (p = 1.94E-04; OR = 1.94 (1.37–2.76) for the GG genotype; power = 0.64). Subsequently, it was investigated whether this SNP was associated with the clinical characteristics of ALL. As can be seen in Table 3 the GG genotype increased the risk of B-cell ALL (p = 3.52E-04; OR = 2.00 (1.37–2.94); power = 0.59), but not of T-cell, or HD-ALL. Further evaluation of these results showed that the G allele positivity increased significantly the susceptibility to ALL both in allelic (50% vs. 41.8%; p = 1.30E-04; OR = 1.39 (1.18–1.65) and genotype levels (AA vs. AG/GG; p = 4.90E-04; OR = 1.61 (1.23–2.10)). Analyzing the ALL subgroups separately showed that this SNP increased the risk exclusively to the B-cell ALL.

The frequency of the *MTRR* rs3776455 GG genotype differed between the ALL and control population (p = 4.49E-03; OR = 0.57 (0.38–0.84); power = 0.51). Further analysis of the genotype distribution showed that the GG genotype was associated with a significantly reduced risk to ALL (AA/AG vs. GG; p = 1.21E-03; OR = 0.55 (0.38–0.79)).

### Haplotype analysis and Linkage Disequilibrium test

Haplotype analyses were carried out to study the influence of haplotype blocks of genes on ALL risk. Two haploblocks in the *MTHFD1* gene were found to influence the risk of ALL in our population. The ACTA haplotype (Table 4) had a slightly protective effect against ALL (p = 0.003; OR = 0.74 (0.61–0.90)). In contrast, people carrying the GCCA haploblock, had 1.38 fold greater risk (p = 0.009; OR = 1.38 (1.08–1.76)) to ALL (Table 4).

**Table 4:** Estimated haplotype frequencies in the *MTHFD1* gene in ALL and control population.

Haplotype	ALL	Control	p	OR (95% C.I.)
***MTHFD1*** **(rs1076991,rs1950902, rs2236225,rs745686)**				
ACTA	248 (23%)	302 (29%)	0.003	0.74 (0.61–0.90)
GCCA	181 (17%)	134 (13%)	0.009	1.38 (1.08–1.76)

Linkage disequilibrium coefficients (D', and r^2^ Table S5) were calculated for each of the significant SNPs in both genes (*MTHFD1* rs1076991, *MTRR* rs3776455) and found that the rs3776455 SNP in *MTRR* gene, was in strong linkage with other six SNPs (rs2966952, rs1801394, rs326120, rs1532268, rs162036, and rs10380) in the gene. It suggests that the rs3776455 SNP can determine the status of the other six SNPs, and thus can be regarded as a tagSNP.

### Effect of the investigated SNPs on the survival rates of the Hungarian ALL population

Earlier we have already reported the survival rate of this population in more details [41]. In summary, the overall survival rate was 85.5%, while the event-free survival rate was 81.0% in this pediatric ALL population.

The logrank test was used to estimate whether these SNPs influenced the overall or the event-free survival rates. An association between the overall survival rates and the rs9909104 SNP in the *SHMT1* gene was observed (Table 5). Overall survival of patients with TC (80.2%) was significantly lower than the survival of the patients with major TT (88.8%) genotype (p = 0.01).

**Table 5:** Influence of the rs9909104 (*SHMT1*) polymorphism on the overall and event-free survival time in ALL.

Case processing summary					Means*				
Coding	Total no.	Events	Censored	Percent	Estimate	Std.error	95% C.I.	Chi^2^	p
**Overall survival**									
Wild	303	34	269	88.8%	16.2	0.3	15.6–16.9	6.4	4.0E-02
Carrier	177	35	142	80.2%	14.6	0.5	13.6–15.5		
Mutant	31	5	26	83.9%	11.7	0.8	10.2–13.2		
Overall	511	74	437	85.5%	15.7	0.3	15.2–16.2		
**Event-free survival**									
Wild	303	49	254	83.8%	15.4	0.4	14.7–16.1	3.2	2.0E-01
Carrier	177	40	137	77.4%	14.0	0.5	13.0–15.1		
Mutant	31	6	25	80.6%	11.2	0.9	9.5–12.9		
Overall	511	95	416	81.4%	15.0	0.3	14.4–15.6		

* Log Rank Test (Mantel-Cox).

### Bayesian analysis of relevance and effect size

#### Strongly relevant SNPs and genes

We used the BN-BMLA method to infer the posterior probability of strong relevance of the genetic markers with respect to ALL susceptibility.

The SNPs and genes with the highest posterior probability of strong relevance according to the BN-BMLA method are shown in Table 6 (see Table S6 for all SNPs). Similarly to the frequentist analysis, the most relevant SNP in ALL susceptibility was the rs1076991 of the *MTHFD1* gene with a posteriori probability of strong relevance of 0.65. In B-cell lineage sample group, the probability of the strong relevance was 0.53, while in the T-cell lineage sample group, this probability was 0.13, and in the hyperdiploid sample group it was nearly zero. This means, that the evaluation with the BN-BMLA confirmed the results of the frequentist analysis, i.e. this SNP influenced the risk exclusively of the B-cell ALL. Furthermore, we validated these results with MDR. It selected rs1076991 of the *MTHFD1* as the best univariate model for explaining ALL in the B-cell sample group and in the whole sample group with overall accuracy values 0.5513 and 0.5486 respectively. In case of the hyperdiploid and T-cell sample group rs1076991 was ranked as less relevant.

**Table 6:** Probability of strong relevance of the most relevant variables in different sample groups according to the BN-BMLA.

Variables		ALL susceptibility	B-ALL susceptibility	T-ALL susceptibility	HD-ALL susceptibility
Gender		0.01	0.05	**0.71**	0.05
rs1076991	*MTHFD1*	**0.65**	**0.53**	0.13	0.00
rs1004474	*TYMS*	0.00	0.00	0.26	**0.66**
rs1532268	*MTRR*	0.00	0.03	0.21	**0.68**
rs3776455	*MTRR*	0.35	0.08	0.02	**0.76**
rs1222809	*DHFR*	0.00	0.01	0.12	0.43
rs11545078	*GGH*	0.03	0.06	0.16	0.33
rs3780127	*GGH*	0.03	0.06	0.16	0.33

**Figure 2:**
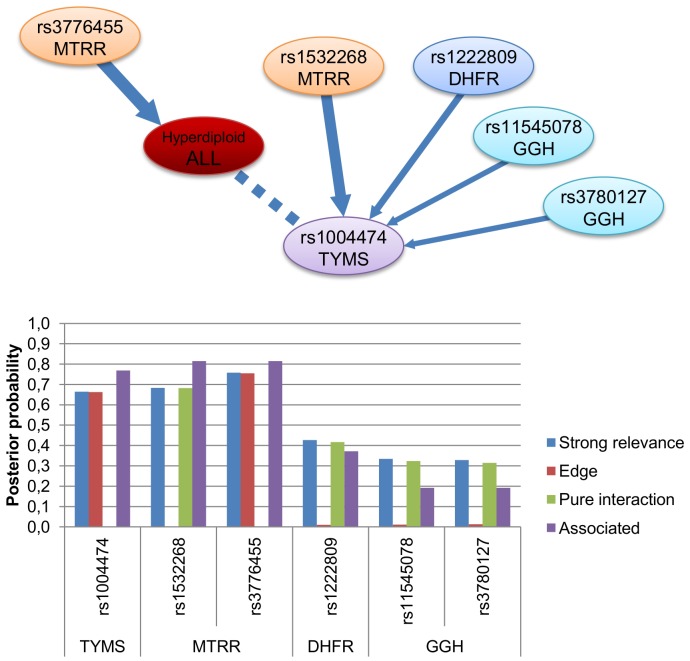
Illustration of different dependency relations between relevant SNPs and hyperdiploid ALL susceptibility. Top panel: An interactional map including hyperdiploid ALL susceptibility (red node) and strongly relevant SNPs with respect to it (other nodes). The width of the edges is proportional to their a posteriori probability. The directed edges represent only probabilistic relationships between the variables which are not necessary causal. Bottom panel: The posterior probability of strong relevance (blue columns), edge (direct strong relevance, red columns), pure interaction (green columns) and association (purple columns) of the variables to hyperdiploid ALL susceptibility according to the BN-BMLA method.

In the hyperdiploid sample group, the relevant SNPs were rs3776455 and rs1532268 in the *MTRR* gene (probability of strong relevance to HD-ALL was 0.76 and 0.68, respectively), and rs1004474 in *TYMS* (0.66). The SNP rs3776455 in the *MTRR* gene was selected as the best univariate model by MDR with an overall accuracy score of 0.5724. MDR found the other two SNPs relevant only in interaction as described later. A few other SNPs also showed low probabilities of relevance in ALL susceptibility (see Figure 2).

### Detailed characterization of association relations

In recent publications we described the characteristics and the calculation of the different structural features of the different dependency types between the variables calculated by BN-BMLA [39], [41]. The features are also summarized in Table S7 of this paper and the results of the present study are shown in Table S8.

According to the BN-BMLA the probability that rs2236225 and rs745686 in the *MTHFD1* gene were associated to ALL susceptibility was 0.71, but their probabilities of strong relevance to ALL were nearly zero while the value of their transitive relevance was 0.61, meaning that this association is probably mediated by the direct relevance of rs1076991 in the *MTHFD1* gene. The situation is the same in the case of *MTRR* gene, where all measured SNPs (rs2966952, rs1801394, rs326120, rs1532268, rs162036, rs3776455 and rs10380) had around 0.65 probability of being associated to ALL, but only rs3776455 had a moderately high probability of strong relevance, meaning that the associations were mainly affected through this SNP.

In the B-cell lineage sample group, three SNPs (rs1076991, rs2236225 and rs745686) in *MTHFD1* gene showed association to ALL with probabilities between 0.57–0.67, mediated by rs1076991. Both in the overall ALL and the B-cell lineage sample groups, all investigated SNPs in *SLCO1B1* (*SLC21A6*) were associated to ALL with probabilities between 0.51–0.55, while the probabilities of the direct relevance of these SNPs were below 0.25. This means that in the case of this gene the exact structural characteristics of the relations between these SNPs and the B-cell ALL risk could not be determined but these results indicated a possible role of this gene in the disease.

In the case of the T-cell lineage sample group the SNPs in *TYMS* gene showed a relatively high probability of association (between 0.64–0.74), but only rs2853533 and rs1004474 SNPs had moderately high value of strong relevance, meaning that the associations were mediated through these SNPs.

Similarly to the above detailed explanations, in the hyperdiploid sample group, the SNPs in the *TYMS* gene were related to HD-ALL with probabilities of 0.72–0.77 mediated through the effect of rs1004474; and SNPs in *MTRR* (rs2966952, rs1801394, rs326120, rs162036 and rs10380) were associated (0.79–0.82) via rs1532268 and rs3776455 (Table S8).

### Structural interactions, statistical interactions and redundancies

We examined different types of interactions, namely structural interactions, statistical interactions and redundancies between the strongly relevant variables. In cases of the overall, B- and T-cell lineage sample groups, the analyses revealed no pure interactional effect (see explanations in Table S7), i.e. the probability of pure interaction was below ∼0.2 in case of all SNPs in these sample groups.

However, in the hyperdiploid sample group some SNPs had moderately high probabilities for pure interaction (e.g. 0.68 in case of rs1532268 in *MTRR*). Figure 2 shows the complex structural interactional map and the probabilities of the different structural association types of the relevant SNPs with respect to hyperdiploid ALL susceptibility. The SNPs rs1532268 in *MTRR*, rs1222809 in *DHFR*, rs11545078 and rs3780127 in *GGH* were in pure interaction with hyperdiploid ALL through rs1004474 in *TYMS*. Note, that directed edges represent only probabilistic relationships between the variables which are not necessary causal.

In accordance with this, the interaction graph stated that for example rs1532268 in the *MTRR* gene was conditionally independent of hyperdiploid ALL, but knowing the genotype of rs1004474 in *TYMS* rendered it dependent. Therefore, the values of rs1004474 and its “parent” SNPs' in the graph had a joint effect on the value of hyperdiploid ALL without a marginal effect of the “parents”.

We also analyzed the interaction of these SNPs with MDR. The interaction of SNPs rs1532268 in *MTRR* and rs1004474 in *TYMS* was among the top 10 models containing two variables with an overall accuracy score of 0.612 (the best model had 0.6239). MDR showed that the SNPs rs1222809 in *DHFR*, rs11545078 and rs3780127 in *GGH* were relevant only in models containing three variables. These models consisted of the previously discussed interacting SNPs rs1532268 in *MTRR* and rs1004474 in *TYMS* and one of the following additional terms: rs1222809 in *DHFR*, rs11545078 and rs3780127 in *GGH* with accuracy scores 0.6513, 0.6566 and 0.6566, respectively (whereas the best model with three elements had 0.6842).

**Figure 3:**
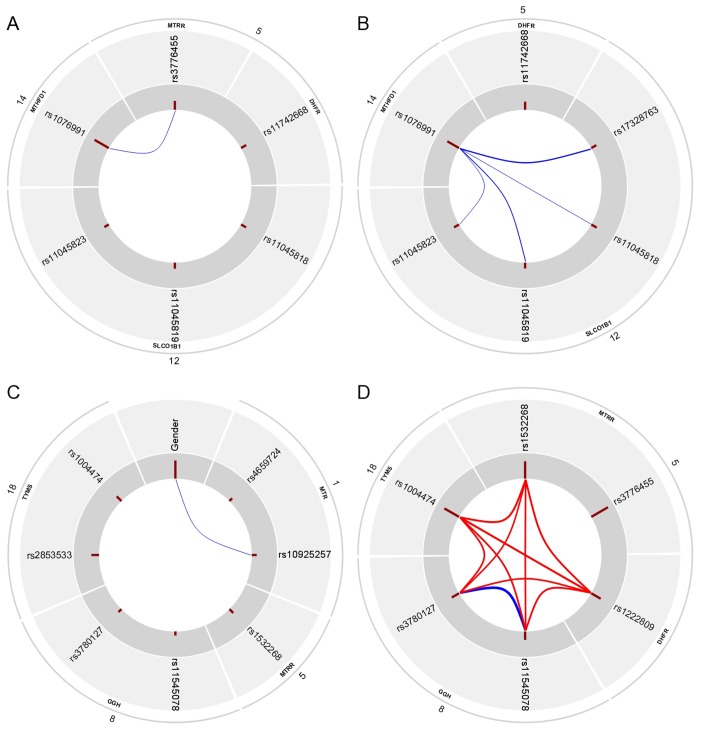
Redundancies and interactions according to the BN-BMLA method. The figure shows the magnitude of redundancies (blue curved lines) and interactions (red curved lines) between the variables in the overall (i.e. ALL susceptibility, A panel), in the B-cell lineage (B panel), in the T-cell lineage (C panel) and in the hyperdiploid patient group (D panel) according to the BN-BMLA method. The width of the curved lines is proportional to the strength of the effect. The a posteriori probability of the strong relevance of the variables is proportional to the length of the dark red columns next to the variable in the inner gray colored ring. The corresponding genes and chromosomes of the SNPs are shown on the outer ring.

We computed the statistical interactions and redundancies between all variables in case of all sample groups. Figure 3 shows the results in a graphical form. A statistical interaction means that two (or more) SNPs tend to occur together more often in the hypotheses of the strongly relevant variable sets than it is expected (i.e. the joint strong relevance of the SNPs is more probable than it is expected). On the other hand, redundancy means that two SNPs are somewhat interchangeable in the hypotheses of the strongly relevant variable sets (i.e. the probability of the joint strong relevance of the SNPs is lower than it is expected).

In the whole patient group, rs1076991 in *MTHFD1* and rs3776455 in *MTRR* showed a weak redundancy (Redundancy ratio (RR) = 0.12, see Figure 3A). In the B-cell lineage sample group, rs1076991 in *MTHFD1* showed a weak redundancy with several SNPs in *SLCO1B1*, namely rs17328763 (RR = 0.24), rs11045819 (RR = 0.16), rs11045818 (RR = 0.13) and rs11045823 (RR = 0.11). See Figure 3B for a graphical representation.

In case of the T cell lineage sample group, the gender showed a weak redundancy with rs10925257 in *MTR* (RR = 0.11, see Figure 3C).

SNPs in the structurally interacting component described above also had a strong statistical interaction in the hypotheses of the strongly relevant variable sets in hyperdiploid ALL (see Figure 3D). This component involved rs1004474 in *TYMS*, rs1532268 in *MTRR*, rs1222809 in *DHFR*, and one of the two SNPs rs11545078 and rs3780127 in *GGH* (interaction ratio (IR) = 1.14, including rs11545078; and IR = 1.12, including rs3780127). In line with this, the two SNPs in *GGH* showed a strong redundancy (RR = 0.85). It must be noted, however, that this dataset was relatively small, thus the exact characterization of these effects needs further validation.

### Bayesian effect size analysis

Bayesian odds ratios and corresponding credible intervals were investigated for relevant SNPs in order to characterize their effect on ALL.

In case of *MTHFD1* rs1076991 the results confirmed its significant effect on the susceptibility to ALL. Both the AG and the GG genotype increased the risk of ALL with credible intervals of (1.44–1.52) and (1.88–2.01), respectively (see Table 7). The related posterior distribution curves were highly peaked (see Figure S1) meaning that all possible dependency models of factors supported that *MTHFD1* rs1076991 had a strong effect on ALL based on the overall population. This effect could also be observed in case of B-cell ALL patients. The credible intervals were similarly narrow (AG: 1.49–1.55, GG: 1.96–2.11), and the posterior distribution curves of the Bayesian odds ratios were similarly peaked. For T-cell ALL patients the *MTHFD1* rs1076991 had a similar effect although not as significant as in the previous cases. The posterior distribution curve was flat indicating that different dependency models entailed somewhat different odds ratios (see Figure S1). Consequently, credible intervals were relatively wider (AG: 1.38–1.68, GG: 1.79–2.08). In contrast, the effect of *MTHFD1* rs1076991 was negligible in the HD-ALL subgroup.

**Table 7:** Credible intervals (95%) corresponding to the Bayesian odds ratios of MTHFD1 rs1076991 and MTRR rs3776455.

	ALL			B-ALL			T-ALL		
	95% CR-L	95% CR-U	Prob. weight	95% CR-L	95% CR-U	Prob. weight	95% CR-L	95% CR-U	Prob. weight
**Bayesian odds ratios of rs1076991 (** ***MTHFD1*** **)**									
**AG vs AA**	1.44	1.53	100%	1.48	1.56	100%	1.38	1.68	100%
**GG vs AA**	1.88	2.01	100%	1.96	2.11	100%	1.79	2.08	100%
**Bayesian odds ratios of rs3776455 (** ***MTRR*** **)**									
**AG vs AA**	0.62	0.71	7%	0.99	1.04	75%	1.19	1.27	100%
	0.96	1.13	75%	1.33	1.39	25%			
	1.22	1.3	18%						
**GG vs AA**	0.34	0.6	84%	0.53	0.54	75%	0.09	0.2	100%
	0.73	0.99	7%	1.3	1.56	25%			
	1.25	1.39	9%						

CR: credible interval, a Bayesian analogue of the confidence interval, 95% CR-U and CR-L: upper and lower thresholds of the 95% credible interval.

The other SNP that was revealed to have a significant effect on the susceptibility to ALL was *MTRR* rs3776455. Based on the whole sample group the posterior distribution of its Bayesian odds ratio had multiple local maxima, resulting in disjoint credible intervals both for the AG (0.62–0.71, 0.96–1.13, 1.22–1.3) and the GG (0.34–0.6, 0.73–0.99, 1.25–1.39) genotypes (see Figure S1 and Table 7). This means that possible dependency models could be divided into three groups each supporting different odds ratios. In case of the AG genotype the majority of the models (75%) supported the neutral odds ratio with a credible interval of (0.96–1.13) which signified a non-relevant effect with relatively high probability. In case of the GG genotype however, the interval of (0.34–0.6) had the majority support of dependency models (84%), which confirmed the protective effect of the GG genotype with a high probability. On the other hand, the credible interval showing increased risk (1.25–1.39) also had some support (9%). This phenomenon might explain earlier results indicating the GG genotype as risk factor. Although the current study population indicates a protective effect, it is possible that the risk increasing effect could be observed under different circumstances.

A similar structural uncertainty could be observed in the case of B-cell ALL patients, although the posterior distribution of Bayesian odds ratios was only bimodal. The effect of AG genotype of *MTRR* rs3776465 was neutral (0.99–1.04) with moderately high probability (0.75), whereas the GG genotype had a protective effect (0.53–0.54) with a similar probability (0.75). In contrast, in the HD-ALL subgroup the related Bayesian odds ratios of *MTRR* rs3776465 indicated strong effects with narrow credible intervals. The AG genotype increased the risk of HD-ALL (1.2–1.27), while the GG genotype had a remarkable protective effect (0.09–0.2).

The interaction between *TYMS* rs1004474 and *MTRR* rs1532268 shown in Figure 2 were also further investigated in this respect. On one hand, the *TYMS* rs1004474 had a negligible effect on the risk of HD-ALL both in case of GA and GG genotypes (with respect to AA) with credible intervals of (0.75–0.97) and (0.94–1.10) respectively (Figure S2). On the other hand, both the GA and AA genotypes of *MTRR* rs1532268 led to a slightly increased risk to HD-ALL (1.28–1.40 and 0.97–1.72 with respect to GG), however the credible interval of the latter was exceedingly wide (Figure S2). The joint Bayesian odds ratio of *TYMS* rs1004474 and *MTRR* rs1532268 was computed by parametric averaging in a dominant model (Table S9). Remarkably, the combination of rs1004474 (AA) - rs1532268 (GG) (i.e. the common homozygous case for both SNPs) had the highest conditional probability of risk of HD-ALL, namely 0.23, which exceeded the individual effect of risk allele A for *MTRR* rs1532268 with a conditional probability of risk of 0.14. Note, that whereas the univariate analysis indicated an increased risk for the carriers of the *MTRR* rs1532268 A allele, the multivariate analysis revealed a more complex pattern of interaction: the *MTRR* rs1532268 A allele became protective in carriers of the AA genotype of the *TYMS* rs1004474 with a joint OR of 0.32 (Table S9).

## Discussion

In this study we investigated the associations of 67 SNPs in 15 genes in the folate metabolic pathway with the development of childhood ALL and whether they influenced the survival rates of the patients. Beside the traditional statistical methods, we also evaluated our results with the BN-BMLA method.

Altogether 9 SNPs (rs2235013, rs12517451, rs1544105, rs1076991, rs12759827, rs9909104, rs2853533, rs3776455, rs1532268) in 8 genes (*ABCB1, DHFR, FPGS, MTHFD1, MTR, SHMT1, TYMS, MTRR*) showed nominally significant association with the risk to ALL.

After the correction for multiple testing 2 SNPs of the *MTHFD1* and *MTRR* genes reached the significance threshold and a SNP in the *SHMT1* gene influenced the survival rate of the patients. We also demonstrated the additional information provided by the BN-BMLA method in relation to the traditional frequentist evaluation methods.

The two most frequently investigated non-synonym SNPs in the *MTHFR* gene, the rs1801133 (C677T, Ala222Val) and the rs1801131 (A1298C, Glu429Ala) did not show any association with the susceptibility to ALL or with the survival rates of the patients in our population. Although there are several reports showing associations between these polymorphisms and ALL risk [8], [42], [43], or survival [23], our results correspond to the results of a meta-analysis where the pooled odds ratio for these two variants did not show association with ALL risk [44], and also to some recent and relatively large studies, where neither SNPs showed association [26], [27]. In these papers it is also discussed in details what the explanations for these inconsistent findings can be.

MTHFD1 is one of the major components of the folate pathway. It is a trifunctional enzyme which catalyzes the conversion of tetrahydrofolate. The enzyme and its derivatives are important cofactors for de novo purine, pyrimidine and methionine synthesis [6], [8], [45]. In our study we found an association between rs1076991 polymorphism in the *MTHFD1* gene and B-cell ALL risk. The G allele significantly increased the risk to the disease especially in homozygous form. The rs1076991 SNP is located in the promoter region of the gene. Using a luciferase reporter gene assay, Carroll et al. found a significant effect of this polymorphism on the *MTHFD1* promoter activity. The construct with A allele had only 38% of the activity than that of with the G allele (T and C alleles in the original paper [28], but they are A/G according to the official databases, like dbSNP, and we use this nomenclature). Although in this paper a bioinformatic analysis did not reveal an alteration of any consensus transcription factor binding site that would explain the observed difference in activity between the two genotypes, this result means that the G allele could be associated with a higher *MTHFD1* gene expression level *in vivo*. Presently, it can not be explained, how an elevated MTHFD1 enzyme level can be associated with an increased risk to B-cell ALL. A plausible explanation can be, that elevated level of MTHFD1 can lead to a decrease availability of its substrate, 5,10-methylenetetrahydrofolate, resulting in lower rate of synthesis of deoxythymidine monophosphate from deoxyuridine monophosphate and therefore increasing the risk of uracil incorporation and chromosomal damage.

The non-synonymous rs2236225 (G1958A; arg653gln) polymorphism of the *MTHFD1* gene has been widely studied in different traits including ALL [8], [12], [29], [30]. In the present study we did not find any associations, which involved this variant, except two haplotypes based on 4 SNPs in the *MTHFD1* gene. But, according to the structures of the haplotypes, it is very probable, that the main signal responsible for this association came from the rs1076991 SNP, rather than from the rs2236225, because the haplotype with the rs1076991 G allele was associated with increased, while with the A allele with decreased risk to ALL.

The other significant association of this study is the decreased B-cell ALL risk associated with the GG genotype of the rs3776455 SNP in the *MTRR* gene. The gene codes for the methionine synthase reductase enzyme which plays an important role in the folate pathway. The methylation of homocysteine to methionine is catalyzed by MTR (methionine synthase) using vitamin B12 as a cofactor, in which the MTR may become inactivated due to the oxidation of vitamin B12 cofactor. MTRR could catalyze reductive methylation of vitamin B12 by using SAM as a methyl donor, leading to the activation of MTR.

Presently, there is no information about the possible function of the SNP. It does not change consensus sequence, and it has not earlier been identified as a phenotype influencing variant. The most studied SNP in the *MTRR* gene is the rs1801394, which causes a non-synonym isoleucine-to-methionine change at position 22 (A66G). The variant enzyme has a lower affinity for MTR [31] and some studies have reported elevated homocysteine levels for carriers of the homozygote wild-type genotype (AA) compared to other genotypes, while others have not [46]–[48]. Some studies found an association between A66G genotype and decreased risk to ALL, but a meta-analysis provided no evidence for an association [11]. In our study we have not revealed an association between rs1801394 and ALL risk, although it showed some linkage to the associated rs3776455 SNP. Similarly, in our population the rs3776455 SNP was in linkage to other SNPs, like rs162036, rs10380, which have earlier been found to be associated with 3-fold elevated risk for spina bifida [49]. In our study we have found association only between the rs3776455 and reduced risk to B-cell and HD-ALL. Further studies are needed to reveal how this SNP or variants in linkage can influence the risk to the disease.

The rs9909104 SNP in the *SHMT1* gene was weakly associated with a reduced risk to ALL and the minor allele in homozygous form with a reduced survival rate. Methotrexate (MTX) is an important part of the ALL chemotherapy. It is an antifolate drug and polymorphisms in the folate pathway probably influence the response to and toxicity of the MTX therapy. Our result is in line with those, which showed that variants in the folate pathway associated with decreased disease risk are often associated with poorer response to MTX therapy [33], [34], [50]. SHMT1 has the same substrate as MTHFD1 and MTHFR (5,10-methylenetetrahydrofolate), thus they are in competition for this substrate. The most frequently studied SNP in the *SHMT1* gene is the rs1979277 (1420G/A), which earlier has been found to be associated with decreased MTX sensitivity in pediatric ALL [35]. We also genotyped this SNP, but found no such connection. This SNP is in strong linkage with the associated rs9909104 (D' = 1), however, because the rs1979277 A allele is more frequent, thus the r^2^ is only 0.13, it means that the linkage is partly one-sided, i.e. the rs9909104 C allele always occurs together with the rs1979277 A allele, but in the other way it is not true. That can be the explanation how it is possible, that the rs9909104 SNP is associated with a reduced survival rate in our population, and possible with decreased MTX response, but the rs1979277 SNP did not. Because in the study of de Jonge et al [35], the rs9909104 SNP was not genotyped, it could also be hypothesized, that the signal in that study came also from the non-genotyped rs9909104 SNP linked with the rs1979277. It must be added, however, that rs1979277 SNP is a non-synonym variation (L474F) in the coding region of the gene, while until now no function has been associated with the rs9909104 SNP.

Next to the frequentist-based analysis, we also evaluated our results with the BN-BMLA method. We showed in our previous papers [39], [41], as well as in the present study, that BN-BMLA is capable of identifying not only those variants, which are directly associated with the target value, but also variants whose effects are mediated, or are only in interactions. In addition, because BN-BMLA uses a different approach than the traditional methods, and the Bayesian statistics offer an automated and normative solution for the multiple hypothesis testing problems [16], [17], the method can also be used as an alternative method for confirmation of the results. Earlier, we have tested the BN-BMLA method in a case-control setup using artificial datasets for identifying interactions and conditional relevance [51]. The BN-BMLA was proven to be superior over other multivariate methods using conditional models designed to detect associations between genotypic variables and the target variable.

In the present study BN-BMLA confirmed the role of the rs1076991 SNP in the *MTHFD1* gene, since it showed strong relevance to B-cell ALL and that of the rs3776455 in the *MTRR* which showed strong relevance to HD-ALL and association to ALL. The two SNPs also showed a slight redundancy, implying that they have similar, although independent effect.

The main strength of the BN-BMLA is that it can suggest multivariate structural features of the different dependency types between the variables. In this dataset such structural features could only be revealed in HD-ALL. It is contrary to the expectations, since one would have thought that polymorphisms in genes in an important metabolic pathway, which has been shown to have a role in the pathogenesis of ALL, must be in interactions. A possible explanation can be that the effect sizes of the particular interactions are too low and larger populations would be needed for their detection. HD-ALL might have a more homogenous genetic background, than B-cell or T-cell ALL, and thus detection of it required smaller population. Similar findings have been experienced in our previous study [41].

A particular feature of the gene-gene interactions was also revealed by the BN-BMLA. Certain genotypes of the *MTRR* rs1532268 SNP were associated with a slightly increased risk to HD-ALL, while they turned to be protective when occurred together with a given allele of a gene (*TYMS*) in the same metabolic pathway. Logically, this can especially happen, when a pathway, like the folate, is responsible for a sensitive balance of several important metabolites. Variations in this pathway can lead to both accumulation and depletion of these metabolites leading to different consequences. This result also suggests a possible reason for the inconclusive results of the different studies investigating only the effect of single SNPs, and shows the importance of the systems biologic approach.

Similarly to the previous studies, the BN-BMLA named further genes like *TYMS*, *DHFR*, *GGH* and SNPs, and also provided possible explanations, what their roles could be in the gene-gene interactions. The results were also confirmed by MDR, a statistical method frequently used for detecting multiple interactions [14]. Naturally, however, all of these findings must be verified in *in vitro* studies, although it will not be an easy task.

The heterogeneous results in the different subtypes of the ALL shown in Figure 3, also confirm the notion that ALL is not one disease, but rather several diseases with different pathogeneses, but with similar symptoms, and therapeutic possibilities. If we can reveal the differences in the pathogeneses, there can be a chance for more personalized and thus more effective therapies.

This study has several strengths and limitations. First, although our sample set contains similar rate of relapsed patients to what was observed in the whole population [33]. The rate of died patients, however, is lower in our study population. Patients who died during the chemotherapy due to therapy resistant progressive disease or due to infections or toxicities of therapy are underrepresented in our sample.

It is well known that association studies suffer from a possibility of false positive results. To reduce this possibility we applied multiple test correction and also the BN-BMLA method, which offers an automated and normative solution for the multiple hypothesis testing problems [16]. But, as was discussed earlier, because of the possible low effect sizes of the SNPs a larger population would have been required for detecting genetic interactions. To overcome this, meta-analyses or an international leukemia consortium can be a solution. Our group is also a member of The International Childhood Acute Lymphoblastic Leukaemia Genetics Consortium [52], and participated in similar studies [53]. But meta-analyses and studies based on international consortia suffer from problems caused by populations with heterogeneous genetic background and significantly different environmental conditions. It was shown that even a given genetic background could respond oppositely in different environments [54], [55]. The number of patients in this study is one of the largest ones relative to the population of the country (Hungary) compared to other studies [35]. One of the main strength of this study is the relatively large population with homogeneous ethnicity and environment. It can be one of the main reasons, that contrary to most studies where after multiple testing correction no significant associations could have been found, here we could present significant results even after the correction [26], [34], [35], [56]. It must be noted, however, that in this paper we discussed mainly those results, which remained significant after the multiple testing correction, or had>0.5 a posteriori probability in the BN-BMLA. But in this study we also detected a number of other nominally significant associations, which most other studies regarded as real associations, and discussed accordingly. Thus, our other results presented in supplementary tables can also be involved, and analyzed in subsequent meta-analyses. Furthermore, the power analysis in our study indicated the potential advantage of more samples to confirm the status of the non-significantly associated SNPs, particularly in case of non-replicating SNPs reported in other studies (e.g. rs1801133 - *MTHFR*, rs1801131 - *MTHFR*, rs2236225 – *MTHFD1*, rs1801394 - *MTRR*, rs1979277 – *SHMT1*).

**In conclusion**, in our study population the rs1076991 SNP in the *MTHFD1* gene and the rs3776455 in the *MTRR* gene significantly influenced the risk of ALL. The heterozygous form of the rs9909104 SNP in the *SHMT1* gene was associated with a lower survival rate. Next to these results several other SNPs in different genes in the folate metabolic pathway showed nominally significant associations with altered risk of ALL. The results were also evaluated by the BN-BMLA method, which confirmed the main findings and detected structural features of the different dependency types between the SNPs in the hyperdiploid subtype of ALL. These results give further evidence that polymorphisms in the folate pathway can influence the ALL risk and the effectiveness of the therapy. We also showed several advantageous features of the BN-BMLA method, and we demonstrated that in gene association studies it might be useful supplementary to traditional frequentist statistical methods. The tool is available at a public website [57].

## Supporting Information

Figure S1 **Posterior distribution curves for Bayesian odds ratios of rs1076991 (** ***MTHFD1*** **) and rs3776455 (** ***MTRR*** **) with respect to ALL and its subtypes.** Each curve depicts the outline of a histogram of possible odds ratio values within the 95% credible interval (Bayesian analogue of the 95% confidence interval) corresponding to genotype AG or GG (given AA as a reference). Bayesian odds ratio values are shown on the horizontal axis, whereas related probability values are displayed on the vertical axis.
(TIF)

Figure S2 **Individual effect of rs1004474 (** ***TYMS*** **) and rs1532268 (** ***MTRR*** **).**
(TIF)

Figure S3 **The power of the chi-square test of association for all SNPs in different sample groups.**
(PDF)

Figure S4 **Power calculations for varying effect sizes based on typical minor allele frequencies in different sample groups in the study.** The power of the chi-square test of association is calculated for different genetic models (rows) in different sample groups representing different sample sizes (columns) for SNPs with typical minor allele frequencies. For example, in case of B-cell ALL, a SNP with minor allele frequency of 0.4 and with a recessive genotype relative risk of 2 would result in a power of 0.75 using a recessive genetic model. The calculated powers are adjusted using the same multiple hypothesis testing correction as described in Methods.
(PDF)

Table S1 **Functions and names of the key enzymes and transporter molecules involved in the folate metabolism.**
(DOC)

Table S2 **Hardy–Weinberg equilibrium test for each of the studied polymorphisms.**
(DOC)

Table S3 **Genotype and minor allele frequencies (MAF) in patient (ALL) and control groups.**
(DOC)

Table S4 **Summary of the nominally significant results according to the frequentist analysis.**
(DOC)

Table S5 **Linkage disequilibrium coefficients (D' and r^2^) of the** ***MTHFD1*** **and** ***MTRR*** **gene polymorphisms.**
(DOC)

Table S6 **Probability of strong relevance to ALL susceptibility in different subgroups according to the BN-BMLA.**
(XLS)

Table S7 **Structural features of different dependence types between variables.**
(DOC)

Table S8 **A posteriori probabilities of the different dependence types in ALL susceptibility.**
(XLS)

Table S9 **Joint effect of TYMS rs1004474 and MTRR rs1532268 according to the Bayesian effect size analysis.**
(DOC)
