# Supplementary figures and images for: Roles of Genetic Polymorphisms in the Folate Pathway in Childhood Acute Lymphoblastic Leukemia Evaluated by Bayesian Relevance and Effect Size Analysis
Source: PLoS One. 2013 Aug 5;8(8):e69843. doi: 10.1371/journal.pone.0069843 (PMC3734218; doi:10.1371/journal.pone.0069843)

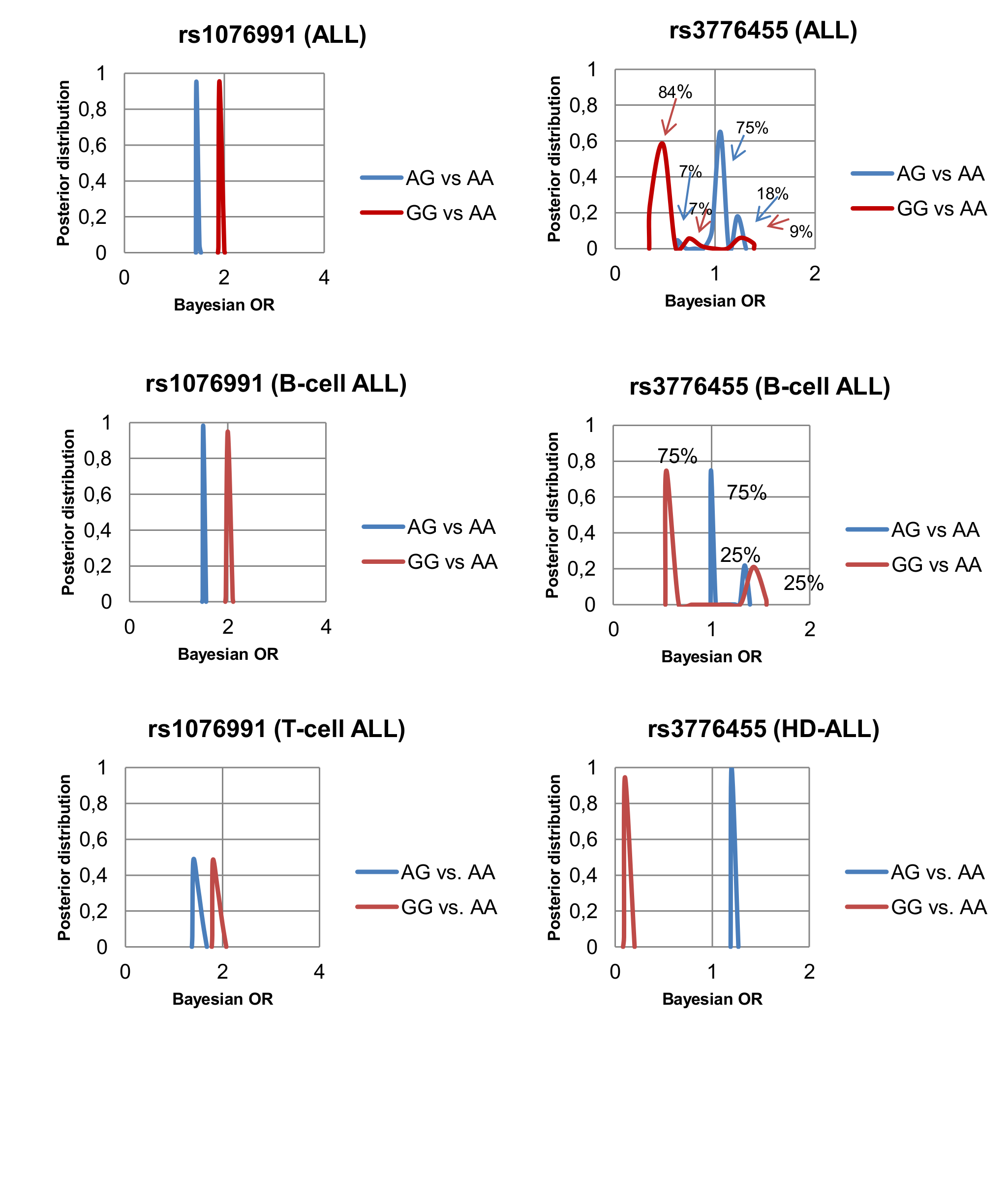

Supplement: Figure S1 — Posterior distribution curves for Bayesian odds ratios of rs1076991 ( MTHFD1 ) and rs3776455 ( MTRR ) with respect to ALL and its subtypes. Each curve depicts the outline of a histogram of possible odds ratio values within the 95% credible interval (Bayesian analogue of the 95% confidence interval) corresponding to genotype AG or GG (given AA as a reference). Bayesian odds ratio values are shown on the horizontal axis, whereas related probability values are displayed on the vertical axis. (TIF) [file pone.0069843.s001.tif]

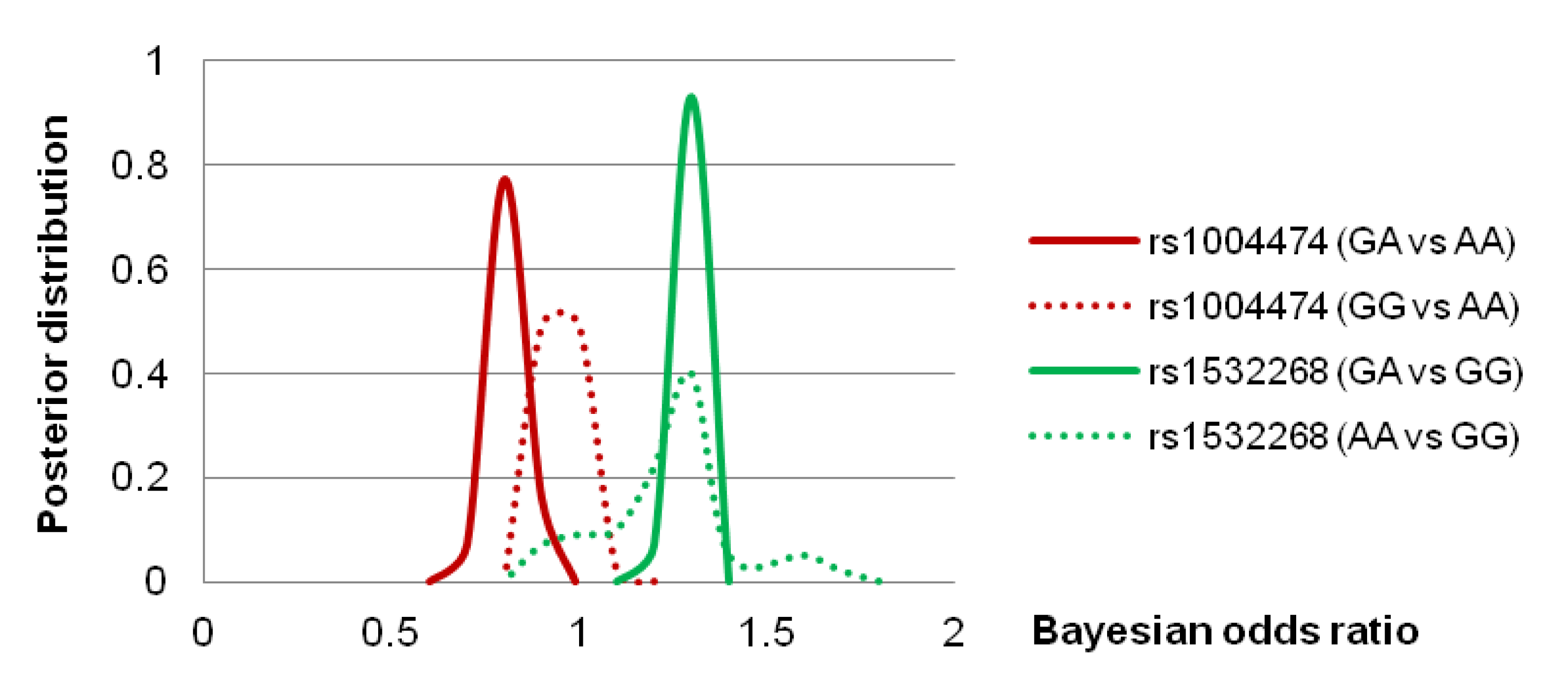

Supplement: Figure S2 — Individual effect of rs1004474 ( TYMS ) and rs1532268 ( MTRR ). (TIF) [file pone.0069843.s002.tif]

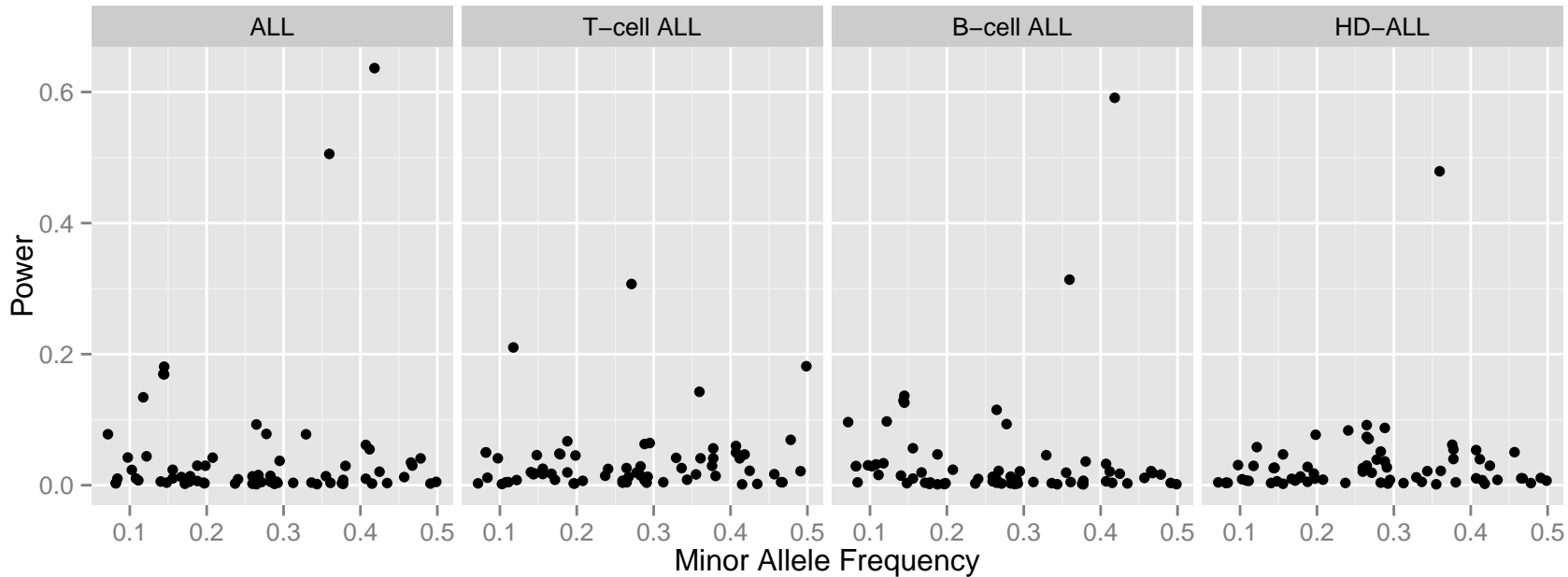

Supplement: Figure S3 — The power of the chi-square test of association for all SNPs in different sample groups. (PDF) [file pone.0069843.s003.pdf]

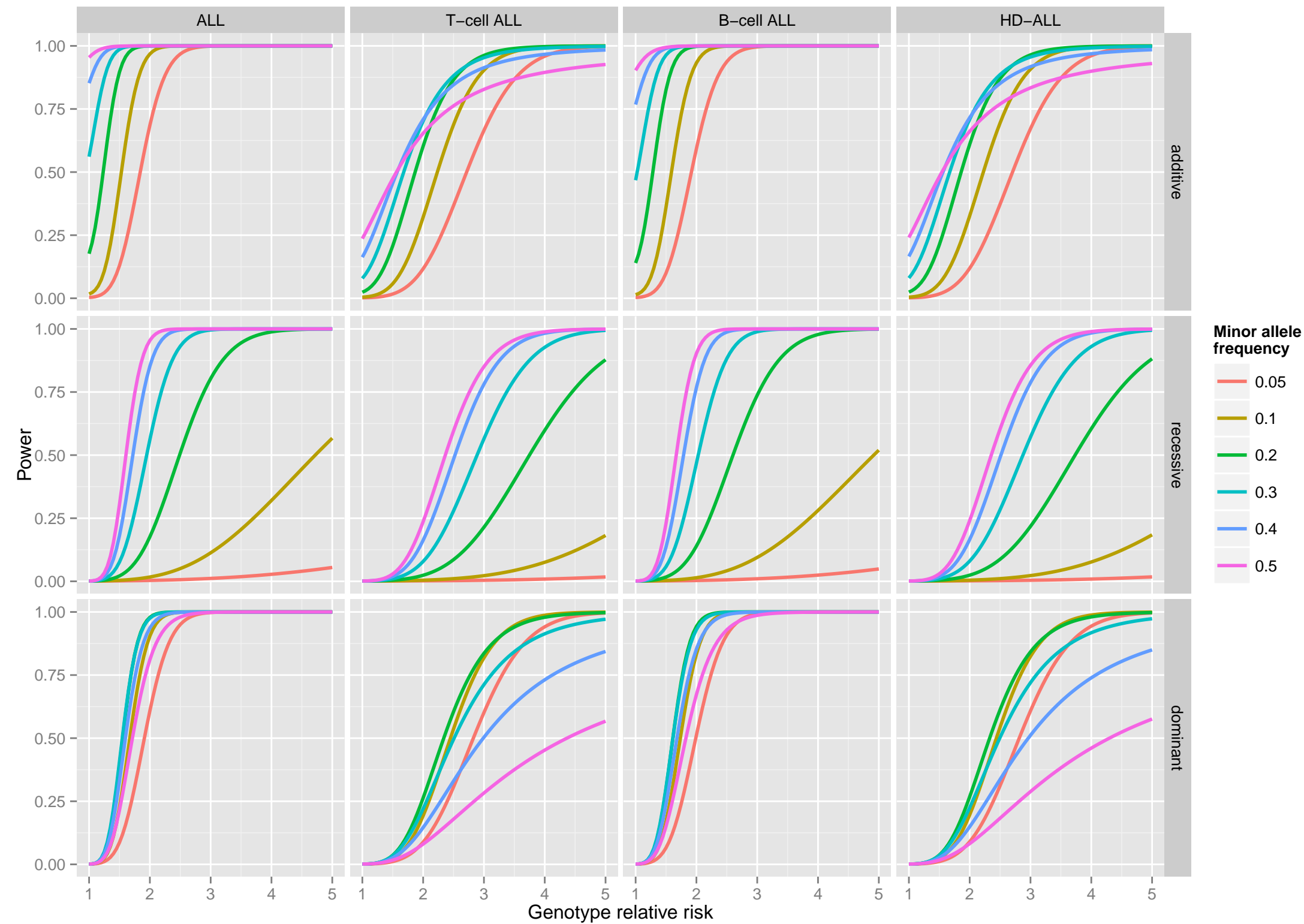

Supplement: Figure S4 — Power calculations for varying effect sizes based on typical minor allele frequencies in different sample groups in the study. The power of the chi-square test of association is calculated for different genetic models (rows) in different sample groups representing different sample sizes (columns) for SNPs with typical minor allele frequencies. For example, in case of B-cell ALL, a SNP with minor allele frequency of 0.4 and with a recessive genotype relative risk of 2 would result in a power of 0.75 using a recessive genetic model. The calculated powers are adjusted using the same multiple hypothesis testing correction as described in Methods. (PDF) [file pone.0069843.s004.pdf]
