# Supplementary material for: Roles of Genetic Polymorphisms in the Folate Pathway in Childhood Acute Lymphoblastic Leukemia Evaluated by Bayesian Relevance and Effect Size Analysis
Source: PLoS One. 2013 Aug 5;8(8):e69843. doi: 10.1371/journal.pone.0069843 (PMC3734218; doi:10.1371/journal.pone.0069843)
Supplement: Table S1 — Functions and names of the key enzymes and transporter molecules involved in the folate metabolism. (DOC) [file pone.0069843.s005.doc]

**Table S1 Functions and names of the key enzymes and transporter molecules involved in the folate metabolism**

| **Transporters and enzymes** | | **Functions** |
| --- | --- | --- |
| ABCB1 | ATP-binding cassette, sub-family B (MDR/TAP), member 1 | Transport folate across extra- and intra-cellular membranes |
| DHFR | Dihydrofolate reductase | Converts dihydrofolate into reduced tetrahydrofolate |
| FPGS | Folylpoly-gamma-glutamyl-synthetase | Catalyzes conversion of folates to polyglutamate derivatives |
| GGH | Gamma-glutamyl-hydrolase | Catalyzes the hydrolysis of folylpoly-gamma-glutamates to yield pteroyl-alpha-glutamate (folic acid) and free glutamate |
| GSTP1 | Glutathione-S-transferase pi | Catalyzes the conjugation of several hydrophobic and electrophilic compounds with reduced glutathione, thus play an important role in detoxification and DNA repair |
| MTHFD1 | Methylenetetrahydrofolate dehydrogenase (NADP+ dependent) 1 | Catalyzes the interconversion of derivatives of tetrahydrofolate:10-formyltetrahydrofolate, 5,10‑methenyltetrahydrofolate, and 5,10‑methylenetetrahydrofolate |
| MTHFR | 5,10-methylenetetrahydrofolate reductase (NAD(P)H) | Catalyzes the conversion of 5,10‑methylenetetrahydrofolate to 5‑methyltetrahydrofolate, a co-substrate for homocysteine remethylation to methionine |
| MTR | 5-methyltetrahydrofolate-homocysteine methyltransferase, Vitamin-B12 dependent methionine synthase | Remethylates homocysteine using methyltetrahydrofolate and Vitamin B12 yielding methionine |
| MTRR | 5-methyltetrahydrofolate-homocysteine methyltransferase reductase | Catalyzes the reductive regeneration of the MTR's cofactor B12 to maintain the appropriate function of MTR |
| SHMT1 | Serine hydroxymethyltransferase 1 | Catalyzes the reversible conversion of serine and tetrahydrofolate to glycine and 5,10‑methylene tetrahydrofolate |
| SLC19A1 | Solute carrier family 19, member1, Reduced folate carrier (=RFC) | Transport folate across extra- and intracellular membranes |
| SLC22A8* | Solute carrier family 22, member8 | Plays an important role in the transport and excretion of endogenous and exogenous organic molecules (e.g. methotrexate) especially from the kidney |
| SLCO1B1* | Solute carrier family 21, member6 (=SLC21A6) | Transporter molecule involved in the uptake/removal of organic anions, certain drug compounds, mainly expressed in the liver |
| TPMT | Thiopurine-S-methyltransferase | Metabolizes thiopurine drugs via S-adenosyl-L-methionine as the S-methyl donor and S-adenosyl-L-homocysteine as a byproduct |
| TYMS | Thymidilate synthase | Catalyzes the methylation of deoxyuridylate to deoxythymidylate using the cofactor of 5,10‑methylenetetrahydrofolate, involved in the maintaining of the dTMP pool pivotal for DNA replication and repair |
