# Supplementary material for: Roles of Genetic Polymorphisms in the Folate Pathway in Childhood Acute Lymphoblastic Leukemia Evaluated by Bayesian Relevance and Effect Size Analysis
Source: PLoS One. 2013 Aug 5;8(8):e69843. doi: 10.1371/journal.pone.0069843 (PMC3734218; doi:10.1371/journal.pone.0069843)
Supplement: Table S2 — Hardy–Weinberg equilibrium test for each of the studied polymorphisms. (DOC) [file pone.0069843.s006.doc]

**Table S2 Hardy–Weinberg equilibrium test for each of the studied polymorphisms**

| **Gene** | **SNP ID** | **Tests for deviation from Hardy-Weinberg equilibrium (Pearson)** | | **Tests for association (C.I.: 95% confidence interval)** | | | |
| --- | --- | --- | --- | --- | --- | --- | --- |
|  |  | **P_Controls_** | **P_Cases_** | **Allele frequency difference** | **Heterozygous** | **Homozygous** | **Allele positivity** |
| ABCB1 | rs10280101 |  |  | **Risk allele 2** | | | |
|  |  |  |  |  |  |  |  |
|  |  |  |  |  |  |  |  |
|  |  |  |  | **[1]<->[2]** | **[11]<->[12]** | **[11+]<->[22]** | **[11]<->[12+22]** |
|  |  |  |  | Odds_ratio=0.783 | Odds_ratio=0.861 | Odds_ratio=0.408 | Odds_ratio=0.812 |
|  |  | 0.026603 | 0.659938 | C.I.=[0.575-1.066] | C.I.=[0.608-1.218] | C.I.=[0.127-1.312] | C.I.=[0.580-1.136] |
|  |  |  |  | chi2=2.42 | chi2=0.72 | chi2=2.41 | chi2=1.48 |
|  |  |  |  | p=0.12004 (P) | p=0.39671 | p=0.12061 | p=0.22344 |
|  |  |  |  | **Risk allele 1** | | | |
|  |  |  |  | **[2]<->[1]** | **[22]<->[12]** | **[22]<->[11]** | **[11+12]<->[22]** |
|  |  |  |  | Odds_ratio=1.277 | Odds_ratio=2.108 | Odds_ratio=2.450 | Odds_ratio=2.391 |
|  |  |  |  | C.I.=[0.938-1.739] | C.I.=[0.634-7.017] | C.I.=[0.762-7.877] | C.I.=[0.745-7.677] |
|  |  |  |  | chi2=2.42 | chi2=1.53 | chi2=2.41 | chi2=2.28 |
|  |  |  |  | p=0.12004 (P) | p=0.21549 | p=0.12061 | p=0.13112 |
| ABCB1 | rs1202179 |  |  | **Risk allele 2** | | | |
|  |  |  |  |  |  |  |  |
|  |  |  |  |  |  |  |  |
|  |  |  |  | **[1]<->[2]** | **[11]<->[12]** | **[11+]<->[22]** | **[11]<->[12+22]** |
|  |  |  |  | Odds_ratio=0.968 | Odds_ratio=0.946 | Odds_ratio=0.972 | Odds_ratio=0.950 |
|  |  | 0.973012 | 0.669065 | C.I.=[0.803-1.169] | C.I.=[0.735-1.217] | C.I.=[0.621-1.521] | C.I.=[0.747-1.208] |
|  |  |  |  | chi2=0.11 | chi2=0.19 | chi2=0.02 | chi2=0.17 |
|  |  |  |  | p=0.73785 (P) | p=0.66510 | p=0.90032 | p=0.67643 |
|  |  |  |  | **Risk allele 1** | | | |
|  |  |  |  | **[2]<->[1]** | **[22]<->[12]** | **[22]<->[11]** | **[11+12]<->[22]** |
|  |  |  |  | Odds_ratio=1.033 | Odds_ratio=0.973 | Odds_ratio=1.029 | Odds_ratio=1.004 |
|  |  |  |  | C.I.=[0.856-1.246] | C.I.=[0.617-1.536] | C.I.=[0.657-1.611] | C.I.=[0.651-1.550] |
|  |  |  |  | chi2=0.11 | chi2=0.01 | chi2=0.02 | chi2=0.00 |
|  |  |  |  | p=0.73785 (P) | p=0.90731 | p=0.90032 | p=0.98528 |
| ABCB1 | rs2235013 |  |  | **Risk allele 2** | | | |
|  |  |  |  |  |  |  |  |
|  |  |  |  |  |  |  |  |
|  |  |  |  | **[1]<->[2]** | **[11]<->[12]** | **[11+]<->[22]** | **[11]<->[12+22]** |
|  |  |  |  | Odds_ratio=0.938 | Odds_ratio=0.890 | Odds_ratio=0.884 | Odds_ratio=0.888 |
|  |  | 0.665128 | 0.284978 | C.I.=[0.791-1.112] | C.I.=[0.664-1.193] | C.I.=[0.632-1.236] | C.I.=[0.675-1.169] |
|  |  |  |  | chi2=0.54 | chi2=0.60 | chi2=0.52 | chi2=0.72 |
|  |  |  |  | p=0.46196 (P) | p=0.43716 | p=0.47010 | p=0.39704 |
|  |  |  |  | **Risk allele 1** | | | |
|  |  |  |  | **[2]<->[1]** | **[22]<->[12]** | **[22]<->[11]** | **[11+12]<->[22]** |
|  |  |  |  | Odds_ratio=1.066 | Odds_ratio=1.007 | Odds_ratio=1.131 | Odds_ratio=1.049 |
|  |  |  |  | C.I.=[0.899-1.264] | C.I.=[0.751-1.351] | C.I.=[0.809-1.581] | C.I.=[0.797-1.381] |
|  |  |  |  | chi2=0.54 | chi2=0.00 | chi2=0.52 | chi2=0.12 |
|  |  |  |  | p=0.46196 (P) | p=0.96099 | p=0.47010 | p=0.73333 |
| ABCB1 | rs9282564 |  |  | **Risk allele 2** | | | |
|  |  |  |  |  |  |  |  |
|  |  |  |  |  |  |  |  |
|  |  |  |  | **[1]<->[2]** | **[11]<->[12]** | **[11+]<->[22]** | **[11]<->[12+22]** |
|  |  |  |  | Odds_ratio=0.800 | Odds_ratio=0.796 | Odds_ratio=0.623 | Odds_ratio=0.787 |
|  |  | 0.515533 | 0.567792 | C.I.=[0.608-1.052] | C.I.=[0.588-1.077] | C.I.=[0.175-2.225] | C.I.=[0.585-1.060] |
|  |  |  |  | chi2=2.56 | chi2=2.19 | chi2=0.54 | chi2=2.49 |
|  |  |  |  | p=0.10934 (P) | p=0.13872 | p=0.46274 | p=0.11422 |
|  |  |  |  | **Risk allele 1** | | | |
|  |  |  |  | **[2]<->[1]** | **[22]<->[12]** | **[22]<->[11]** | **[11+12]<->[22]** |
|  |  |  |  | Odds_ratio=1.251 | Odds_ratio=1.276 | Odds_ratio=1.604 | Odds_ratio=1.532 |
|  |  |  |  | C.I.=[0.951-1.645] | C.I.=[0.350-4.654] | C.I.=[0.449-5.725] | C.I.=[0.430-5.460] |
|  |  |  |  | chi2=2.56 | chi2=0.14 | chi2=0.54 | chi2=0.44 |
|  |  |  |  | p=0.10934 (P) | p=0.71108 | p=0.46274 | p=0.50762 |
| CYP1A1 | rs2470893 |  |  | **Risk allele 2** | | | |
|  |  |  |  |  |  |  |  |
|  |  |  |  |  |  |  |  |
|  |  |  |  | **[1]<->[2]** | **[11]<->[12]** | **[11+]<->[22]** | **[11]<->[12+22]** |
|  |  |  |  | Odds_ratio=0.919 | Odds_ratio=1.000 | Odds_ratio=0.718 | Odds_ratio=0.953 |
|  |  | 0.481613 | 0.453224 | C.I.=[0.757-1.116] | C.I.=[0.777-1.288] | C.I.=[0.435-1.183] | C.I.=[0.749-1.213] |
|  |  |  |  | chi2=0.72 | chi2=0.00 | chi2=1.70 | chi2=0.15 |
|  |  |  |  | p=0.39531 (P) | p=0.99856 | p=0.19216 | p=0.69546 |
|  |  |  |  | **Risk allele 1** | | | |
|  |  |  |  | **[2]<->[1]** | **[22]<->[12]** | **[22]<->[11]** | **[11+12]<->[22]** |
|  |  |  |  | Odds_ratio=1.088 | Odds_ratio=1.394 | Odds_ratio=1.393 | Odds_ratio=1.393 |
|  |  |  |  | C.I.=[0.896-1.321] | C.I.=[0.835-2.325] | C.I.=[0.845-2.297] | C.I.=[0.854-2.273] |
|  |  |  |  | chi2=0.72 | chi2=1.63 | chi2=1.70 | chi2=1.78 |
|  |  |  |  | p=0.39531 (P) | p=0.20233 | p=0.19216 | p=0.18228 |
| CYP3A4 | rs12333983 |  |  | **Risk allele 2** | | | |
|  |  |  |  |  |  |  |  |
|  |  |  |  |  |  |  |  |
|  |  |  |  | **[1]<->[2]** | **[11]<->[12]** | **[11+]<->[22]** | **[11]<->[12+22]** |
|  |  |  |  | Odds_ratio=1.225 | Odds_ratio=1.188 | Odds_ratio=1.804 | Odds_ratio=1.219 |
|  |  | 0.956864 | 0.570688 | C.I.=[0.929-1.615] | C.I.=[0.872-1.618] | C.I.=[0.600-5.430] | C.I.=[0.902-1.648] |
|  |  |  |  | chi2=2.08 | chi2=1.19 | chi2=1.13 | chi2=1.67 |
|  |  |  |  | p=0.14913 (P) | p=0.27547 | p=0.28707 | p=0.19657 |
|  |  |  |  | **Risk allele 1** | | | |
|  |  |  |  | **[2]<->[1]** | **[22]<->[12]** | **[22]<->[11]** | **[11+12]<->[22]** |
|  |  |  |  | Odds_ratio=0.816 | Odds_ratio=0.658 | Odds_ratio=0.554 | Odds_ratio=0.573 |
|  |  |  |  | C.I.=[0.619-1.076] | C.I.=[0.213-2.033] | C.I.=[0.184-1.668] | C.I.=[0.191-1.722] |
|  |  |  |  | chi2=2.08 | chi2=0.53 | chi2=1.13 | chi2=1.01 |
|  |  |  |  | p=0.14913 (P) | p=0.46468 | p=0.28707 | p=0.31541 |
| CYP3A4 | rs2246709 |  |  | **Risk allele 2** | | | |
|  |  |  |  |  |  |  |  |
|  |  |  |  |  |  |  |  |
|  |  |  |  | **[1]<->[2]** | **[11]<->[12]** | **[11+]<->[22]** | **[11]<->[12+22]** |
|  |  |  |  | Odds_ratio=1.021 | Odds_ratio=0.960 | Odds_ratio=1.168 | Odds_ratio=0.989 |
|  |  | 0.425634 | 0.793478 | C.I.=[0.844-1.237] | C.I.=[0.747-1.234] | C.I.=[0.721-1.895] | C.I.=[0.778-1.257] |
|  |  |  |  | chi2=0.05 | chi2=0.10 | chi2=0.40 | chi2=0.01 |
|  |  |  |  | p=0.82729 (P) | p=0.74932 | p=0.52753 | p=0.92475 |
|  |  |  |  | **Risk allele 1** | | | |
|  |  |  |  | **[2]<->[1]** | **[22]<->[12]** | **[22]<->[11]** | **[11+12]<->[22]** |
|  |  |  |  | Odds_ratio=0.979 | Odds_ratio=0.822 | Odds_ratio=0.856 | Odds_ratio=0.841 |
|  |  |  |  | C.I.=[0.809-1.185] | C.I.=[0.502-1.345] | C.I.=[0.528-1.388] | C.I.=[0.525-1.347] |
|  |  |  |  | chi2=0.05 | chi2=0.61 | chi2=0.40 | chi2=0.52 |
|  |  |  |  | p=0.82729 (P) | p=0.43370 | p=0.52753 | p=0.47096 |
| CYP3A4 | rs2404955 |  |  | **Risk allele 2** | | | |
|  |  |  |  |  |  |  |  |
|  |  |  |  |  |  |  |  |
|  |  |  |  | **[1]<->[2]** | **[11]<->[12]** | **[11+]<->[22]** | **[11]<->[12+22]** |
|  |  |  |  | Odds_ratio=1.128 | Odds_ratio=1.148 | Odds_ratio=1.130 | Odds_ratio=1.147 |
|  |  | 0.424247 | 0.655090 | C.I.=[0.864-1.474] | C.I.=[0.848-1.555] | C.I.=[0.432-2.958] | C.I.=[0.855-1.539] |
|  |  |  |  | chi2=0.78 | chi2=0.80 | chi2=0.06 | chi2=0.84 |
|  |  |  |  | p=0.37622 (P) | p=0.37115 | p=0.80265 | p=0.36047 |
|  |  |  |  | **Risk allele 1** | | | |
|  |  |  |  | **[2]<->[1]** | **[22]<->[12]** | **[22]<->[11]** | **[11+12]<->[22]** |
|  |  |  |  | Odds_ratio=0.886 | Odds_ratio=1.016 | Odds_ratio=0.885 | Odds_ratio=0.910 |
|  |  |  |  | C.I.=[0.678-1.158] | C.I.=[0.377-2.735] | C.I.=[0.338-2.315] | C.I.=[0.348-2.376] |
|  |  |  |  | chi2=0.78 | chi2=0.00 | chi2=0.06 | chi2=0.04 |
|  |  |  |  | p=0.37622 (P) | p=0.97513 | p=0.80265 | p=0.84664 |
| DHFR | rs11742668 |  |  | **Risk allele 2** | | | |
|  |  |  |  |  |  |  |  |
|  |  |  |  |  |  |  |  |
|  |  |  |  | **[1]<->[2]** | **[11]<->[12]** | **[11+]<->[22]** | **[11]<->[12+22]** |
|  |  |  |  | Odds_ratio=0.833 | Odds_ratio=0.725 | Odds_ratio=2.428 | Odds_ratio=0.772 |
|  |  | 0.631286 | 0.019092 | C.I.=[0.589-1.178] | C.I.=[0.496-1.059] | C.I.=[0.469-12.582] | C.I.=[0.534-1.116] |
|  |  |  |  | chi2=1.07 | chi2=2.78 | chi2=1.19 | chi2=1.90 |
|  |  |  |  | p=0.30114 (P) | p=0.09561 | p=0.27511 | p=0.16780 |
|  |  |  |  | **Risk allele 1** | | | |
|  |  |  |  | **[2]<->[1]** | **[22]<->[12]** | **[22]<->[11]** | **[11+12]<->[22]** |
|  |  |  |  | Odds_ratio=1.200 | Odds_ratio=0.299 | Odds_ratio=0.412 | Odds_ratio=0.396 |
|  |  |  |  | C.I.=[0.849-1.698] | C.I.=[0.056-1.599] | C.I.=[0.079-2.134] | C.I.=[0.076-2.051] |
|  |  |  |  | chi2=1.07 | chi2=2.21 | chi2=1.19 | chi2=1.31 |
|  |  |  |  | p=0.30114 (P) | p=0.13712 | p=0.27511 | p=0.25306 |
| DHFR | rs1222809 |  |  | **Risk allele 2** | | | |
|  |  |  |  |  |  |  |  |
|  |  |  |  |  |  |  |  |
|  |  |  |  | **[1]<->[2]** | **[11]<->[12]** | **[11+]<->[22]** | **[11]<->[12+22]** |
|  |  |  |  | Odds_ratio=0.961 | Odds_ratio=0.945 | Odds_ratio=0.954 | Odds_ratio=0.946 |
|  |  | 0.953067 | 0.722263 | C.I.=[0.791-1.167] | C.I.=[0.734-1.216] | C.I.=[0.585-1.556] | C.I.=[0.743-1.204] |
|  |  |  |  | chi2=0.16 | chi2=0.20 | chi2=0.04 | chi2=0.20 |
|  |  |  |  | p=0.68661 (P) | p=0.65829 | p=0.85016 | p=0.65166 |
|  |  |  |  | **Risk allele 1** | | | |
|  |  |  |  | **[2]<->[1]** | **[22]<->[12]** | **[22]<->[11]** | **[11+12]<->[22]** |
|  |  |  |  | Odds_ratio=1.041 | Odds_ratio=0.990 | Odds_ratio=1.048 | Odds_ratio=1.024 |
|  |  |  |  | C.I.=[0.857-1.264] | C.I.=[0.600-1.635] | C.I.=[0.643-1.710] | C.I.=[0.635-1.653] |
|  |  |  |  | chi2=0.16 | chi2=0.00 | chi2=0.04 | chi2=0.01 |
|  |  |  |  | p=0.68661 (P) | p=0.96913 | p=0.85016 | p=0.92150 |
| DHFR | rs12517451 |  |  | **Risk allele 2** | | | |
|  |  |  |  |  |  |  |  |
|  |  |  |  |  |  |  |  |
|  |  |  |  | **[1]<->[2]** | **[11]<->[12]** | **[11+]<->[22]** | **[11]<->[12+22]** |
|  |  |  |  | Odds_ratio=1.109 | Odds_ratio=1.128 | Odds_ratio=1.187 | Odds_ratio=1.136 |
|  |  | 0.672864 | 0.893544 | C.I.=[0.912-1.348] | C.I.=[0.875-1.452] | C.I.=[0.725-1.943] | C.I.=[0.893-1.446] |
|  |  |  |  | chi2=1.08 | chi2=0.86 | chi2=0.46 | chi2=1.08 |
|  |  |  |  | p=0.29847 (P) | p=0.35264 | p=0.49618 | p=0.29976 |
|  |  |  |  | **Risk allele 1** | | | |
|  |  |  |  | **[2]<->[1]** | **[22]<->[12]** | **[22]<->[11]** | **[11+12]<->[22]** |
|  |  |  |  | Odds_ratio=0.902 | Odds_ratio=0.950 | Odds_ratio=0.843 | Odds_ratio=0.884 |
|  |  |  |  | C.I.=[0.742-1.096] | C.I.=[0.573-1.576] | C.I.=[0.515-1.380] | C.I.=[0.546-1.433] |
|  |  |  |  | chi2=1.08 | chi2=0.04 | chi2=0.46 | chi2=0.25 |
|  |  |  |  | p=0.29847 (P) | p=0.84332 | p=0.49618 | p=0.61691 |
| DHFR | rs1478834 |  |  | **Risk allele 2** | | | |
|  |  |  |  |  |  |  |  |
|  |  |  |  |  |  |  |  |
|  |  |  |  | **[1]<->[2]** | **[11]<->[12]** | **[11+]<->[22]** | **[11]<->[12+22]** |
|  |  |  |  | Odds_ratio=0.968 | Odds_ratio=0.969 | Odds_ratio=0.934 | Odds_ratio=0.964 |
|  |  | 0.878848 | 0.896973 | C.I.=[0.798-1.173] | C.I.=[0.753-1.247] | C.I.=[0.578-1.511] | C.I.=[0.758-1.226] |
|  |  |  |  | chi2=0.11 | chi2=0.06 | chi2=0.08 | chi2=0.09 |
|  |  |  |  | p=0.73757 (P) | p=0.80727 | p=0.78235 | p=0.76310 |
|  |  |  |  | **Risk allele 1** | | | |
|  |  |  |  | **[2]<->[1]** | **[22]<->[12]** | **[22]<->[11]** | **[11+12]<->[22]** |
|  |  |  |  | Odds_ratio=1.033 | Odds_ratio=1.037 | Odds_ratio=1.070 | Odds_ratio=1.056 |
|  |  |  |  | C.I.=[0.852-1.253] | C.I.=[0.634-1.696] | C.I.=[0.662-1.731] | C.I.=[0.661-1.689] |
|  |  |  |  | chi2=0.11 | chi2=0.02 | chi2=0.08 | chi2=0.05 |
|  |  |  |  | p=0.73757 (P) | p=0.88481 | p=0.78235 | p=0.81905 |
| DHFR | rs1650723 |  |  | **Risk allele 2** | | | |
|  |  |  |  |  |  |  |  |
|  |  |  |  |  |  |  |  |
|  |  |  |  | **[1]<->[2]** | **[11]<->[12]** | **[11+]<->[22]** | **[11]<->[12+22]** |
|  |  |  |  | Odds_ratio=0.946 | Odds_ratio=0.857 | Odds_ratio=1.466 | Odds_ratio=0.893 |
|  |  | 0.270293 | 0.386933 | C.I.=[0.746-1.201] | C.I.=[0.650-1.129] | C.I.=[0.627-3.427] | C.I.=[0.683-1.167] |
|  |  |  |  | chi2=0.21 | chi2=1.21 | chi2=0.79 | chi2=0.69 |
|  |  |  |  | p=0.65036 (P) | p=0.27206 | p=0.37469 | p=0.40656 |
|  |  |  |  | **Risk allele 1** | | | |
|  |  |  |  | **[2]<->[1]** | **[22]<->[12]** | **[22]<->[11]** | **[11+12]<->[22]** |
|  |  |  |  | Odds_ratio=1.057 | Odds_ratio=0.584 | Odds_ratio=0.682 | Odds_ratio=0.655 |
|  |  |  |  | C.I.=[0.833-1.340] | C.I.=[0.245-1.396] | C.I.=[0.292-1.595] | C.I.=[0.281-1.527] |
|  |  |  |  | chi2=0.21 | chi2=1.49 | chi2=0.79 | chi2=0.97 |
|  |  |  |  | p=0.65036 (P) | p=0.22208 | p=0.37469 | p=0.32406 |
| DHFR | rs1677626 |  |  | **Risk allele 2** | | | |
|  |  |  |  |  |  |  |  |
|  |  |  |  |  |  |  |  |
|  |  |  |  | **[1]<->[2]** | **[11]<->[12]** | **[11+]<->[22]** | **[11]<->[12+22]** |
|  |  |  |  | Odds_ratio=0.958 | Odds_ratio=0.964 | Odds_ratio=0.909 | Odds_ratio=0.956 |
|  |  | 0.878848 | 0.960432 | C.I.=[0.790-1.162] | C.I.=[0.749-1.241] | C.I.=[0.560-1.475] | C.I.=[0.751-1.216] |
|  |  |  |  | chi2=0.19 | chi2=0.08 | chi2=0.15 | chi2=0.14 |
|  |  |  |  | p=0.66664 (P) | p=0.77842 | p=0.69979 | p=0.71284 |
|  |  |  |  | **Risk allele 1** | | | |
|  |  |  |  | **[2]<->[1]** | **[22]<->[12]** | **[22]<->[11]** | **[11+12]<->[22]** |
|  |  |  |  | Odds_ratio=1.043 | Odds_ratio=1.061 | Odds_ratio=1.100 | Odds_ratio=1.084 |
|  |  |  |  | C.I.=[0.860-1.265] | C.I.=[0.646-1.740] | C.I.=[0.678-1.784] | C.I.=[0.676-1.738] |
|  |  |  |  | chi2=0.19 | chi2=0.05 | chi2=0.15 | chi2=0.11 |
|  |  |  |  | p=0.66664 (P) | p=0.81553 | p=0.69979 | p=0.73932 |
| FPGS | rs10106 |  |  | **Risk allele 2** | | | |
|  |  |  |  |  |  |  |  |
|  |  |  |  |  |  |  |  |
|  |  |  |  | **[1]<->[2]** | **[11]<->[12]** | **[11+]<->[22]** | **[11]<->[12+22]** |
|  |  |  |  | Odds_ratio=1.027 | Odds_ratio=0.963 | Odds_ratio=1.090 | Odds_ratio=0.994 |
|  |  | 0.633751 | 0.173803 | C.I.=[0.861-1.225] | C.I.=[0.739-1.254] | C.I.=[0.758-1.567] | C.I.=[0.775-1.274] |
|  |  |  |  | chi2=0.09 | chi2=0.08 | chi2=0.22 | chi2=0.00 |
|  |  |  |  | p=0.76916 (P) | p=0.77864 | p=0.64278 | p=0.96050 |
|  |  |  |  | **Risk allele 1** | | | |
|  |  |  |  | **[2]<->[1]** | **[22]<->[12]** | **[22]<->[11]** | **[11+12]<->[22]** |
|  |  |  |  | Odds_ratio=0.974 | Odds_ratio=0.884 | Odds_ratio=0.918 | Odds_ratio=0.899 |
|  |  |  |  | C.I.=[0.817-1.162] | C.I.=[0.619-1.261] | C.I.=[0.638-1.320] | C.I.=[0.644-1.256] |
|  |  |  |  | chi2=0.09 | chi2=0.46 | chi2=0.22 | chi2=0.39 |
|  |  |  |  | p=0.76916 (P) | p=0.49535 | p=0.64278 | p=0.53318 |
| FPGS | rs1544105 |  |  | **Risk allele 2** | | | |
|  |  |  |  |  |  |  |  |
|  |  |  |  |  |  |  |  |
|  |  |  |  | **[1]<->[2]** | **[11]<->[12]** | **[11+]<->[22]** | **[11]<->[12+22]** |
|  |  |  |  | Odds_ratio=1.080 | Odds_ratio=1.028 | Odds_ratio=1.190 | Odds_ratio=1.067 |
|  |  | 0.763893 | 0.332334 | C.I.=[0.907-1.285] | C.I.=[0.791-1.337] | C.I.=[0.830-1.705] | C.I.=[0.834-1.366] |
|  |  |  |  | chi2=0.74 | chi2=0.04 | chi2=0.90 | chi2=0.27 |
|  |  |  |  | p=0.38852 (P) | p=0.83561 | p=0.34311 | p=0.60629 |
|  |  |  |  | **Risk allele 1** | | | |
|  |  |  |  | **[2]<->[1]** | **[22]<->[12]** | **[22]<->[11]** | **[11+12]<->[22]** |
|  |  |  |  | Odds_ratio=0.926 | Odds_ratio=0.864 | Odds_ratio=0.840 | Odds_ratio=0.853 |
|  |  |  |  | C.I.=[0.778-1.102] | C.I.=[0.609-1.226] | C.I.=[0.587-1.204] | C.I.=[0.614-1.186] |
|  |  |  |  | chi2=0.74 | chi2=0.67 | chi2=0.90 | chi2=0.89 |
|  |  |  |  | p=0.38852 (P) | p=0.41337 | p=0.34311 | p=0.34511 |
| FPGS | rs4451422 |  |  | **Risk allele 2** | | | |
|  |  |  |  |  |  |  |  |
|  |  |  |  |  |  |  |  |
|  |  |  |  | **[1]<->[2]** | **[11]<->[12]** | **[11+]<->[22]** | **[11]<->[12+22]** |
|  |  |  |  | Odds_ratio=0.990 | Odds_ratio=0.925 | Odds_ratio=1.021 | Odds_ratio=0.949 |
|  |  | 0.717645 | 0.215495 | C.I.=[0.826-1.188] | C.I.=[0.705-1.214] | C.I.=[0.701-1.487] | C.I.=[0.735-1.224] |
|  |  |  |  | chi2=0.01 | chi2=0.31 | chi2=0.01 | chi2=0.16 |
|  |  |  |  | p=0.91661 (P) | p=0.57602 | p=0.91378 | p=0.68513 |
|  |  |  |  | **Risk allele 1** | | | |
|  |  |  |  | **[2]<->[1]** | **[22]<->[12]** | **[22]<->[11]** | **[11+12]<->[22]** |
|  |  |  |  | Odds_ratio=1.010 | Odds_ratio=0.906 | Odds_ratio=0.979 | Odds_ratio=0.940 |
|  |  |  |  | C.I.=[0.842-1.211] | C.I.=[0.627-1.311] | C.I.=[0.673-1.426] | C.I.=[0.664-1.329] |
|  |  |  |  | chi2=0.01 | chi2=0.27 | chi2=0.01 | chi2=0.12 |
|  |  |  |  | p=0.91661 (P) | p=0.60191 | p=0.91378 | p=0.72512 |
| GGH | rs10957267 |  |  | **Risk allele 2** | | | |
|  |  |  |  |  |  |  |  |
|  |  |  |  |  |  |  |  |
|  |  |  |  | **[1]<->[2]** | **[11]<->[12]** | **[11+]<->[22]** | **[11]<->[12+22]** |
|  |  |  |  | Odds_ratio=0.950 | Odds_ratio=0.874 | Odds_ratio=1.294 | Odds_ratio=0.905 |
|  |  | 0.629603 | 0.255429 | C.I.=[0.749-1.206] | C.I.=[0.661-1.155] | C.I.=[0.587-2.853] | C.I.=[0.691-1.184] |
|  |  |  |  | chi2=0.18 | chi2=0.90 | chi2=0.41 | chi2=0.53 |
|  |  |  |  | p=0.67359 (P) | p=0.34292 | p=0.52210 | p=0.46574 |
|  |  |  |  | **Risk allele 1** | | | |
|  |  |  |  | **[2]<->[1]** | **[22]<->[12]** | **[22]<->[11]** | **[11+12]<->[22]** |
|  |  |  |  | Odds_ratio=1.053 | Odds_ratio=0.675 | Odds_ratio=0.773 | Odds_ratio=0.746 |
|  |  |  |  | C.I.=[0.829-1.336] | C.I.=[0.299-1.524] | C.I.=[0.350-1.704] | C.I.=[0.340-1.640] |
|  |  |  |  | chi2=0.18 | chi2=0.90 | chi2=0.41 | chi2=0.53 |
|  |  |  |  | p=0.67359 (P) | p=0.34229 | p=0.52210 | p=0.46493 |
| GGH | rs11545078 |  |  | **Risk allele 2** | | | |
|  |  |  |  |  |  |  |  |
|  |  |  |  |  |  |  |  |
|  |  |  |  | **[1]<->[2]** | **[11]<->[12]** | **[11+]<->[22]** | **[11]<->[12+22]** |
|  |  |  |  | Odds_ratio=0.923 | Odds_ratio=0.939 | Odds_ratio=0.642 | Odds_ratio=0.928 |
|  |  | 0.773077 | 0.500539 | C.I.=[0.674-1.265] | C.I.=[0.670-1.317] | C.I.=[0.107-3.861] | C.I.=[0.665-1.296] |
|  |  |  |  | chi2=0.25 | chi2=0.13 | chi2=0.24 | chi2=0.19 |
|  |  |  |  | p=0.61861 (P) | p=0.71595 | p=0.62571 | p=0.66263 |
|  |  |  |  | **Risk allele 1** | | | |
|  |  |  |  | **[2]<->[1]** | **[22]<->[12]** | **[22]<->[11]** | **[11+12]<->[22]** |
|  |  |  |  | Odds_ratio=1.083 | Odds_ratio=1.462 | Odds_ratio=1.557 | Odds_ratio=1.543 |
|  |  |  |  | C.I.=[0.791-1.485] | C.I.=[0.238-8.992] | C.I.=[0.259-9.363] | C.I.=[0.257-9.270] |
|  |  |  |  | chi2=0.25 | chi2=0.17 | chi2=0.24 | chi2=0.23 |
|  |  |  |  | p=0.61861 (P) | p=0.67997 | p=0.62571 | p=0.63296 |
| GGH | rs3780127 |  |  | **Risk allele 2** | | | |
|  |  |  |  |  |  |  |  |
|  |  |  |  |  |  |  |  |
|  |  |  |  | **[1]<->[2]** | **[11]<->[12]** | **[11+]<->[22]** | **[11]<->[12+22]** |
|  |  |  |  | Odds_ratio=0.923 | Odds_ratio=0.939 | Odds_ratio=0.642 | Odds_ratio=0.928 |
|  |  | 0.773077 | 0.500539 | C.I.=[0.674-1.265] | C.I.=[0.670-1.317] | C.I.=[0.107-3.861] | C.I.=[0.665-1.296] |
|  |  |  |  | chi2=0.25 | chi2=0.13 | chi2=0.24 | chi2=0.19 |
|  |  |  |  | p=0.61861 (P) | p=0.71595 | p=0.62571 | p=0.66263 |
|  |  |  |  | **Risk allele 1** | | | |
|  |  |  |  | **[2]<->[1]** | **[22]<->[12]** | **[22]<->[11]** | **[11+12]<->[22]** |
|  |  |  |  | Odds_ratio=1.083 | Odds_ratio=1.462 | Odds_ratio=1.557 | Odds_ratio=1.543 |
|  |  |  |  | C.I.=[0.791-1.485] | C.I.=[0.238-8.992] | C.I.=[0.259-9.363] | C.I.=[0.257-9.270] |
|  |  |  |  | chi2=0.25 | chi2=0.17 | chi2=0.24 | chi2=0.23 |
|  |  |  |  | p=0.61861 (P) | p=0.67997 | p=0.62571 | p=0.63296 |
| GGH | rs719235 |  |  | **Risk allele 2** | | | |
|  |  |  |  |  |  |  |  |
|  |  |  |  |  |  |  |  |
|  |  |  |  | **[1]<->[2]** | **[11]<->[12]** | **[11+]<->[22]** | **[11]<->[12+22]** |
|  |  |  |  | Odds_ratio=0.911 | Odds_ratio=0.967 | Odds_ratio=0.771 | Odds_ratio=0.929 |
|  |  | 0.264252 | 0.860491 | C.I.=[0.754-1.101] | C.I.=[0.749-1.248] | C.I.=[0.493-1.203] | C.I.=[0.729-1.183] |
|  |  |  |  | chi2=0.93 | chi2=0.07 | chi2=1.32 | chi2=0.36 |
|  |  |  |  | p=0.33521 (P) | p=0.79787 | p=0.25089 | p=0.54840 |
|  |  |  |  | **Risk allele 1** | | | |
|  |  |  |  | **[2]<->[1]** | **[22]<->[12]** | **[22]<->[11]** | **[11+12]<->[22]** |
|  |  |  |  | Odds_ratio=1.098 | Odds_ratio=1.255 | Odds_ratio=1.298 | Odds_ratio=1.279 |
|  |  |  |  | C.I.=[0.908-1.327] | C.I.=[0.796-1.979] | C.I.=[0.831-2.027] | C.I.=[0.831-1.970] |
|  |  |  |  | chi2=0.93 | chi2=0.96 | chi2=1.32 | chi2=1.25 |
|  |  |  |  | p=0.33521 (P) | p=0.32746 | p=0.25089 | p=0.26285 |
| GSTP1 | rs1695 |  |  | **Risk allele 2** | | | |
|  |  |  |  |  |  |  |  |
|  |  |  |  |  |  |  |  |
|  |  |  |  | **[1]<->[2]** | **[11]<->[12]** | **[11+]<->[22]** | **[11]<->[12+22]** |
|  |  |  |  | Odds_ratio=1.036 | Odds_ratio=1.113 | Odds_ratio=0.980 | Odds_ratio=1.086 |
|  |  | 0.148166 | 0.760109 | C.I.=[0.860-1.248] | C.I.=[0.864-1.435] | C.I.=[0.641-1.496] | C.I.=[0.855-1.380] |
|  |  |  |  | chi2=0.14 | chi2=0.69 | chi2=0.01 | chi2=0.46 |
|  |  |  |  | p=0.70822 (P) | p=0.40769 | p=0.92377 | p=0.49944 |
|  |  |  |  | **Risk allele 1** | | | |
|  |  |  |  | **[2]<->[1]** | **[22]<->[12]** | **[22]<->[11]** | **[11+12]<->[22]** |
|  |  |  |  | Odds_ratio=0.965 | Odds_ratio=1.136 | Odds_ratio=1.021 | Odds_ratio=1.071 |
|  |  |  |  | C.I.=[0.802-1.162] | C.I.=[0.738-1.750] | C.I.=[0.669-1.559] | C.I.=[0.712-1.610] |
|  |  |  |  | chi2=0.14 | chi2=0.34 | chi2=0.01 | chi2=0.11 |
|  |  |  |  | p=0.70822 (P) | p=0.56168 | p=0.92377 | p=0.74344 |
| GSTP1 | rs749174 |  |  | **Risk allele 2** | | | |
|  |  |  |  |  |  |  |  |
|  |  |  |  |  |  |  |  |
|  |  |  |  | **[1]<->[2]** | **[11]<->[12]** | **[11+]<->[22]** | **[11]<->[12+22]** |
|  |  |  |  | Odds_ratio=1.017 | Odds_ratio=1.063 | Odds_ratio=0.977 | Odds_ratio=1.046 |
|  |  | 0.275701 | 0.699808 | C.I.=[0.845-1.224] | C.I.=[0.825-1.370] | C.I.=[0.638-1.494] | C.I.=[0.823-1.329] |
|  |  |  |  | chi2=0.03 | chi2=0.22 | chi2=0.01 | chi2=0.14 |
|  |  |  |  | p=0.86076 (P) | p=0.63645 | p=0.91363 | p=0.71251 |
|  |  |  |  | **Risk allele 1** | | | |
|  |  |  |  | **[2]<->[1]** | **[22]<->[12]** | **[22]<->[11]** | **[11+12]<->[22]** |
|  |  |  |  | Odds_ratio=0.984 | Odds_ratio=1.088 | Odds_ratio=1.024 | Odds_ratio=1.052 |
|  |  |  |  | C.I.=[0.817-1.184] | C.I.=[0.706-1.679] | C.I.=[0.669-1.566] | C.I.=[0.698-1.585] |
|  |  |  |  | chi2=0.03 | chi2=0.15 | chi2=0.01 | chi2=0.06 |
|  |  |  |  | p=0.86076 (P) | p=0.70179 | p=0.91363 | p=0.80819 |
| GSTP1 | rs7941395 |  |  | **Risk allele 2** | | | |
|  |  |  |  |  |  |  |  |
|  |  |  |  |  |  |  |  |
|  |  |  |  | **[1]<->[2]** | **[11]<->[12]** | **[11+]<->[22]** | **[11]<->[12+22]** |
|  |  |  |  | Odds_ratio=1.016 | Odds_ratio=1.043 | Odds_ratio=1.010 | Odds_ratio=1.035 |
|  |  | 0.410756 | 0.660140 | C.I.=[0.851-1.213] | C.I.=[0.807-1.348] | C.I.=[0.691-1.477] | C.I.=[0.813-1.319] |
|  |  |  |  | chi2=0.03 | chi2=0.10 | chi2=0.00 | chi2=0.08 |
|  |  |  |  | p=0.86132 (P) | p=0.74893 | p=0.95878 | p=0.77875 |
|  |  |  |  | **Risk allele 1** | | | |
|  |  |  |  | **[2]<->[1]** | **[22]<->[12]** | **[22]<->[11]** | **[11+12]<->[22]** |
|  |  |  |  | Odds_ratio=0.984 | Odds_ratio=1.032 | Odds_ratio=0.990 | Odds_ratio=1.011 |
|  |  |  |  | C.I.=[0.824-1.175] | C.I.=[0.707-1.507] | C.I.=[0.677-1.447] | C.I.=[0.708-1.445] |
|  |  |  |  | chi2=0.03 | chi2=0.03 | chi2=0.00 | chi2=0.00 |
|  |  |  |  | p=0.86132 (P) | p=0.86849 | p=0.95878 | p=0.95017 |
| MTHFD1 | rs1076991 |  |  | **Risk allele 2** | | | |
|  |  |  |  |  |  |  |  |
|  |  |  |  |  |  |  |  |
|  |  |  |  | **[1]<->[2]** | **[11]<->[12]** | **[11+]<->[22]** | **[11]<->[12+22]** |
|  |  |  |  | Odds_ratio=1.394 | Odds_ratio=1.488 | Odds_ratio=1.946 | Odds_ratio=1.608 |
|  |  | 0.953385 | 0.414862 | C.I.=[1.175-1.653] | C.I.=[1.123-1.972] | C.I.=[1.372-2.759] | C.I.=[1.232-2.100] |
|  |  |  |  | chi2=14.59 | chi2=7.68 | chi2=14.10 | chi2=12.30 |
|  |  |  |  | p=0.00013 (P) | p=0.00557 | p=0.00017 | p=0.00045 |
|  |  |  |  | **Risk allele 1** | | | |
|  |  |  |  | **[2]<->[1]** | **[22]<->[12]** | **[22]<->[11]** | **[11+12]<->[22]** |
|  |  |  |  | Odds_ratio=0.718 | Odds_ratio=0.765 | Odds_ratio=0.514 | Odds_ratio=0.662 |
|  |  |  |  | C.I.=[0.605-0.851] | C.I.=[0.558-1.049] | C.I.=[0.363-0.729] | C.I.=[0.491-0.893] |
|  |  |  |  | chi2=14.59 | chi2=2.78 | chi2=14.10 | chi2=7.38 |
|  |  |  |  | p=0.00013 (P) | p=0.09570 | p=0.00017 | p=0.00661 |
| MTHFD1 | rs1950902 |  |  | **Risk allele 2** | | | |
|  |  |  |  |  |  |  |  |
|  |  |  |  |  |  |  |  |
|  |  |  |  | **[1]<->[2]** | **[11]<->[12]** | **[11+]<->[22]** | **[11]<->[12+22]** |
|  |  |  |  | Odds_ratio=1.028 | Odds_ratio=1.086 | Odds_ratio=0.882 | Odds_ratio=1.061 |
|  |  | 0.070755 | 0.421555 | C.I.=[0.814-1.299] | C.I.=[0.822-1.436] | C.I.=[0.443-1.755] | C.I.=[0.812-1.385] |
|  |  |  |  | chi2=0.06 | chi2=0.34 | chi2=0.13 | chi2=0.19 |
|  |  |  |  | p=0.81451 (P) | p=0.56230 | p=0.72040 | p=0.66545 |
|  |  |  |  | **Risk allele 1** | | | |
|  |  |  |  | **[2]<->[1]** | **[22]<->[12]** | **[22]<->[11]** | **[11+12]<->[22]** |
|  |  |  |  | Odds_ratio=0.972 | Odds_ratio=1.231 | Odds_ratio=1.134 | Odds_ratio=1.158 |
|  |  |  |  | C.I.=[0.770-1.228] | C.I.=[0.602-2.517] | C.I.=[0.570-2.256] | C.I.=[0.584-2.296] |
|  |  |  |  | chi2=0.06 | chi2=0.33 | chi2=0.13 | chi2=0.18 |
|  |  |  |  | p=0.81451 (P) | p=0.56801 | p=0.72040 | p=0.67399 |
| MTHFD1 | rs2236225 |  |  | **Risk allele 2** | | | |
|  |  |  |  |  |  |  |  |
|  |  |  |  |  |  |  |  |
|  |  |  |  | **[1]<->[2]** | **[11]<->[12]** | **[11+]<->[22]** | **[11]<->[12+22]** |
|  |  |  |  | Odds_ratio=0.885 | Odds_ratio=0.927 | Odds_ratio=0.784 | Odds_ratio=0.882 |
|  |  | 0.119782 | 0.301069 | C.I.=[0.746-1.051] | C.I.=[0.708-1.214] | C.I.=[0.558-1.102] | C.I.=[0.686-1.135] |
|  |  |  |  | chi2=1.94 | chi2=0.30 | chi2=1.96 | chi2=0.95 |
|  |  |  |  | p=0.16353 (P) | p=0.58167 | p=0.16137 | p=0.32980 |
|  |  |  |  | **Risk allele 1** | | | |
|  |  |  |  | **[2]<->[1]** | **[22]<->[12]** | **[22]<->[11]** | **[11+12]<->[22]** |
|  |  |  |  | Odds_ratio=1.130 | Odds_ratio=1.182 | Odds_ratio=1.275 | Odds_ratio=1.221 |
|  |  |  |  | C.I.=[0.952-1.341] | C.I.=[0.854-1.635] | C.I.=[0.907-1.792] | C.I.=[0.902-1.654] |
|  |  |  |  | chi2=1.94 | chi2=1.02 | chi2=1.96 | chi2=1.67 |
|  |  |  |  | p=0.16353 (P) | p=0.31236 | p=0.16137 | p=0.19664 |
| MTHFD1 | rs745686 |  |  | **Risk allele 2** | | | |
|  |  |  |  |  |  |  |  |
|  |  |  |  |  |  |  |  |
|  |  |  |  | **[1]<->[2]** | **[11]<->[12]** | **[11+]<->[22]** | **[11]<->[12+22]** |
|  |  |  |  | Odds_ratio=0.985 | Odds_ratio=0.937 | Odds_ratio=1.024 | Odds_ratio=0.954 |
|  |  | 0.614916 | 0.197983 | C.I.=[0.820-1.182] | C.I.=[0.726-1.209] | C.I.=[0.682-1.536] | C.I.=[0.750-1.213] |
|  |  |  |  | chi2=0.03 | chi2=0.25 | chi2=0.01 | chi2=0.15 |
|  |  |  |  | p=0.86931 (P) | p=0.61681 | p=0.91040 | p=0.70196 |
|  |  |  |  | **Risk allele 1** | | | |
|  |  |  |  | **[2]<->[1]** | **[22]<->[12]** | **[22]<->[11]** | **[11+12]<->[22]** |
|  |  |  |  | Odds_ratio=1.015 | Odds_ratio=0.915 | Odds_ratio=0.977 | Odds_ratio=0.948 |
|  |  |  |  | C.I.=[0.846-1.219] | C.I.=[0.606-1.382] | C.I.=[0.651-1.466] | C.I.=[0.643-1.398] |
|  |  |  |  | chi2=0.03 | chi2=0.18 | chi2=0.01 | chi2=0.07 |
|  |  |  |  | p=0.86931 (P) | p=0.67436 | p=0.91040 | p=0.78757 |
| MTHFR | rs13306561 |  |  | **Risk allele 2** | | | |
|  |  |  |  |  |  |  |  |
|  |  |  |  |  |  |  |  |
|  |  |  |  | **[1]<->[2]** | **[11]<->[12]** | **[11+]<->[22]** | **[11]<->[12+22]** |
|  |  |  |  | Odds_ratio=1.053 | Odds_ratio=1.090 | Odds_ratio=0.848 | Odds_ratio=1.077 |
|  |  | 0.309813 | 0.080695 | C.I.=[0.824-1.345] | C.I.=[0.826-1.437] | C.I.=[0.283-2.547] | C.I.=[0.821-1.414] |
|  |  |  |  | chi2=0.17 | chi2=0.37 | chi2=0.09 | chi2=0.29 |
|  |  |  |  | p=0.68237 (P) | p=0.54316 | p=0.76924 | p=0.59188 |
|  |  |  |  | **Risk allele 1** | | | |
|  |  |  |  | **[2]<->[1]** | **[22]<->[12]** | **[22]<->[11]** | **[11+12]<->[22]** |
|  |  |  |  | Odds_ratio=0.950 | Odds_ratio=1.284 | Odds_ratio=1.179 | Odds_ratio=1.205 |
|  |  |  |  | C.I.=[0.743-1.214] | C.I.=[0.421-3.921] | C.I.=[0.393-3.539] | C.I.=[0.402-3.609] |
|  |  |  |  | chi2=0.17 | chi2=0.19 | chi2=0.09 | chi2=0.11 |
|  |  |  |  | p=0.68237 (P) | p=0.65974 | p=0.76924 | p=0.73872 |
| MTHFR | rs1801131 |  |  | **Risk allele 2** | | | |
|  |  |  |  |  |  |  |  |
|  |  |  |  |  |  |  |  |
|  |  |  |  | **[1]<->[2]** | **[11]<->[12]** | **[11+]<->[22]** | **[11]<->[12+22]** |
|  |  |  |  | Odds_ratio=1.016 | Odds_ratio=1.075 | Odds_ratio=0.961 | Odds_ratio=1.053 |
|  |  | 0.393061 | 0.962483 | C.I.=[0.845-1.223] | C.I.=[0.835-1.384] | C.I.=[0.627-1.473] | C.I.=[0.829-1.338] |
|  |  |  |  | chi2=0.03 | chi2=0.32 | chi2=0.03 | chi2=0.18 |
|  |  |  |  | p=0.86461 (P) | p=0.57408 | p=0.85445 | p=0.67239 |
|  |  |  |  | **Risk allele 1** | | | |
|  |  |  |  | **[2]<->[1]** | **[22]<->[12]** | **[22]<->[11]** | **[11+12]<->[22]** |
|  |  |  |  | Odds_ratio=0.984 | Odds_ratio=1.119 | Odds_ratio=1.041 | Odds_ratio=1.076 |
|  |  |  |  | C.I.=[0.818-1.184] | C.I.=[0.725-1.727] | C.I.=[0.679-1.596] | C.I.=[0.713-1.624] |
|  |  |  |  | chi2=0.03 | chi2=0.26 | chi2=0.03 | chi2=0.12 |
|  |  |  |  | p=0.86461 (P) | p=0.61168 | p=0.85445 | p=0.72843 |
| MTHFR | rs1801133 |  |  | **Risk allele 2** | | | |
|  |  |  |  |  |  |  |  |
|  |  |  |  |  |  |  |  |
|  |  |  |  | **[1]<->[2]** | **[11]<->[12]** | **[11+]<->[22]** | **[11]<->[12+22]** |
|  |  |  |  | Odds_ratio=1.048 | Odds_ratio=1.024 | Odds_ratio=1.118 | Odds_ratio=1.045 |
|  |  | 0.890206 | 0.841392 | C.I.=[0.879-1.250] | C.I.=[0.791-1.326] | C.I.=[0.766-1.631] | C.I.=[0.818-1.334] |
|  |  |  |  | chi2=0.28 | chi2=0.03 | chi2=0.33 | chi2=0.12 |
|  |  |  |  | p=0.59829 (P) | p=0.85434 | p=0.56389 | p=0.72570 |
|  |  |  |  | **Risk allele 1** | | | |
|  |  |  |  | **[2]<->[1]** | **[22]<->[12]** | **[22]<->[11]** | **[11+12]<->[22]** |
|  |  |  |  | Odds_ratio=0.954 | Odds_ratio=0.917 | Odds_ratio=0.895 | Odds_ratio=0.906 |
|  |  |  |  | C.I.=[0.800-1.137] | C.I.=[0.632-1.329] | C.I.=[0.613-1.306] | C.I.=[0.638-1.288] |
|  |  |  |  | chi2=0.28 | chi2=0.21 | chi2=0.33 | chi2=0.30 |
|  |  |  |  | p=0.59829 (P) | p=0.64608 | p=0.56389 | p=0.58376 |
| MTRR | rs10380 |  |  | **Risk allele 2** | | | |
|  |  |  |  |  |  |  |  |
|  |  |  |  |  |  |  |  |
|  |  |  |  | **[1]<->[2]** | **[11]<->[12]** | **[11+]<->[22]** | **[11]<->[12+22]** |
|  |  |  |  | Odds_ratio=0.916 | Odds_ratio=0.932 | Odds_ratio=0.484 | Odds_ratio=0.921 |
|  |  | 0.429624 | 0.231523 | C.I.=[0.664-1.263] | C.I.=[0.663-1.311] | C.I.=[0.044-5.362] | C.I.=[0.657-1.292] |
|  |  |  |  | chi2=0.29 | chi2=0.16 | chi2=0.36 | chi2=0.23 |
|  |  |  |  | p=0.59165 (P) | p=0.68660 | p=0.54604 | p=0.63420 |
|  |  |  |  | **Risk allele 1** | | | |
|  |  |  |  | **[2]<->[1]** | **[22]<->[12]** | **[22]<->[11]** | **[11+12]<->[22]** |
|  |  |  |  | Odds_ratio=1.092 | Odds_ratio=1.924 | Odds_ratio=2.064 | Odds_ratio=2.043 |
|  |  |  |  | C.I.=[0.792-1.506] | C.I.=[0.171-21.660] | C.I.=[0.186-22.845] | C.I.=[0.185-22.596] |
|  |  |  |  | chi2=0.29 | chi2=0.29 | chi2=0.36 | chi2=0.35 |
|  |  |  |  | p=0.59165 (P) | p=0.59002 | p=0.54604 | p=0.55198 |
| MTRR | rs1532268 |  |  | **Risk allele 2** | | | |
|  |  |  |  |  |  |  |  |
|  |  |  |  |  |  |  |  |
|  |  |  |  | **[1]<->[2]** | **[11]<->[12]** | **[11+]<->[22]** | **[11]<->[12+22]** |
|  |  |  |  | Odds_ratio=1.196 | Odds_ratio=1.268 | Odds_ratio=1.350 | Odds_ratio=1.287 |
|  |  | 0.261909 | 0.822746 | C.I.=[1.002-1.427] | C.I.=[0.980-1.642] | C.I.=[0.927-1.966] | C.I.=[1.009-1.641] |
|  |  |  |  | chi2=3.92 | chi2=3.26 | chi2=2.46 | chi2=4.14 |
|  |  |  |  | p=0.04763 (P) | p=0.07087 | p=0.11674 | p=0.04189 |
|  |  |  |  | **Risk allele 1** | | | |
|  |  |  |  | **[2]<->[1]** | **[22]<->[12]** | **[22]<->[11]** | **[11+12]<->[22]** |
|  |  |  |  | Odds_ratio=0.836 | Odds_ratio=0.939 | Odds_ratio=0.741 | Odds_ratio=0.837 |
|  |  |  |  | C.I.=[0.701-0.998] | C.I.=[0.647-1.364] | C.I.=[0.509-1.078] | C.I.=[0.589-1.190] |
|  |  |  |  | chi2=3.92 | chi2=0.11 | chi2=2.46 | chi2=0.98 |
|  |  |  |  | p=0.04763 (P) | p=0.74253 | p=0.11674 | p=0.32123 |
| MTRR | rs162036 |  |  | **Risk allele 2** | | | |
|  |  |  |  |  |  |  |  |
|  |  |  |  |  |  |  |  |
|  |  |  |  | **[1]<->[2]** | **[11]<->[12]** | **[11+]<->[22]** | **[11]<->[12+22]** |
|  |  |  |  | Odds_ratio=0.916 | Odds_ratio=0.988 | Odds_ratio=0.323 | Odds_ratio=0.949 |
|  |  | 0.856199 | 0.147394 | C.I.=[0.690-1.217] | C.I.=[0.725-1.347] | C.I.=[0.065-1.608] | C.I.=[0.700-1.288] |
|  |  |  |  | chi2=0.37 | chi2=0.01 | chi2=2.11 | chi2=0.11 |
|  |  |  |  | p=0.54437 (P) | p=0.93991 | p=0.14613 | p=0.73859 |
|  |  |  |  | **Risk allele 1** | | | |
|  |  |  |  | **[2]<->[1]** | **[22]<->[12]** | **[22]<->[11]** | **[11+12]<->[22]** |
|  |  |  |  | Odds_ratio=1.092 | Odds_ratio=3.062 | Odds_ratio=3.099 | Odds_ratio=3.092 |
|  |  |  |  | C.I.=[0.822-1.450] | C.I.=[0.603-15.543] | C.I.=[0.622-15.437] | C.I.=[0.621-15.388] |
|  |  |  |  | chi2=0.37 | chi2=2.00 | chi2=2.11 | chi2=2.11 |
|  |  |  |  | p=0.54437 (P) | p=0.15720 | p=0.14613 | p=0.14665 |
| MTRR | rs1801394 |  |  | **Risk allele 2** | | | |
|  |  |  |  |  |  |  |  |
|  |  |  |  |  |  |  |  |
|  |  |  |  | **[1]<->[2]** | **[11]<->[12]** | **[11+]<->[22]** | **[11]<->[12+22]** |
|  |  |  |  | Odds_ratio=0.989 | Odds_ratio=1.129 | Odds_ratio=0.954 | Odds_ratio=1.076 |
|  |  | 0.737329 | 0.181714 | C.I.=[0.835-1.173] | C.I.=[0.854-1.494] | C.I.=[0.675-1.349] | C.I.=[0.826-1.402] |
|  |  |  |  | chi2=0.02 | chi2=0.73 | chi2=0.07 | chi2=0.29 |
|  |  |  |  | p=0.90166 (P) | p=0.39386 | p=0.79110 | p=0.58815 |
|  |  |  |  | **Risk allele 1** | | | |
|  |  |  |  | **[2]<->[1]** | **[22]<->[12]** | **[22]<->[11]** | **[11+12]<->[22]** |
|  |  |  |  | Odds_ratio=1.011 | Odds_ratio=1.183 | Odds_ratio=1.048 | Odds_ratio=1.132 |
|  |  |  |  | C.I.=[0.853-1.198] | C.I.=[0.865-1.619] | C.I.=[0.741-1.481] | C.I.=[0.842-1.524] |
|  |  |  |  | chi2=0.02 | chi2=1.11 | chi2=0.07 | chi2=0.68 |
|  |  |  |  | p=0.90166 (P) | p=0.29206 | p=0.79110 | p=0.41088 |
| MTRR | rs2966952 |  |  | **Risk allele 2** | | | |
|  |  |  |  |  |  |  |  |
|  |  |  |  |  |  |  |  |
|  |  |  |  | **[1]<->[2]** | **[11]<->[12]** | **[11+]<->[22]** | **[11]<->[12+22]** |
|  |  |  |  | Odds_ratio=1.018 | Odds_ratio=1.084 | Odds_ratio=0.843 | Odds_ratio=1.055 |
|  |  | 0.417373 | 0.723786 | C.I.=[0.818-1.268] | C.I.=[0.832-1.412] | C.I.=[0.434-1.636] | C.I.=[0.818-1.362] |
|  |  |  |  | chi2=0.03 | chi2=0.35 | chi2=0.26 | chi2=0.17 |
|  |  |  |  | p=0.87072 (P) | p=0.55206 | p=0.61305 | p=0.67791 |
|  |  |  |  | **Risk allele 1** | | | |
|  |  |  |  | **[2]<->[1]** | **[22]<->[12]** | **[22]<->[11]** | **[11+12]<->[22]** |
|  |  |  |  | Odds_ratio=0.982 | Odds_ratio=1.286 | Odds_ratio=1.186 | Odds_ratio=1.216 |
|  |  |  |  | C.I.=[0.788-1.223] | C.I.=[0.649-2.546] | C.I.=[0.611-2.302] | C.I.=[0.630-2.347] |
|  |  |  |  | chi2=0.03 | chi2=0.52 | chi2=0.26 | chi2=0.34 |
|  |  |  |  | p=0.87072 (P) | p=0.47032 | p=0.61305 | p=0.56001 |
| MTRR | rs326120 |  |  | **Risk allele 2** | | | |
|  |  |  |  |  |  |  |  |
|  |  |  |  |  |  |  |  |
|  |  |  |  | **[1]<->[2]** | **[11]<->[12]** | **[11+]<->[22]** | **[11]<->[12+22]** |
|  |  |  |  | Odds_ratio=1.016 | Odds_ratio=1.113 | Odds_ratio=0.762 | Odds_ratio=1.070 |
|  |  | 0.322100 | 0.478413 | C.I.=[0.817-1.265] | C.I.=[0.854-1.449] | C.I.=[0.391-1.484] | C.I.=[0.829-1.380] |
|  |  |  |  | chi2=0.02 | chi2=0.63 | chi2=0.64 | chi2=0.27 |
|  |  |  |  | p=0.88491 (P) | p=0.42869 | p=0.42284 | p=0.60337 |
|  |  |  |  | **Risk allele 1** | | | |
|  |  |  |  | **[2]<->[1]** | **[22]<->[12]** | **[22]<->[11]** | **[11+12]<->[22]** |
|  |  |  |  | Odds_ratio=0.984 | Odds_ratio=1.460 | Odds_ratio=1.312 | Odds_ratio=1.356 |
|  |  |  |  | C.I.=[0.791-1.225] | C.I.=[0.735-2.901] | C.I.=[0.674-2.557] | C.I.=[0.700-2.629] |
|  |  |  |  | chi2=0.02 | chi2=1.18 | chi2=0.64 | chi2=0.82 |
|  |  |  |  | p=0.88491 (P) | p=0.27767 | p=0.42284 | p=0.36490 |
| MTRR | rs3776455 |  |  | **Risk allele 2** | | | |
|  |  |  |  |  |  |  |  |
|  |  |  |  |  |  |  |  |
|  |  |  |  | **[1]<->[2]** | **[11]<->[12]** | **[11+]<->[22]** | **[11]<->[12+22]** |
|  |  |  |  | Odds_ratio=0.828 | Odds_ratio=1.078 | Odds_ratio=0.567 | Odds_ratio=0.931 |
|  |  | 0.013710 | 0.189496 | C.I.=[0.692-0.991] | C.I.=[0.830-1.401] | C.I.=[0.383-0.838] | C.I.=[0.728-1.192] |
|  |  |  |  | chi2=4.24 | chi2=0.32 | chi2=8.19 | chi2=0.32 |
|  |  |  |  | p=0.03938 (P) | p=0.57280 | p=0.00420 | p=0.57129 |
|  |  |  |  | **Risk allele 1** | | | |
|  |  |  |  | **[2]<->[1]** | **[22]<->[12]** | **[22]<->[11]** | **[11+12]<->[22]** |
|  |  |  |  | Odds_ratio=1.208 | Odds_ratio=1.902 | Odds_ratio=1.764 | Odds_ratio=1.834 |
|  |  |  |  | C.I.=[1.009-1.446] | C.I.=[1.290-2.804] | C.I.=[1.193-2.608] | C.I.=[1.270-2.647] |
|  |  |  |  | chi2=4.24 | chi2=10.70 | chi2=8.19 | chi2=10.70 |
|  |  |  |  | p=0.03938 (P) | p=0.00107 | p=0.00420 | p=0.00107 |
| MTR | rs10925257 |  |  | **Risk allele 2** | | | |
|  |  |  |  |  |  |  |  |
|  |  |  |  |  |  |  |  |
|  |  |  |  | **[1]<->[2]** | **[11]<->[12]** | **[11+]<->[22]** | **[11]<->[12+22]** |
|  |  |  |  | Odds_ratio=1.051 | Odds_ratio=1.089 | Odds_ratio=0.997 | Odds_ratio=1.078 |
|  |  | 0.502897 | 0.986940 | C.I.=[0.851-1.298] | C.I.=[0.839-1.414] | C.I.=[0.549-1.812] | C.I.=[0.840-1.384] |
|  |  |  |  | chi2=0.21 | chi2=0.41 | chi2=0.00 | chi2=0.35 |
|  |  |  |  | p=0.64506 (P) | p=0.51955 | p=0.99233 | p=0.55624 |
|  |  |  |  | **Risk allele 1** | | | |
|  |  |  |  | **[2]<->[1]** | **[22]<->[12]** | **[22]<->[11]** | **[11+12]<->[22]** |
|  |  |  |  | Odds_ratio=0.952 | Odds_ratio=1.093 | Odds_ratio=1.003 | Odds_ratio=1.032 |
|  |  |  |  | C.I.=[0.770-1.176] | C.I.=[0.590-2.023] | C.I.=[0.552-1.822] | C.I.=[0.571-1.863] |
|  |  |  |  | chi2=0.21 | chi2=0.08 | chi2=0.00 | chi2=0.01 |
|  |  |  |  | p=0.64506 (P) | p=0.77808 | p=0.99233 | p=0.91726 |
| MTR | rs12759827 |  |  | **Risk allele 2** | | | |
|  |  |  |  |  |  |  |  |
|  |  |  |  |  |  |  |  |
|  |  |  |  | **[1]<->[2]** | **[11]<->[12]** | **[11+]<->[22]** | **[11]<->[12+22]** |
|  |  |  |  | Odds_ratio=0.818 | Odds_ratio=0.819 | Odds_ratio=0.677 | Odds_ratio=0.795 |
|  |  | 0.730959 | 0.720923 | C.I.=[0.674-0.993] | C.I.=[0.636-1.055] | C.I.=[0.418-1.095] | C.I.=[0.624-1.012] |
|  |  |  |  | chi2=4.13 | chi2=2.39 | chi2=2.55 | chi2=3.49 |
|  |  |  |  | p=0.04214 (P) | p=0.12179 | p=0.11043 | p=0.06176 |
|  |  |  |  | **Risk allele 1** | | | |
|  |  |  |  | **[2]<->[1]** | **[22]<->[12]** | **[22]<->[11]** | **[11+12]<->[22]** |
|  |  |  |  | Odds_ratio=1.222 | Odds_ratio=1.210 | Odds_ratio=1.478 | Odds_ratio=1.362 |
|  |  |  |  | C.I.=[1.007-1.483] | C.I.=[0.739-1.982] | C.I.=[0.913-2.392] | C.I.=[0.851-2.180] |
|  |  |  |  | chi2=4.13 | chi2=0.57 | chi2=2.55 | chi2=1.67 |
|  |  |  |  | p=0.04214 (P) | p=0.44886 | p=0.11043 | p=0.19648 |
| MTR | rs1805087 |  |  | **Risk allele 2** | | | |
|  |  |  |  |  |  |  |  |
|  |  |  |  |  |  |  |  |
|  |  |  |  | **[1]<->[2]** | **[11]<->[12]** | **[11+]<->[22]** | **[11]<->[12+22]** |
|  |  |  |  | Odds_ratio=1.026 | Odds_ratio=1.064 | Odds_ratio=0.957 | Odds_ratio=1.050 |
|  |  | 0.335861 | 0.755170 | C.I.=[0.832-1.267] | C.I.=[0.820-1.382] | C.I.=[0.536-1.709] | C.I.=[0.818-1.348] |
|  |  |  |  | chi2=0.06 | chi2=0.22 | chi2=0.02 | chi2=0.15 |
|  |  |  |  | p=0.80797 (P) | p=0.63952 | p=0.88243 | p=0.70107 |
|  |  |  |  | **Risk allele 1** | | | |
|  |  |  |  | **[2]<->[1]** | **[22]<->[12]** | **[22]<->[11]** | **[11+12]<->[22]** |
|  |  |  |  | Odds_ratio=0.974 | Odds_ratio=1.112 | Odds_ratio=1.045 | Odds_ratio=1.066 |
|  |  |  |  | C.I.=[0.789-1.203] | C.I.=[0.611-2.025] | C.I.=[0.585-1.866] | C.I.=[0.601-1.892] |
|  |  |  |  | chi2=0.06 | chi2=0.12 | chi2=0.02 | chi2=0.05 |
|  |  |  |  | p=0.80797 (P) | p=0.72852 | p=0.88243 | p=0.82587 |
| MTR | rs2853523 |  |  | **Risk allele 2** | | | |
|  |  |  |  |  |  |  |  |
|  |  |  |  |  |  |  |  |
|  |  |  |  | **[1]<->[2]** | **[11]<->[12]** | **[11+]<->[22]** | **[11]<->[12+22]** |
|  |  |  |  | Odds_ratio=0.858 | Odds_ratio=0.937 | Odds_ratio=0.702 | Odds_ratio=0.873 |
|  |  | 0.570338 | 0.505311 | C.I.=[0.722-1.021] | C.I.=[0.720-1.219] | C.I.=[0.487-1.011] | C.I.=[0.681-1.120] |
|  |  |  |  | chi2=2.98 | chi2=0.23 | chi2=3.63 | chi2=1.14 |
|  |  |  |  | p=0.08451 (P) | p=0.62852 | p=0.05671 | p=0.28561 |
|  |  |  |  | **Risk allele 1** | | | |
|  |  |  |  | **[2]<->[1]** | **[22]<->[12]** | **[22]<->[11]** | **[11+12]<->[22]** |
|  |  |  |  | Odds_ratio=1.165 | Odds_ratio=1.335 | Odds_ratio=1.425 | Odds_ratio=1.373 |
|  |  |  |  | C.I.=[0.979-1.385] | C.I.=[0.939-1.899] | C.I.=[0.989-2.052] | C.I.=[0.984-1.916] |
|  |  |  |  | chi2=2.98 | chi2=2.60 | chi2=3.63 | chi2=3.50 |
|  |  |  |  | p=0.08451 (P) | p=0.10708 | p=0.05671 | p=0.06125 |
| MTR | rs3768142 |  |  | **Risk allele 2** | | | |
|  |  |  |  |  |  |  |  |
|  |  |  |  |  |  |  |  |
|  |  |  |  | **[1]<->[2]** | **[11]<->[12]** | **[11+]<->[22]** | **[11]<->[12+22]** |
|  |  |  |  | Odds_ratio=0.871 | Odds_ratio=0.961 | Odds_ratio=0.719 | Odds_ratio=0.895 |
|  |  | 0.580446 | 0.404293 | C.I.=[0.732-1.035] | C.I.=[0.738-1.252] | C.I.=[0.500-1.033] | C.I.=[0.697-1.149] |
|  |  |  |  | chi2=2.47 | chi2=0.09 | chi2=3.19 | chi2=0.76 |
|  |  |  |  | p=0.11619 (P) | p=0.76942 | p=0.07411 | p=0.38278 |
|  |  |  |  | **Risk allele 1** | | | |
|  |  |  |  | **[2]<->[1]** | **[22]<->[12]** | **[22]<->[11]** | **[11+12]<->[22]** |
|  |  |  |  | Odds_ratio=1.149 | Odds_ratio=1.337 | Odds_ratio=1.391 | Odds_ratio=1.360 |
|  |  |  |  | C.I.=[0.966-1.366] | C.I.=[0.944-1.895] | C.I.=[0.968-2.000] | C.I.=[0.978-1.891] |
|  |  |  |  | chi2=2.47 | chi2=2.68 | chi2=3.19 | chi2=3.36 |
|  |  |  |  | p=0.11619 (P) | p=0.10158 | p=0.07411 | p=0.06694 |
| MTR | rs4659724 |  |  | **Risk allele 2** | | | |
|  |  |  |  |  |  |  |  |
|  |  |  |  |  |  |  |  |
|  |  |  |  | **[1]<->[2]** | **[11]<->[12]** | **[11+]<->[22]** | **[11]<->[12+22]** |
|  |  |  |  | Odds_ratio=1.100 | Odds_ratio=1.168 | Odds_ratio=1.163 | Odds_ratio=1.167 |
|  |  | 0.570070 | 0.157726 | C.I.=[0.919-1.316] | C.I.=[0.899-1.517] | C.I.=[0.779-1.736] | C.I.=[0.909-1.498] |
|  |  |  |  | chi2=1.07 | chi2=1.35 | chi2=0.54 | chi2=1.47 |
|  |  |  |  | p=0.29997 (P) | p=0.24567 | p=0.46073 | p=0.22604 |
|  |  |  |  | **Risk allele 1** | | | |
|  |  |  |  | **[2]<->[1]** | **[22]<->[12]** | **[22]<->[11]** | **[11+12]<->[22]** |
|  |  |  |  | Odds_ratio=0.909 | Odds_ratio=1.004 | Odds_ratio=0.860 | Odds_ratio=0.936 |
|  |  |  |  | C.I.=[0.760-1.088] | C.I.=[0.678-1.487] | C.I.=[0.576-1.284] | C.I.=[0.644-1.361] |
|  |  |  |  | chi2=1.07 | chi2=0.00 | chi2=0.54 | chi2=0.12 |
|  |  |  |  | p=0.29997 (P) | p=0.98280 | p=0.46073 | p=0.73078 |
| SHMT1 | rs1979277 |  |  | **Risk allele 2** | | | |
|  |  |  |  |  |  |  |  |
|  |  |  |  |  |  |  |  |
|  |  |  |  | **[1]<->[2]** | **[11]<->[12]** | **[11+]<->[22]** | **[11]<->[12+22]** |
|  |  |  |  | Odds_ratio=1.051 | Odds_ratio=1.113 | Odds_ratio=1.023 | Odds_ratio=1.097 |
|  |  | 0.739525 | 0.552053 | C.I.=[0.874-1.265] | C.I.=[0.865-1.432] | C.I.=[0.660-1.587] | C.I.=[0.863-1.394] |
|  |  |  |  | chi2=0.28 | chi2=0.70 | chi2=0.01 | chi2=0.57 |
|  |  |  |  | p=0.59540 (P) | p=0.40412 | p=0.91886 | p=0.44902 |
|  |  |  |  | **Risk allele 1** | | | |
|  |  |  |  | **[2]<->[1]** | **[22]<->[12]** | **[22]<->[11]** | **[11+12]<->[22]** |
|  |  |  |  | Odds_ratio=0.951 | Odds_ratio=1.088 | Odds_ratio=0.977 | Odds_ratio=1.027 |
|  |  |  |  | C.I.=[0.790-1.145] | C.I.=[0.698-1.697] | C.I.=[0.630-1.516] | C.I.=[0.673-1.568] |
|  |  |  |  | chi2=0.28 | chi2=0.14 | chi2=0.01 | chi2=0.02 |
|  |  |  |  | p=0.59540 (P) | p=0.71005 | p=0.91886 | p=0.90193 |
| SHMT1 | rs643333 |  |  | **Risk allele 2** | | | |
|  |  |  |  |  |  |  |  |
|  |  |  |  |  |  |  |  |
|  |  |  |  | **[1]<->[2]** | **[11]<->[12]** | **[11+]<->[22]** | **[11]<->[12+22]** |
|  |  |  |  | Odds_ratio=1.128 | Odds_ratio=1.158 | Odds_ratio=1.222 | Odds_ratio=1.168 |
|  |  | 0.973314 | 0.686528 | C.I.=[0.933-1.362] | C.I.=[0.900-1.489] | C.I.=[0.768-1.946] | C.I.=[0.919-1.485] |
|  |  |  |  | chi2=1.55 | chi2=1.30 | chi2=0.72 | chi2=1.60 |
|  |  |  |  | p=0.21297 (P) | p=0.25362 | p=0.39664 | p=0.20535 |
|  |  |  |  | **Risk allele 1** | | | |
|  |  |  |  | **[2]<->[1]** | **[22]<->[12]** | **[22]<->[11]** | **[11+12]<->[22]** |
|  |  |  |  | Odds_ratio=0.887 | Odds_ratio=0.947 | Odds_ratio=0.818 | Odds_ratio=0.872 |
|  |  |  |  | C.I.=[0.734-1.071] | C.I.=[0.590-1.520] | C.I.=[0.514-1.302] | C.I.=[0.555-1.370] |
|  |  |  |  | chi2=1.55 | chi2=0.05 | chi2=0.72 | chi2=0.35 |
|  |  |  |  | p=0.21297 (P) | p=0.82179 | p=0.39664 | p=0.55343 |
| SHMT1 | rs9909104 |  |  | **Risk allele 2** | | | |
|  |  |  |  |  |  |  |  |
|  |  |  |  |  |  |  |  |
|  |  |  |  | **[1]<->[2]** | **[11]<->[12]** | **[11+]<->[22]** | **[11]<->[12+22]** |
|  |  |  |  | Odds_ratio=0.873 | Odds_ratio=0.766 | Odds_ratio=1.012 | Odds_ratio=0.794 |
|  |  | 0.125872 | 0.450496 | C.I.=[0.716-1.063] | C.I.=[0.595-0.986] | C.I.=[0.595-1.722] | C.I.=[0.623-1.012] |
|  |  |  |  | chi2=1.83 | chi2=4.28 | chi2=0.00 | chi2=3.47 |
|  |  |  |  | p=0.17633 (P) | p=0.03852 | p=0.96509 | p=0.06251 |
|  |  |  |  | **Risk allele 1** | | | |
|  |  |  |  | **[2]<->[1]** | **[22]<->[12]** | **[22]<->[11]** | **[11+12]<->[22]** |
|  |  |  |  | Odds_ratio=1.146 | Odds_ratio=0.757 | Odds_ratio=0.988 | Odds_ratio=0.889 |
|  |  |  |  | C.I.=[0.941-1.396] | C.I.=[0.439-1.304] | C.I.=[0.581-1.682] | C.I.=[0.527-1.498] |
|  |  |  |  | chi2=1.83 | chi2=1.01 | chi2=0.00 | chi2=0.20 |
|  |  |  |  | p=0.17633 (P) | p=0.31463 | p=0.96509 | p=0.65840 |
| SLC19A1 | rs1051266 |  |  | **Risk allele 2** | | | |
|  |  |  |  |  |  |  |  |
|  |  |  |  |  |  |  |  |
|  |  |  |  | **[1]<->[2]** | **[11]<->[12]** | **[11+]<->[22]** | **[11]<->[12+22]** |
|  |  |  |  | Odds_ratio=0.989 | Odds_ratio=1.065 | Odds_ratio=0.958 | Odds_ratio=1.034 |
|  |  | 0.690714 | 0.573269 | C.I.=[0.834-1.174] | C.I.=[0.810-1.400] | C.I.=[0.676-1.357] | C.I.=[0.799-1.338] |
|  |  |  |  | chi2=0.02 | chi2=0.20 | chi2=0.06 | chi2=0.06 |
|  |  |  |  | p=0.90033 (P) | p=0.65222 | p=0.80735 | p=0.80021 |
|  |  |  |  | **Risk allele 1** | | | |
|  |  |  |  | **[2]<->[1]** | **[22]<->[12]** | **[22]<->[11]** | **[11+12]<->[22]** |
|  |  |  |  | Odds_ratio=1.011 | Odds_ratio=1.112 | Odds_ratio=1.044 | Odds_ratio=1.085 |
|  |  |  |  | C.I.=[0.852-1.200] | C.I.=[0.804-1.537] | C.I.=[0.737-1.479] | C.I.=[0.799-1.473] |
|  |  |  |  | chi2=0.02 | chi2=0.41 | chi2=0.06 | chi2=0.27 |
|  |  |  |  | p=0.90033 (P) | p=0.52036 | p=0.80735 | p=0.60059 |
| SLC19A1 | rs4819128 |  |  | **Risk allele 2** | | | |
|  |  |  |  |  |  |  |  |
|  |  |  |  |  |  |  |  |
|  |  |  |  | **[1]<->[2]** | **[11]<->[12]** | **[11+]<->[22]** | **[11]<->[12+22]** |
|  |  |  |  | Odds_ratio=1.013 | Odds_ratio=1.038 | Odds_ratio=1.016 | Odds_ratio=1.032 |
|  |  | 0.505158 | 0.732610 | C.I.=[0.852-1.203] | C.I.=[0.792-1.360] | C.I.=[0.717-1.440] | C.I.=[0.800-1.331] |
|  |  |  |  | chi2=0.02 | chi2=0.07 | chi2=0.01 | chi2=0.06 |
|  |  |  |  | p=0.88680 (P) | p=0.78755 | p=0.92911 | p=0.80975 |
|  |  |  |  | **Risk allele 1** | | | |
|  |  |  |  | **[2]<->[1]** | **[22]<->[12]** | **[22]<->[11]** | **[11+12]<->[22]** |
|  |  |  |  | Odds_ratio=0.988 | Odds_ratio=1.022 | Odds_ratio=0.984 | Odds_ratio=1.006 |
|  |  |  |  | C.I.=[0.831-1.173] | C.I.=[0.734-1.422] | C.I.=[0.695-1.395] | C.I.=[0.737-1.373] |
|  |  |  |  | chi2=0.02 | chi2=0.02 | chi2=0.01 | chi2=0.00 |
|  |  |  |  | p=0.88680 (P) | p=0.89914 | p=0.92911 | p=0.97017 |
| SLC19A1 | rs7499 |  |  | **Risk allele 2** | | | |
|  |  |  |  |  |  |  |  |
|  |  |  |  |  |  |  |  |
|  |  |  |  | **[1]<->[2]** | **[11]<->[12]** | **[11+]<->[22]** | **[11]<->[12+22]** |
|  |  |  |  | Odds_ratio=1.162 | Odds_ratio=1.182 | Odds_ratio=1.331 | Odds_ratio=1.219 |
|  |  | 0.635382 | 0.826464 | C.I.=[0.977-1.382] | C.I.=[0.907-1.541] | C.I.=[0.931-1.904] | C.I.=[0.949-1.565] |
|  |  |  |  | chi2=2.87 | chi2=1.53 | chi2=2.46 | chi2=2.41 |
|  |  |  |  | p=0.09007 (P) | p=0.21667 | p=0.11660 | p=0.12076 |
|  |  |  |  | **Risk allele 1** | | | |
|  |  |  |  | **[2]<->[1]** | **[22]<->[12]** | **[22]<->[11]** | **[11+12]<->[22]** |
|  |  |  |  | Odds_ratio=0.861 | Odds_ratio=0.888 | Odds_ratio=0.751 | Odds_ratio=0.826 |
|  |  |  |  | C.I.=[0.724-1.024] | C.I.=[0.629-1.253] | C.I.=[0.525-1.074] | C.I.=[0.597-1.142] |
|  |  |  |  | chi2=2.87 | chi2=0.46 | chi2=2.46 | chi2=1.34 |
|  |  |  |  | p=0.09007 (P) | p=0.49874 | p=0.11660 | p=0.24746 |
| SLC22A8 | rs2276299 |  |  | **Risk allele 2** | | | |
|  |  |  |  |  |  |  |  |
|  |  |  |  |  |  |  |  |
|  |  |  |  | **[1]<->[2]** | **[11]<->[12]** | **[11+]<->[22]** | **[11]<->[12+22]** |
|  |  |  |  | Odds_ratio=0.977 | Odds_ratio=0.875 | Odds_ratio=1.502 | Odds_ratio=0.917 |
|  |  | 0.112537 | 0.593469 | C.I.=[0.784-1.218] | C.I.=[0.673-1.138] | C.I.=[0.718-3.139] | C.I.=[0.711-1.183] |
|  |  |  |  | chi2=0.04 | chi2=0.99 | chi2=1.18 | chi2=0.45 |
|  |  |  |  | p=0.83623 (P) | p=0.31922 | p=0.27699 | p=0.50355 |
|  |  |  |  | **Risk allele 1** | | | |
|  |  |  |  | **[2]<->[1]** | **[22]<->[12]** | **[22]<->[11]** | **[11+12]<->[22]** |
|  |  |  |  | Odds_ratio=1.024 | Odds_ratio=0.583 | Odds_ratio=0.666 | Odds_ratio=0.639 |
|  |  |  |  | C.I.=[0.821-1.276] | C.I.=[0.274-1.240] | C.I.=[0.319-1.392] | C.I.=[0.307-1.330] |
|  |  |  |  | chi2=0.04 | chi2=2.00 | chi2=1.18 | chi2=1.46 |
|  |  |  |  | p=0.83623 (P) | p=0.15701 | p=0.27699 | p=0.22730 |
| SLC22A8 | rs3809069 |  |  | **Risk allele 2** | | | |
|  |  |  |  |  |  |  |  |
|  |  |  |  |  |  |  |  |
|  |  |  |  | **[1]<->[2]** | **[11]<->[12]** | **[11+]<->[22]** | **[11]<->[12+22]** |
|  |  |  |  | Odds_ratio=1.020 | Odds_ratio=1.053 | Odds_ratio=0.928 | Odds_ratio=1.040 |
|  |  | 0.721352 | 0.813674 | C.I.=[0.816-1.275] | C.I.=[0.806-1.374] | C.I.=[0.462-1.866] | C.I.=[0.804-1.345] |
|  |  |  |  | chi2=0.03 | chi2=0.14 | chi2=0.04 | chi2=0.09 |
|  |  |  |  | p=0.85925 (P) | p=0.70596 | p=0.83467 | p=0.76556 |
|  |  |  |  | **Risk allele 1** | | | |
|  |  |  |  | **[2]<->[1]** | **[22]<->[12]** | **[22]<->[11]** | **[11+12]<->[22]** |
|  |  |  |  | Odds_ratio=0.980 | Odds_ratio=1.134 | Odds_ratio=1.077 | Odds_ratio=1.094 |
|  |  |  |  | C.I.=[0.784-1.225] | C.I.=[0.553-2.325] | C.I.=[0.536-2.165] | C.I.=[0.547-2.188] |
|  |  |  |  | chi2=0.03 | chi2=0.12 | chi2=0.04 | chi2=0.06 |
|  |  |  |  | p=0.85925 (P) | p=0.73174 | p=0.83467 | p=0.80023 |
| SLC22A8 | rs4149183 |  |  | **Risk allele 2** | | | |
|  |  |  |  |  |  |  |  |
|  |  |  |  |  |  |  |  |
|  |  |  |  | **[1]<->[2]** | **[11]<->[12]** | **[11+]<->[22]** | **[11]<->[12+22]** |
|  |  |  |  | Odds_ratio=1.132 | Odds_ratio=1.244 | Odds_ratio=1.007 | Odds_ratio=1.212 |
|  |  | 0.529229 | 0.283039 | C.I.=[0.923-1.388] | C.I.=[0.963-1.606] | C.I.=[0.569-1.782] | C.I.=[0.949-1.550] |
|  |  |  |  | chi2=1.41 | chi2=2.80 | chi2=0.00 | chi2=2.37 |
|  |  |  |  | p=0.23423 (P) | p=0.09403 | p=0.97999 | p=0.12358 |
|  |  |  |  | **Risk allele 1** | | | |
|  |  |  |  | **[2]<->[1]** | **[22]<->[12]** | **[22]<->[11]** | **[11+12]<->[22]** |
|  |  |  |  | Odds_ratio=0.884 | Odds_ratio=1.235 | Odds_ratio=0.993 | Odds_ratio=1.075 |
|  |  |  |  | C.I.=[0.720-1.084] | C.I.=[0.687-2.217] | C.I.=[0.561-1.756] | C.I.=[0.613-1.887] |
|  |  |  |  | chi2=1.41 | chi2=0.50 | chi2=0.00 | chi2=0.06 |
|  |  |  |  | p=0.23423 (P) | p=0.47992 | p=0.97999 | p=0.80031 |
| SLC21A6 | rs10841769 |  |  | **Risk allele 2** | | | |
|  |  |  |  |  |  |  |  |
|  |  |  |  |  |  |  |  |
|  |  |  |  | **[1]<->[2]** | **[11]<->[12]** | **[11+]<->[22]** | **[11]<->[12+22]** |
|  |  |  |  | Odds_ratio=0.968 | Odds_ratio=0.926 | Odds_ratio=0.940 | Odds_ratio=0.930 |
|  |  | 0.888312 | 0.690351 | C.I.=[0.817-1.147] | C.I.=[0.694-1.234] | C.I.=[0.670-1.319] | C.I.=[0.709-1.220] |
|  |  |  |  | chi2=0.14 | chi2=0.28 | chi2=0.13 | chi2=0.27 |
|  |  |  |  | p=0.70926 (P) | p=0.59900 | p=0.72173 | p=0.60206 |
|  |  |  |  | **Risk allele 1** | | | |
|  |  |  |  | **[2]<->[1]** | **[22]<->[12]** | **[22]<->[11]** | **[11+12]<->[22]** |
|  |  |  |  | Odds_ratio=1.033 | Odds_ratio=0.984 | Odds_ratio=1.063 | Odds_ratio=1.011 |
|  |  |  |  | C.I.=[0.872-1.224] | C.I.=[0.730-1.327] | C.I.=[0.758-1.492] | C.I.=[0.763-1.340] |
|  |  |  |  | chi2=0.14 | chi2=0.01 | chi2=0.13 | chi2=0.01 |
|  |  |  |  | p=0.70926 (P) | p=0.91777 | p=0.72173 | p=0.93797 |
| SLC21A6 | rs11045818 |  |  | **Risk allele 2** | | | |
|  |  |  |  |  |  |  |  |
|  |  |  |  |  |  |  |  |
|  |  |  |  | **[1]<->[2]** | **[11]<->[12]** | **[11+]<->[22]** | **[11]<->[12+22]** |
|  |  |  |  | Odds_ratio=0.889 | Odds_ratio=0.754 | Odds_ratio=2.587 | Odds_ratio=0.806 |
|  |  | 0.029022 | 0.255406 | C.I.=[0.690-1.146] | C.I.=[0.565-1.006] | C.I.=[0.817-8.195] | C.I.=[0.609-1.068] |
|  |  |  |  | chi2=0.83 | chi2=3.70 | chi2=2.81 | chi2=2.25 |
|  |  |  |  | p=0.36337 (P) | p=0.05448 | p=0.09397 | p=0.13325 |
|  |  |  |  | **Risk allele 1** | | | |
|  |  |  |  | **[2]<->[1]** | **[22]<->[12]** | **[22]<->[11]** | **[11+12]<->[22]** |
|  |  |  |  | Odds_ratio=1.125 | Odds_ratio=0.291 | Odds_ratio=0.386 | Odds_ratio=0.362 |
|  |  |  |  | C.I.=[0.873-1.450] | C.I.=[0.090-0.941] | C.I.=[0.122-1.224] | C.I.=[0.114-1.143] |
|  |  |  |  | chi2=0.83 | chi2=4.73 | chi2=2.81 | chi2=3.26 |
|  |  |  |  | p=0.36337 (P) | p=0.02961 | p=0.09397 | p=0.07098 |
| SLC21A6 | rs11045819 |  |  | **Risk allele 2** | | | |
|  |  |  |  |  |  |  |  |
|  |  |  |  |  |  |  |  |
|  |  |  |  | **[1]<->[2]** | **[11]<->[12]** | **[11+]<->[22]** | **[11]<->[12+22]** |
|  |  |  |  | Odds_ratio=0.831 | Odds_ratio=0.730 | Odds_ratio=1.919 | Odds_ratio=0.765 |
|  |  | 0.031875 | 0.642011 | C.I.=[0.640-1.079] | C.I.=[0.544-0.980] | C.I.=[0.573-6.424] | C.I.=[0.574-1.019] |
|  |  |  |  | chi2=1.93 | chi2=4.42 | chi2=1.16 | chi2=3.35 |
|  |  |  |  | p=0.16446 (P) | p=0.03556 | p=0.28250 | p=0.06709 |
|  |  |  |  | **Risk allele 1** | | | |
|  |  |  |  | **[2]<->[1]** | **[22]<->[12]** | **[22]<->[11]** | **[11+12]<->[22]** |
|  |  |  |  | Odds_ratio=1.203 | Odds_ratio=0.381 | Odds_ratio=0.521 | Odds_ratio=0.484 |
|  |  |  |  | C.I.=[0.927-1.563] | C.I.=[0.112-1.299] | C.I.=[0.156-1.745] | C.I.=[0.145-1.619] |
|  |  |  |  | chi2=1.93 | chi2=2.54 | chi2=1.16 | chi2=1.45 |
|  |  |  |  | p=0.16446 (P) | p=0.11075 | p=0.28250 | p=0.22908 |
| SLC21A6 | rs11045823 |  |  | **Risk allele 2** | | | |
|  |  |  |  |  |  |  |  |
|  |  |  |  |  |  |  |  |
|  |  |  |  | **[1]<->[2]** | **[11]<->[12]** | **[11+]<->[22]** | **[11]<->[12+22]** |
|  |  |  |  | Odds_ratio=0.942 | Odds_ratio=0.797 | Odds_ratio=2.878 | Odds_ratio=0.857 |
|  |  | 0.027408 | 0.256898 | C.I.=[0.732-1.211] | C.I.=[0.598-1.062] | C.I.=[0.920-9.003] | C.I.=[0.648-1.133] |
|  |  |  |  | chi2=0.22 | chi2=2.41 | chi2=3.60 | chi2=1.18 |
|  |  |  |  | p=0.64086 (P) | p=0.12070 | p=0.05764 | p=0.27742 |
|  |  |  |  | **Risk allele 1** | | | |
|  |  |  |  | **[2]<->[1]** | **[22]<->[12]** | **[22]<->[11]** | **[11+12]<->[22]** |
|  |  |  |  | Odds_ratio=1.062 | Odds_ratio=0.277 | Odds_ratio=0.347 | Odds_ratio=0.329 |
|  |  |  |  | C.I.=[0.826-1.365] | C.I.=[0.087-0.882] | C.I.=[0.111-1.087] | C.I.=[0.105-1.026] |
|  |  |  |  | chi2=0.22 | chi2=5.29 | chi2=3.60 | chi2=4.05 |
|  |  |  |  | p=0.64086 (P) | p=0.02142 | p=0.05764 | p=0.04425 |
| SLC21A6 | rs17328763 |  |  | **Risk allele 2** | | | |
|  |  |  |  |  |  |  |  |
|  |  |  |  |  |  |  |  |
|  |  |  |  | **[1]<->[2]** | **[11]<->[12]** | **[11+]<->[22]** | **[11]<->[12+22]** |
|  |  |  |  | Odds_ratio=0.868 | Odds_ratio=0.881 | Odds_ratio=0.737 | Odds_ratio=0.864 |
|  |  | 0.289485 | 0.420963 | C.I.=[0.689-1.093] | C.I.=[0.669-1.160] | C.I.=[0.369-1.472] | C.I.=[0.664-1.125] |
|  |  |  |  | chi2=1.45 | chi2=0.81 | chi2=0.75 | chi2=1.17 |
|  |  |  |  | p=0.22778 (P) | p=0.36774 | p=0.38562 | p=0.27891 |
|  |  |  |  | **Risk allele 1** | | | |
|  |  |  |  | **[2]<->[1]** | **[22]<->[12]** | **[22]<->[11]** | **[11+12]<->[22]** |
|  |  |  |  | Odds_ratio=1.152 | Odds_ratio=1.196 | Odds_ratio=1.357 | Odds_ratio=1.312 |
|  |  |  |  | C.I.=[0.915-1.451] | C.I.=[0.584-2.449] | C.I.=[0.679-2.710] | C.I.=[0.659-2.609] |
|  |  |  |  | chi2=1.45 | chi2=0.24 | chi2=0.75 | chi2=0.60 |
|  |  |  |  | p=0.22778 (P) | p=0.62448 | p=0.38562 | p=0.43838 |
| SLC21A6 | rs4149056 |  |  | **Risk allele 2** | | | |
|  |  |  |  |  |  |  |  |
|  |  |  |  |  |  |  |  |
|  |  |  |  | **[1]<->[2]** | **[11]<->[12]** | **[11+]<->[22]** | **[11]<->[12+22]** |
|  |  |  |  | Odds_ratio=0.965 | Odds_ratio=1.031 | Odds_ratio=0.772 | Odds_ratio=0.997 |
|  |  | 0.226141 | 0.998234 | C.I.=[0.777-1.197] | C.I.=[0.792-1.342] | C.I.=[0.415-1.434] | C.I.=[0.774-1.283] |
|  |  |  |  | chi2=0.11 | chi2=0.05 | chi2=0.67 | chi2=0.00 |
|  |  |  |  | p=0.74352 (P) | p=0.82005 | p=0.41144 | p=0.97847 |
|  |  |  |  | **Risk allele 1** | | | |
|  |  |  |  | **[2]<->[1]** | **[22]<->[12]** | **[22]<->[11]** | **[11+12]<->[22]** |
|  |  |  |  | Odds_ratio=1.037 | Odds_ratio=1.336 | Odds_ratio=1.296 | Odds_ratio=1.308 |
|  |  |  |  | C.I.=[0.835-1.287] | C.I.=[0.704-2.535] | C.I.=[0.697-2.408] | C.I.=[0.708-2.418] |
|  |  |  |  | chi2=0.11 | chi2=0.79 | chi2=0.67 | chi2=0.74 |
|  |  |  |  | p=0.74352 (P) | p=0.37414 | p=0.41144 | p=0.39002 |
| SLC21A6 | rs4363657 |  |  | **Risk allele 2** | | | |
|  |  |  |  |  |  |  |  |
|  |  |  |  |  |  |  |  |
|  |  |  |  | **[1]<->[2]** | **[11]<->[12]** | **[11+]<->[22]** | **[11]<->[12+22]** |
|  |  |  |  | Odds_ratio=0.949 | Odds_ratio=1.067 | Odds_ratio=0.691 | Odds_ratio=1.005 |
|  |  | 0.028155 | 0.888760 | C.I.=[0.769-1.171] | C.I.=[0.821-1.388] | C.I.=[0.392-1.218] | C.I.=[0.783-1.291] |
|  |  |  |  | chi2=0.24 | chi2=0.24 | chi2=1.65 | chi2=0.00 |
|  |  |  |  | p=0.62527 (P) | p=0.62728 | p=0.19952 | p=0.96693 |
|  |  |  |  | **Risk allele 1** | | | |
|  |  |  |  | **[2]<->[1]** | **[22]<->[12]** | **[22]<->[11]** | **[11+12]<->[22]** |
|  |  |  |  | Odds_ratio=1.054 | Odds_ratio=1.544 | Odds_ratio=1.446 | Odds_ratio=1.477 |
|  |  |  |  | C.I.=[0.854-1.301] | C.I.=[0.858-2.778] | C.I.=[0.821-2.548] | C.I.=[0.844-2.586] |
|  |  |  |  | chi2=0.24 | chi2=2.12 | chi2=1.65 | chi2=1.88 |
|  |  |  |  | p=0.62527 (P) | p=0.14553 | p=0.19952 | p=0.16983 |
| TPMT | rs2518463 |  |  | **Risk allele 2** | | | |
|  |  |  |  |  |  |  |  |
|  |  |  |  |  |  |  |  |
|  |  |  |  | **[1]<->[2]** | **[11]<->[12]** | **[11+]<->[22]** | **[11]<->[12+22]** |
|  |  |  |  | Odds_ratio=1.137 | Odds_ratio=1.278 | Odds_ratio=1.285 | Odds_ratio=1.281 |
|  |  | 0.389242 | 0.601940 | C.I.=[0.959-1.347] | C.I.=[0.956-1.710] | C.I.=[0.916-1.804] | C.I.=[0.974-1.685] |
|  |  |  |  | chi2=2.20 | chi2=2.74 | chi2=2.11 | chi2=3.14 |
|  |  |  |  | p=0.13839 (P) | p=0.09788 | p=0.14637 | p=0.07660 |
|  |  |  |  | **Risk allele 1** | | | |
|  |  |  |  | **[2]<->[1]** | **[22]<->[12]** | **[22]<->[11]** | **[11+12]<->[22]** |
|  |  |  |  | Odds_ratio=0.880 | Odds_ratio=0.995 | Odds_ratio=0.778 | Odds_ratio=0.914 |
|  |  |  |  | C.I.=[0.742-1.042] | C.I.=[0.739-1.339] | C.I.=[0.554-1.092] | C.I.=[0.691-1.209] |
|  |  |  |  | chi2=2.20 | chi2=0.00 | chi2=2.11 | chi2=0.40 |
|  |  |  |  | p=0.13839 (P) | p=0.97179 | p=0.14637 | p=0.52877 |
| TPMT | rs2842951 |  |  | **Risk allele 2** | | | |
|  |  |  |  |  |  |  |  |
|  |  |  |  |  |  |  |  |
|  |  |  |  | **[1]<->[2]** | **[11]<->[12]** | **[11+]<->[22]** | **[11]<->[12+22]** |
|  |  |  |  | Odds_ratio=1.043 | Odds_ratio=1.075 | Odds_ratio=1.029 | Odds_ratio=1.067 |
|  |  | 0.098049 | 0.245627 | C.I.=[0.858-1.269] | C.I.=[0.832-1.389] | C.I.=[0.644-1.644] | C.I.=[0.837-1.359] |
|  |  |  |  | chi2=0.18 | chi2=0.30 | chi2=0.01 | chi2=0.27 |
|  |  |  |  | p=0.67112 (P) | p=0.58090 | p=0.90487 | p=0.60086 |
|  |  |  |  | **Risk allele 1** | | | |
|  |  |  |  | **[2]<->[1]** | **[22]<->[12]** | **[22]<->[11]** | **[11+12]<->[22]** |
|  |  |  |  | Odds_ratio=0.958 | Odds_ratio=1.045 | Odds_ratio=0.972 | Odds_ratio=0.999 |
|  |  |  |  | C.I.=[0.788-1.166] | C.I.=[0.643-1.697] | C.I.=[0.608-1.553] | C.I.=[0.632-1.580] |
|  |  |  |  | chi2=0.18 | chi2=0.03 | chi2=0.01 | chi2=0.00 |
|  |  |  |  | p=0.67112 (P) | p=0.85993 | p=0.90487 | p=0.99617 |
| TPMT | rs4449636 |  |  | **Risk allele 2** | | | |
|  |  |  |  |  |  |  |  |
|  |  |  |  |  |  |  |  |
|  |  |  |  | **[1]<->[2]** | **[11]<->[12]** | **[11+]<->[22]** | **[11]<->[12+22]** |
|  |  |  |  | Odds_ratio=1.116 | Odds_ratio=1.245 | Odds_ratio=1.236 | Odds_ratio=1.242 |
|  |  | 0.317768 | 0.761916 | C.I.=[0.942-1.322] | C.I.=[0.931-1.664] | C.I.=[0.882-1.731] | C.I.=[0.945-1.632] |
|  |  |  |  | chi2=1.60 | chi2=2.19 | chi2=1.52 | chi2=2.43 |
|  |  |  |  | p=0.20600 (P) | p=0.13857 | p=0.21798 | p=0.11930 |
|  |  |  |  | **Risk allele 1** | | | |
|  |  |  |  | **[2]<->[1]** | **[22]<->[12]** | **[22]<->[11]** | **[11+12]<->[22]** |
|  |  |  |  | Odds_ratio=0.896 | Odds_ratio=1.007 | Odds_ratio=0.809 | Odds_ratio=0.933 |
|  |  |  |  | C.I.=[0.756-1.062] | C.I.=[0.749-1.355] | C.I.=[0.578-1.134] | C.I.=[0.706-1.234] |
|  |  |  |  | chi2=1.60 | chi2=0.00 | chi2=1.52 | chi2=0.23 |
|  |  |  |  | p=0.20600 (P) | p=0.96139 | p=0.21798 | p=0.62801 |
| TYMS | rs1004474 |  |  | **Risk allele 2** | | | |
|  |  |  |  |  |  |  |  |
|  |  |  |  |  |  |  |  |
|  |  |  |  | **[1]<->[2]** | **[11]<->[12]** | **[11+]<->[22]** | **[11]<->[12+22]** |
|  |  |  |  | Odds_ratio=1.000 | Odds_ratio=0.822 | Odds_ratio=1.034 | Odds_ratio=0.880 |
|  |  | 0.115189 | 0.390302 | C.I.=[0.844-1.185] | C.I.=[0.621-1.089] | C.I.=[0.732-1.460] | C.I.=[0.675-1.147] |
|  |  |  |  | chi2=0.00 | chi2=1.86 | chi2=0.04 | chi2=0.90 |
|  |  |  |  | p=0.99800 (P) | p=0.17224 | p=0.84857 | p=0.34358 |
|  |  |  |  | **Risk allele 1** | | | |
|  |  |  |  | **[2]<->[1]** | **[22]<->[12]** | **[22]<->[11]** | **[11+12]<->[22]** |
|  |  |  |  | Odds_ratio=1.000 | Odds_ratio=0.795 | Odds_ratio=0.967 | Odds_ratio=0.853 |
|  |  |  |  | C.I.=[0.844-1.185] | C.I.=[0.582-1.086] | C.I.=[0.685-1.365] | C.I.=[0.636-1.146] |
|  |  |  |  | chi2=0.00 | chi2=2.08 | chi2=0.04 | chi2=1.11 |
|  |  |  |  | p=0.99800 (P) | p=0.14936 | p=0.84857 | p=0.29156 |
| TYMS | rs2612100 |  |  | **Risk allele 2** | | | |
|  |  |  |  |  |  |  |  |
|  |  |  |  |  |  |  |  |
|  |  |  |  | **[1]<->[2]** | **[11]<->[12]** | **[11+]<->[22]** | **[11]<->[12+22]** |
|  |  |  |  | Odds_ratio=1.015 | Odds_ratio=0.951 | Odds_ratio=1.093 | Odds_ratio=0.979 |
|  |  | 0.913328 | 0.373071 | C.I.=[0.849-1.214] | C.I.=[0.737-1.227] | C.I.=[0.738-1.620] | C.I.=[0.769-1.246] |
|  |  |  |  | chi2=0.03 | chi2=0.15 | chi2=0.20 | chi2=0.03 |
|  |  |  |  | p=0.87088 (P) | p=0.69702 | p=0.65654 | p=0.86347 |
|  |  |  |  | **Risk allele 1** | | | |
|  |  |  |  | **[2]<->[1]** | **[22]<->[12]** | **[22]<->[11]** | **[11+12]<->[22]** |
|  |  |  |  | Odds_ratio=0.985 | Odds_ratio=0.869 | Odds_ratio=0.915 | Odds_ratio=0.892 |
|  |  |  |  | C.I.=[0.824-1.178] | C.I.=[0.586-1.289] | C.I.=[0.617-1.356] | C.I.=[0.615-1.294] |
|  |  |  |  | chi2=0.03 | chi2=0.49 | chi2=0.20 | chi2=0.36 |
|  |  |  |  | p=0.87088 (P) | p=0.48608 | p=0.65654 | p=0.54672 |
| TYMS | rs2853533 |  |  | **Risk allele 2** | | | |
|  |  |  |  |  |  |  |  |
|  |  |  |  |  |  |  |  |
|  |  |  |  | **[1]<->[2]** | **[11]<->[12]** | **[11+]<->[22]** | **[11]<->[12+22]** |
|  |  |  |  | Odds_ratio=1.366 | Odds_ratio=1.350 | Odds_ratio=1.805 | Odds_ratio=1.389 |
|  |  | 0.327751 | 0.236661 | C.I.=[1.067-1.750] | C.I.=[1.011-1.801] | C.I.=[0.817-3.991] | C.I.=[1.052-1.833] |
|  |  |  |  | chi2=6.15 | chi2=4.16 | chi2=2.19 | chi2=5.39 |
|  |  |  |  | p=0.01312 (P) | p=0.04146 | p=0.13925 | p=0.02030 |
|  |  |  |  | **Risk allele 1** | | | |
|  |  |  |  | **[2]<->[1]** | **[22]<->[12]** | **[22]<->[11]** | **[11+12]<->[22]** |
|  |  |  |  | Odds_ratio=0.732 | Odds_ratio=0.748 | Odds_ratio=0.554 | Odds_ratio=0.594 |
|  |  |  |  | C.I.=[0.571-0.937] | C.I.=[0.329-1.699] | C.I.=[0.251-1.225] | C.I.=[0.269-1.309] |
|  |  |  |  | chi2=6.15 | chi2=0.48 | chi2=2.19 | chi2=1.70 |
|  |  |  |  | p=0.01312 (P) | p=0.48647 | p=0.13925 | p=0.19171 |
| TYMS | rs2853741 |  |  | **Risk allele 2** | | | |
|  |  |  |  |  |  |  |  |
|  |  |  |  |  |  |  |  |
|  |  |  |  | **[1]<->[2]** | **[11]<->[12]** | **[11+]<->[22]** | **[11]<->[12+22]** |
|  |  |  |  | Odds_ratio=1.155 | Odds_ratio=1.080 | Odds_ratio=1.473 | Odds_ratio=1.139 |
|  |  | 0.359286 | 0.857051 | C.I.=[0.960-1.389] | C.I.=[0.839-1.390] | C.I.=[0.948-2.290] | C.I.=[0.896-1.449] |
|  |  |  |  | chi2=2.34 | chi2=0.36 | chi2=2.98 | chi2=1.13 |
|  |  |  |  | p=0.12610 (P) | p=0.54919 | p=0.08416 | p=0.28812 |
|  |  |  |  | **Risk allele 1** | | | |
|  |  |  |  | **[2]<->[1]** | **[22]<->[12]** | **[22]<->[11]** | **[11+12]<->[22]** |
|  |  |  |  | Odds_ratio=0.866 | Odds_ratio=0.733 | Odds_ratio=0.679 | Odds_ratio=0.704 |
|  |  |  |  | C.I.=[0.720-1.041] | C.I.=[0.470-1.144] | C.I.=[0.437-1.055] | C.I.=[0.460-1.077] |
|  |  |  |  | chi2=2.34 | chi2=1.87 | chi2=2.98 | chi2=2.64 |
|  |  |  |  | p=0.12610 (P) | p=0.17095 | p=0.08416 | p=0.10411 |
| TYMS | rs9967368 |  |  | **Risk allele 2** | | | |
|  |  |  |  |  |  |  |  |
|  |  |  |  |  |  |  |  |
|  |  |  |  | **[1]<->[2]** | **[11]<->[12]** | **[11+]<->[22]** | **[11]<->[12+22]** |
|  |  |  |  | Odds_ratio=1.085 | Odds_ratio=1.131 | Odds_ratio=1.164 | Odds_ratio=1.139 |
|  |  | 0.471583 | 0.215656 | C.I.=[0.910-1.293] | C.I.=[0.862-1.485] | C.I.=[0.803-1.687] | C.I.=[0.879-1.475] |
|  |  |  |  | chi2=0.83 | chi2=0.79 | chi2=0.65 | chi2=0.97 |
|  |  |  |  | p=0.36253 (P) | p=0.37386 | p=0.42163 | p=0.32347 |
|  |  |  |  | **Risk allele 1** | | | |
|  |  |  |  | **[2]<->[1]** | **[22]<->[12]** | **[22]<->[11]** | **[11+12]<->[22]** |
|  |  |  |  | Odds_ratio=0.922 | Odds_ratio=0.972 | Odds_ratio=0.859 | Odds_ratio=0.925 |
|  |  |  |  | C.I.=[0.774-1.098] | C.I.=[0.685-1.379] | C.I.=[0.593-1.245] | C.I.=[0.663-1.291] |
|  |  |  |  | chi2=0.83 | chi2=0.03 | chi2=0.65 | chi2=0.21 |
|  |  |  |  | p=0.36253 (P) | p=0.87234 | p=0.42163 | p=0.64673 |

*1: major, 2: minor allele
