# Supplementary material for: Roles of Genetic Polymorphisms in the Folate Pathway in Childhood Acute Lymphoblastic Leukemia Evaluated by Bayesian Relevance and Effect Size Analysis
Source: PLoS One. 2013 Aug 5;8(8):e69843. doi: 10.1371/journal.pone.0069843 (PMC3734218; doi:10.1371/journal.pone.0069843)
Supplement: Table S3 — Genotype and minor allele frequencies (MAF) in patient (ALL) and control groups. (DOC) [file pone.0069843.s007.doc]

**Table S**3 Genotype and minor allele frequencies (MAF) in patient (ALL) and control groups

| **Gene** | **SNP** | **Alleles***  **1/2** | **Genotype of ALL patients** | | | | **Genotypes of controls** | | | |
| --- | --- | --- | --- | --- | --- | --- | --- | --- | --- | --- |
|  |  |  | **1/1** | **1/2** | **2/2** | **MAF** | **1/1** | **1/2** | **2/2** | **MAF** |
| ***ABCB1*** | **rs10280101** | A/C | 0.841 | 0.151 | 0.009 | 0.084 | 0.811 | 0.169 | 0.020 | 0.105 |
|  | **rs1202179** | A/G | 0.519 | 0.397 | 0.083 | 0.282 | 0.507 | 0.410 | 0.083 | 0.288 |
|  | **rs2235013** | G/A | 0.271 | 0.477 | 0.252 | 0.491 | 0.248 | 0.490 | 0.261 | 0.507 |
|  | **rs9282564** | A/G | 0.811 | 0.182 | 0.008 | 0.098 | 0.771 | 0.218 | 0.011 | 0.120 |
| ***DHFR*** | **rs11742668** | T/C | 0.887 | 0.104 | 0.010 | 0.062 | 0.858 | 0.138 | 0.004 | 0.073 |
|  | **rs1222809** | T/C | 0.562 | 0.372 | 0.067 | 0.252 | 0.548 | 0.384 | 0.068 | 0.260 |
|  | **rs12517451** | C/T | 0.545 | 0.385 | 0.070 | 0.262 | 0.577 | 0.361 | 0.062 | 0.243 |
|  | **rs1478834** | C/A | 0.550 | 0.382 | 0.068 | 0.259 | 0.541 | 0.388 | 0.072 | 0.266 |
|  | **rs1650723** | G/A | 0.735 | 0.239 | 0.026 | 0.145 | 0.712 | 0.271 | 0.017 | 0.152 |
|  | **rs1677626** | G/C | 0.552 | 0.381 | 0.067 | 0.257 | 0.541 | 0.388 | 0.072 | 0.266 |
| ***FPGS*** | **rs10106** | A/G | 0.392 | 0.446 | 0.163 | 0.385 | 0.390 | 0.461 | 0.149 | 0.379 |
|  | **rs1544105** | G/A | 0.372 | 0.459 | 0.169 | 0.398 | 0.388 | 0.465 | 0.147 | 0.380 |
|  | **rs4451422** | A/C | 0.400 | 0.443 | 0.156 | 0.378 | 0.388 | 0.464 | 0.148 | 0.380 |
| ***GGH*** | **rs10957267** | T/A | 0.732 | 0.240 | 0.028 | 0.148 | 0.712 | 0.267 | 0.021 | 0.155 |
|  | **rs11545078** | C/T | 0.853 | 0.144 | 0.004 | 0.076 | 0.843 | 0.151 | 0.006 | 0.081 |
|  | **rs3780127** | C/T | 0.853 | 0.144 | 0.004 | 0.076 | 0.843 | 0.151 | 0.006 | 0.081 |
|  | **rs719235** | G/T | 0.528 | 0.395 | 0.077 | 0.274 | 0.510 | 0.394 | 0.096 | 0.293 |
| ***GSTP1*** | **rs1695** | A/G | 0.494 | 0.414 | 0.092 | 0.299 | 0.514 | 0.388 | 0.098 | 0.292 |
|  | **rs749174** | C/T | 0.496 | 0.411 | 0.092 | 0.298 | 0.508 | 0.396 | 0.097 | 0.295 |
|  | **rs7941395** | A/G | 0.423 | 0.448 | 0.129 | 0.353 | 0.431 | 0.439 | 0.130 | 0.350 |
| ***MTHFD1*** | **rs1076991** | A/G | 0.241 | 0.517 | 0.241 | 0.500 | 0.338 | 0.488 | 0.174 | 0.418 |
|  | **rs1950902** | C/T | 0.713 | 0.257 | 0.030 | 0.158 | 0.725 | 0.241 | 0.034 | 0.154 |
|  | **rs2236225** | C/T | 0.359 | 0.462 | 0.179 | 0.410 | 0.331 | 0.459 | 0.210 | 0.440 |
|  | **rs745686** | A/G | 0.485 | 0.406 | 0.109 | 0.312 | 0.473 | 0.422 | 0.104 | 0.315 |
| ***MTHFR*** | **rs13306561** | T/C | 0.726 | 0.262 | 0.011 | 0.142 | 0.741 | 0.246 | 0.013 | 0.136 |
|  | **rs1801131** | A/C | 0.488 | 0.422 | 0.090 | 0.301 | 0.501 | 0.403 | 0.096 | 0.298 |
|  | **rs1801133** | C/T | 0.397 | 0.463 | 0.140 | 0.372 | 0.407 | 0.464 | 0.129 | 0.361 |
| ***MTRR*** | **rs10380** | C/T | 0.854 | 0.144 | 0.002 | 0.074 | 0.844 | 0.153 | 0.004 | 0.080 |
|  | **rs1532268** | G/A | 0.389 | 0.466 | 0.145 | 0.378 | 0.450 | 0.425 | 0.125 | 0.337 |
|  | **rs162036** | A/G | 0.813 | 0.183 | 0.004 | 0.095 | 0.805 | 0.183 | 0.011 | 0.103 |
|  | **rs1801394** | G/A | 0.280 | 0.525 | 0.195 | 0.458 | 0.295 | 0.490 | 0.216 | 0.460 |
|  | **rs2966952** | C/T | 0.665 | 0.304 | 0.031 | 0.183 | 0.677 | 0.285 | 0.038 | 0.181 |
|  | **rs326120** | A/G | 0.660 | 0.311 | 0.030 | 0.185 | 0.675 | 0.285 | 0.040 | 0.182 |
|  | **rs3776455** | A/G | 0.428 | 0.472 | 0.100 | 0.336 | 0.410 | 0.420 | 0.170 | 0.380 |
| ***MTR*** | **rs10925257** | A/G | 0.631 | 0.327 | 0.042 | 0.206 | 0.648 | 0.308 | 0.044 | 0.198 |
|  | **rs12759827** | A/G | 0.579 | 0.360 | 0.061 | 0.241 | 0.522 | 0.397 | 0.081 | 0.280 |
|  | **rs1805087** | A/G | 0.633 | 0.322 | 0.044 | 0.206 | 0.645 | 0.308 | 0.047 | 0.201 |
|  | **rs2853523** | C/A | 0.383 | 0.483 | 0.134 | 0.376 | 0.352 | 0.473 | 0.176 | 0.412 |
|  | **rs3768142** | T/G | 0.372 | 0.490 | 0.139 | 0.384 | 0.346 | 0.474 | 0.180 | 0.417 |
|  | **rs4659724** | G/A | 0.378 | 0.497 | 0.125 | 0.373 | 0.415 | 0.467 | 0.118 | 0.351 |
| ***SHMT1*** | **rs1979277** | G/A | 0.480 | 0.434 | 0.087 | 0.304 | 0.503 | 0.408 | 0.089 | 0.293 |
|  | **rs643333** | C/A | 0.498 | 0.420 | 0.081 | 0.292 | 0.537 | 0.391 | 0.072 | 0.267 |
|  | **rs9909104** | T/C | 0.597 | 0.344 | 0.059 | 0.231 | 0.541 | 0.406 | 0.053 | 0.256 |
| ***SLC19A1*** | **rs1051266** | G/A | 0.312 | 0.504 | 0.184 | 0.436 | 0.319 | 0.484 | 0.197 | 0.439 |
|  | **rs4819128** | T/C | 0.335 | 0.481 | 0.184 | 0.424 | 0.342 | 0.473 | 0.184 | 0.421 |
|  | **rs7499** | G/A | 0.340 | 0.482 | 0.178 | 0.419 | 0.385 | 0.463 | 0.152 | 0.383 |
| ***SLC22A8*** | **rs2276299** | A/T | 0.679 | 0.286 | 0.035 | 0.178 | 0.660 | 0.318 | 0.023 | 0.181 |
|  | **rs3809069** | T/C | 0.678 | 0.293 | 0.029 | 0.176 | 0.686 | 0.282 | 0.032 | 0.173 |
|  | **rs4149183** | T/C | 0.580 | 0.374 | 0.046 | 0.233 | 0.626 | 0.324 | 0.049 | 0.212 |
| ***SLC21A6*** | **rs10841769** | G/A | 0.273 | 0.491 | 0.236 | 0.482 | 0.259 | 0.503 | 0.238 | 0.490 |
|  | **rs11045818** | G/A | 0.772 | 0.207 | 0.021 | 0.125 | 0.732 | 0.261 | 0.008 | 0.138 |
|  | **rs11045819** | C/A | 0.781 | 0.203 | 0.016 | 0.117 | 0.732 | 0.260 | 0.008 | 0.138 |
|  | **rs11045823** | G/A | 0.759 | 0.218 | 0.023 | 0.132 | 0.730 | 0.263 | 0.008 | 0.139 |
|  | **rs17328763** | T/C | 0.723 | 0.250 | 0.028 | 0.152 | 0.693 | 0.271 | 0.036 | 0.172 |
|  | **rs4149056** | T/C | 0.661 | 0.304 | 0.035 | 0.187 | 0.660 | 0.295 | 0.045 | 0.193 |
|  | **rs4363657** | T/C | 0.643 | 0.317 | 0.041 | 0.199 | 0.644 | 0.297 | 0.059 | 0.207 |
| ***TPMT*** | **rs2518463** | C/T | 0.237 | 0.511 | 0.252 | 0.507 | 0.285 | 0.480 | 0.235 | 0.475 |
|  | **rs2842951** | C/T | 0.566 | 0.360 | 0.074 | 0.254 | 0.582 | 0.344 | 0.074 | 0.246 |
|  | **rs4449636** | A/G | 0.242 | 0.506 | 0.251 | 0.505 | 0.284 | 0.477 | 0.239 | 0.477 |
| ***TYMS*** | **rs1004474** | A/G | 0.298 | 0.479 | 0.223 | 0.462 | 0.272 | 0.531 | 0.197 | 0.462 |
|  | **rs2612100** | G/A | 0.446 | 0.431 | 0.123 | 0.339 | 0.440 | 0.448 | 0.112 | 0.336 |
|  | **rs2853533** | G/C | 0.717 | 0.251 | 0.031 | 0.157 | 0.779 | 0.202 | 0.019 | 0.120 |
|  | **rs2853741** | C/T | 0.464 | 0.432 | 0.104 | 0.320 | 0.496 | 0.428 | 0.076 | 0.290 |
|  | **rs9967368** | G/C | 0.320 | 0.515 | 0.165 | 0.422 | 0.349 | 0.496 | 0.155 | 0.403 |

*: 1 / 2: major/minor alleles, alleles on the forward strand according to NCBI Genome Build 36.0
