# Supplementary material for: Roles of Genetic Polymorphisms in the Folate Pathway in Childhood Acute Lymphoblastic Leukemia Evaluated by Bayesian Relevance and Effect Size Analysis
Source: PLoS One. 2013 Aug 5;8(8):e69843. doi: 10.1371/journal.pone.0069843 (PMC3734218; doi:10.1371/journal.pone.0069843)
Supplement: Table S4 — Summary of the nominally significant results according to the frequentist analysis. (DOC) [file pone.0069843.s008.doc]

**Table S4 Summary of the nominally significant results according to the frequentist analysis**

| **SNP (rs#)** | **Alleles** | **Overall** | | **B-ALL** | | **T-ALL** | | | **HD-ALL** | |
| --- | --- | --- | --- | --- | --- | --- | --- | --- | --- | --- |
|  |  | p* | OR(95%CI) | p* | OR(95%CI) | p* | OR(95%CI) | | p* | OR(95%CI) |
| **Genotype frequency** | | | | | | | | | | |
| **rs2235013 *(ABCB1)*** | GG | Reference | | | | | | | | |
|  | GA | 0.44 | 0.89(0.66-1.19) | 0.67 | 0.93(0.68-1.29) | **0.04** | **0.57(0.33-0.97)** | | 0.46 | 1.26(0.68-2.34) |
|  | AA | 0.46 | 0.88(0.63-1.23) | 0.71 | 0.93(0.65-1.35) | **0.02** | **0.45(0.23-0.89)** | | 0.44 | 1.31(0.66-2.60) |
| **rs12517451 *(DHFR)*** | CC | Reference | | | | | | | | |
|  | CT | 0.35 | 1.13(0.88-1.45) | 0.29 | 1.16(0.88-1.53) | 0.56 | 1.16(0.70-1.94) | | **0.04** | **1.68(1.03-2.75)** |
|  | TT | 0.49 | 1.19(0.73-1.95) | 0.61 | 1.15(0.67-1.99) | 0.16 | 1.83(0.78-4.28) | | 0.63 | 1.28(0.47-3.49) |
| **rs1544105 *(FPGS)*** | GG | Reference | | | | | | | | |
|  | GA | 0.84 | 1.03(0.79-1.34) | 0.63 | 1.07(0.80-1.43) | 0.19 | 0.70(0.41-1.20) | | 0.06 | 1.71(0.98-2.98) |
|  | AA | 0.35 | 1.19(0.83-1.70) | 0.41 | 1.18(0.80-1.75) | 0.54 | 1.23(0.64-2.36) | | 0.13 | 1.76(0.85-3.63) |
| **rs12759827 *(MTR)*** | AA | Reference | | | | | | | | |
|  | AG | 0.12 | 0.82(0.64-1.05) | 0.13 | 0.81(0.61-1.06) | 0.19 | 0.71(0.42-1.19) | | 0.17 | 0.70(0.42-1.16) |
|  | GG | 0.11 | 0.68(0.42-1.10) | 0.08 | 0.62(0.36-1.06) | 0.77 | 0.87(0.35-2.18) | | 0.24 | 0.53(0.18-1.53) |
| **rs1532268 *(MTRR)*** | GG | Reference | | | | | | | | |
|  | GA | 0.07 | 1.27(0.98-1.64) | 0.19 | 1.21(0.91-1.60) | 0.08 | 1.61(0.95-2.72) | | 0.26 | 1.34(0.81-2.25) |
|  | AA | 0.12 | 1.35(0.93-1.96) | 0.13 | 1.37(0.91-2.06) | 0.31 | 1.48(0.69-3.16) | | 0.46 | 1.32(0.63-2.78) |
| **rs9909104 *(SHMT1)*** | TT | Reference | | | | | | | | |
|  | TC | **0.04** | **0.77(0.59-0.99)** | **0.03** | **0.73(0.55-0.96)** | 0.43 | 0.81(0.49-1.36) | | 0.11 | 0.65(0.38-1.10) |
|  | CC | 0.97 | 1.01(0.59-1.72) | 0.70 | 0.89(0.49-1.61) | 0.28 | 1.64(0.67-4.02) | | 0.35 | 1.53(0.63-3.69) |
| **rs2853533 *(TYMS)*** | GG | Reference | | | | | | | | |
|  | GC | **0.04** | **1.35(1.01-1.81)** | 0.21 | 1.23(0.89-1.69) | **0.02** | **1.85(1.09-3.13)** | | 0.24 | 1.40(0.80-2.43) |
|  | CC | 0.15 | 1.79(0.81-3.96) | 0.24 | 1.68(0.71-3.94) | 0.11 | 2.98(0.77-11.50) | | 0.22 | 2.27(0.60-8.54) |
| **Allele positivity** | | | | | | | | | | |
| **rs2235013 *(ABCB1)*** | GG vs GA+AA | 0.39 | 0.89(0.67-1.17) | 0.65 | 0.93(0.69-1.26) | **0.01** | **0.53(0.32-0.87)** | | 0.41 | 1.28(0.71-2.29) |
| **rs12517451 *(DHFR)*** | CC vs CT+TT | 0.30 | 1.14(0.89-1.45) | 0.27 | 1.16(0.89-1.51) | 0.34 | 1.26(0.78-2.04) | | **0.05** | **1.62(1.01-2.61)** |
| **rs1544105 *(FPGS)*** | GG vs GA+AA | 0.61 | 1.07(0.83-1.37) | 0.49 | 1.10(0.84-1.44) | 0.44 | 0.83(0.51-1.34) | | **0.04** | **1.72(1.01-2.93)** |
| **rs12759827 *(MTR)*** | AA vs AG+GG | 0.06 | 0.79(0.62-1.01) | 0.06 | 0.77(0.59-1.01) | 0.22 | 0.74(0.45-1.20) | | 0.10 | 0.67(0.41-1.09) |
| **rs1532268 *(MTRR)*** | GG vs GA+AA | **0.04** | **1.29(1.01-1.64)** | 0.11 | 1.25(0.95-1.62) | 0.07 | 1.58(0.96-2.61) | | 0.24 | 1.34(0.82-2.18) |
| **rs9909104 *(SHMT1)*** | TT vs TC+CC | 0.06 | 0.79(0.62-1.01) | **0.03** | **0.75(0.57-0.98)** | 0.70 | 0.91(0.56-1.47) | | 0.25 | 0.75(0.46-1.22) |
| **rs2853533 *(TYMS)*** | GG vs GC+CC | **0.02** | **1.39(1.05-1.84)** | 0.13 | 1.27(0.93-1.72) | **0.01** | **1.93(1.16-3.21)** | | 0.15 | 1.47(0.87-2.49) |
| **Allele frequency** | | | | | | | | | | |
| **rs2235013 *(ABCB1)*** | G/A | 0.46 | 0.94(0.79-1.11) | 0.78 | 0.97(0.81-1.17) | **0.01** | | **0.64(0.46-0.91)** | 0.46 | 1.14(.081-1.59) |
| **rs12517451 *(DHFR)*** | C/T | 0.30 | 1.11(0.91-1.35) | 0.33 | 1.11(0.90-1.37) | 0.16 | | 1.30(0.90-1.89) | 0.10 | 1.36(0.94-1.96) |
| **rs1544105 *(FPGS)*** | G/A | 0.39 | 1.08(0.91-1.29) | 0.37 | 1.09(0.90-1.32) | 0.91 | | 1.02(0.72-1.44) | 0.07 | 1.36(0.97-1.91) |
| **rs12759827 *(MTR)*** | A/G | **0.04** | **0.82(0.67-0.99)** | **0.03** | **0.79(0.64-0.98)** | 0.27 | | 0.80(0.54-1.19) | 0.09 | 0.71(0.47-1.06) |
| **rs1532268 *(MTRR)*** | G/A | **0.05** | **1.20(1.00-1.43)** | 0.07 | 1.19(0.99-1.45) | 0.14 | | 1.30(0.92-1.83) | 0.30 | 1.20(0.85-1.70) |
| **rs9909104 *(SHMT1)*** | T/C | 0.18 | 0.87(0.72-1.06) | 0.08 | 0.83(0.67-1.03) | 0.86 | | 1.04(0.71-1.52) | 0.67 | 0.92(0.62-1.36) |
| **rs2853533 *(TYMS)*** | G/C | **0.01** | **1.37(1.07-1.75)** | 1.26 | 0.09(0.96-1.66) | **7E-03** | | **1.82(1.18-2.81)** | 0.10 | 1.45(0.93-2.32) |

*Values reached the p≤0.05 significance threshold are nominally significant and in bold
