# Supplementary material for: Roles of Genetic Polymorphisms in the Folate Pathway in Childhood Acute Lymphoblastic Leukemia Evaluated by Bayesian Relevance and Effect Size Analysis
Source: PLoS One. 2013 Aug 5;8(8):e69843. doi: 10.1371/journal.pone.0069843 (PMC3734218; doi:10.1371/journal.pone.0069843)
Supplement: Table S5 — Linkage disequilibrium coefficients (D' and r2) of the MTHFD1 and MTRR gene polymorphisms. (DOC) [file pone.0069843.s009.doc]

**Table S5 Linkage disequilibrium coefficients (D' and r^2^) of the *MTHFD1* and *MTRR* gene polymorphisms**

| **ALL** | | | | | | **Controls** | | | | |
| --- | --- | --- | --- | --- | --- | --- | --- | --- | --- | --- |
| **Gene** | **SNP ID_L1** | **SNP ID_L2** | **D'** | **LOD** | **r^2** | **SNP ID_L1** | **SNP ID_L2** | **D'** | **LOD** | **r^2** |
| MTHFD1 | rs1076991 | rs1950902 | 0.16 | 0.52 | 0.01 | rs1076991 | rs1950902 | 0.03 | 0.01 | 0.00 |
| MTHFD1 | rs1076991 | rs2236225 | 0.21 | 3.90 | 0.03 | rs1076991 | rs2236225 | 0.26 | 4.98 | 0.04 |
| MTHFD1 | rs1076991 | rs745686 | 0.11 | 0.71 | 0.01 | rs1076991 | rs745686 | 0.14 | 1.34 | 0.01 |
| MTHFD1 | rs1950902 | rs2236225 | 0.42 | 2.93 | 0.02 | rs1950902 | rs2236225 | 0.39 | 3.02 | 0.02 |
| MTHFD1 | rs1950902 | rs745686 | 0.59 | 17.99 | 0.14 | rs1950902 | rs745686 | 0.57 | 16.60 | 0.13 |
| MTHFD1 | rs2236225 | rs745686 | 0.96 | 51.43 | 0.29 | rs2236225 | rs745686 | 0.99 | 64.73 | 0.36 |
| MTRR | rs2966952 | rs1801394 | 1.00 | 46.52 | 0.27 | rs2966952 | rs1801394 | 1.00 | 44.01 | 0.26 |
| MTRR | rs2966952 | rs326120 | 0.99 | 164.12 | 0.98 | rs2966952 | rs326120 | 1.00 | 166.98 | 0.99 |
| MTRR | rs2966952 | rs1532268 | 0.90 | 17.09 | 0.11 | rs2966952 | rs1532268 | 0.98 | 21.25 | 0.11 |
| MTRR | rs2966952 | rs162036 | 1.00 | 4.44 | 0.02 | rs2966952 | rs162036 | 0.78 | 1.92 | 0.02 |
| MTRR | rs2966952 | rs3776455 | 0.96 | 64.19 | 0.42 | rs2966952 | rs3776455 | 0.94 | 50.80 | 0.32 |
| MTRR | rs2966952 | rs10380 | 1.00 | 3.60 | 0.02 | rs2966952 | rs10380 | 0.74 | 1.35 | 0.01 |
| MTRR | rs1801394 | rs326120 | 1.00 | 47.09 | 0.27 | rs1801394 | rs326120 | 1.00 | 44.65 | 0.26 |
| MTRR | rs1801394 | rs1532268 | 0.12 | 0.92 | 0.01 | rs1801394 | rs1532268 | 0.17 | 1.57 | 0.01 |
| MTRR | rs1801394 | rs162036 | 1.00 | 17.63 | 0.13 | rs1801394 | rs162036 | 1.00 | 23.39 | 0.14 |
| MTRR | rs1801394 | rs3776455 | 0.85 | 62.58 | 0.43 | rs1801394 | rs3776455 | 0.76 | 63.58 | 0.41 |
| MTRR | rs1801394 | rs10380 | 1.00 | 11.78 | 0.09 | rs1801394 | rs10380 | 1.00 | 17.12 | 0.10 |
| MTRR | rs326120 | rs1532268 | 0.91 | 17.29 | 0.11 | rs326120 | rs1532268 | 0.95 | 20.24 | 0.10 |
| MTRR | rs326120 | rs162036 | 1.00 | 4.24 | 0.02 | rs326120 | rs162036 | 0.79 | 2.02 | 0.02 |
| MTRR | rs326120 | rs3776455 | 0.96 | 65.24 | 0.43 | rs326120 | rs3776455 | 0.93 | 49.85 | 0.31 |
| MTRR | rs326120 | rs10380 | 1.00 | 3.38 | 0.02 | rs326120 | rs10380 | 0.74 | 1.38 | 0.01 |
| MTRR | rs1532268 | rs162036 | 1.00 | 10.45 | 0.06 | rs1532268 | rs162036 | 1.00 | 9.71 | 0.06 |
| MTRR | rs1532268 | rs3776455 | 0.99 | 51.63 | 0.30 | rs1532268 | rs3776455 | 0.98 | 55.29 | 0.30 |
| MTRR | rs1532268 | rs10380 | 1.00 | 8.27 | 0.05 | rs1532268 | rs10380 | 1.00 | 7.94 | 0.05 |
| MTRR | rs162036 | rs3776455 | 1.00 | 28.24 | 0.20 | rs162036 | rs3776455 | 1.00 | 31.45 | 0.19 |
| MTRR | rs162036 | rs10380 | 1.00 | 75.93 | 0.77 | rs162036 | rs10380 | 1.00 | 80.66 | 0.79 |
| MTRR | rs3776455 | rs10380 | 1.00 | 20.85 | 0.16 | rs3776455 | rs10380 | 1.00 | 24.19 | 0.15 |
