# Supplementary material for: Roles of Genetic Polymorphisms in the Folate Pathway in Childhood Acute Lymphoblastic Leukemia Evaluated by Bayesian Relevance and Effect Size Analysis
Source: PLoS One. 2013 Aug 5;8(8):e69843. doi: 10.1371/journal.pone.0069843 (PMC3734218; doi:10.1371/journal.pone.0069843)
Supplement: Table S7 — Structural features of different dependence types between variables. (DOC) [file pone.0069843.s011.doc]

**Table S7 Structural features of different dependence types between variables**

| **Relation** | **Abbreviation** | **Graphical** |
| --- | --- | --- |
| **Pairwise features** |  |  |
| Direct causal relevance | DCR(X,Y) | There is an edge between X and Y |
| Transitive causal relevance | TCR(X,Y) | There is directed path between X and Y |
| Confounded relevance | ConfR(X,Y) | X and Y have common ancestor |
| Association | A(X,Y) | DCR or TCR or ConfR |
| Pure interactionist relevance | PIR(X,Y) | X and Y have common child |
| Strong relevance | SR(X,Y) | PIR or DCR |
| **Relevance of variable sets** |  |  |
| Strong relevance | MBS(Y) | The set consisting of Y’s parents, its children, and the other parents of its children (the Markov Blanket Set of Y) |
| **Interaction models of relevant variables** | | |
| Strong relevance | MBG(Y) | The subgraph that includes the nodes in the MBS and the incoming edges into Y and into its children (the Markov Blanket Subgraph of Y) |
