# Supplementary material for: Roles of Genetic Polymorphisms in the Folate Pathway in Childhood Acute Lymphoblastic Leukemia Evaluated by Bayesian Relevance and Effect Size Analysis
Source: PLoS One. 2013 Aug 5;8(8):e69843. doi: 10.1371/journal.pone.0069843 (PMC3734218; doi:10.1371/journal.pone.0069843)
Supplement: Table S9 — Joint effect of TYMS rs1004474 and MTRR rs1532268 according to the Bayesian effect size analysis. (DOC) [file pone.0069843.s013.doc]

Table S9 Joint effect of *TYMS* rs1004474 and *MTRR* rs1532268 according to the Bayesian effect size analysis

| ***TYMS* rs1004474** | ***MTRR*** **rs1532268** | **Conditional probability of risk of  HD-ALL** | **Joint OR^a^** |
| --- | --- | --- | --- |
| AA | GG | 0.23 | 1.00 |
| AA | AG or AA | 0.09 | 0.32 |
| GA or GG | GG | 0.08 | 0.29 |
| GA or GG | AG or AA | 0.17 | 0.70 |

^a^Joint OR – Joint odds ratio, computed using the common homozygous
case for both SNPs as a basis
